# Assessing the current and future potential geographic distribution of the American dog tick, *Dermacentor variabilis* (Say) (Acari: Ixodidae) in North America

**DOI:** 10.1371/journal.pone.0237191

**Published:** 2020-08-10

**Authors:** Gunavanthi D. Y. Boorgula, A. Townsend Peterson, Desmond H. Foley, Roman R. Ganta, Ram K. Raghavan

**Affiliations:** 1 Department of Diagnostic Medicine and Pathobiology, College of Veterinary Medicine, Kansas State University, Manhattan, Kansas, United States of America; 2 Department of Ecology and Evolutionary Biology, College of Liberal Arts and Sciences, The University of Kansas, Lawrence, Kansas, United States of America; 3 Walter Reed Biosystematics Unit, Department of Entomology, National Museum of History, Washington, District of Columbia, United States of America; 4 Center for Vector-borne and Emerging Infectious Diseases, Departments of Veterinary Pathobiology and Public Health, College of Veterinary Medicine and School of Health Professions, University of Missouri, Columbia, South Carolina, United States of America; Instituto Federal de Educacao Ciencia e Tecnologia Goiano - Campus Urutai, BRAZIL

## Abstract

The American dog tick, *Dermacentor variabilis*, is a veterinary- and medically- significant tick species that is known to transmit several diseases to animal and human hosts. The spatial distribution of this species in North America is not well understood, however; and knowledge of likely changes to its future geographic distribution owing to ongoing climate change is needed for proper public health planning and messaging. Two recent studies have evaluated these topics for *D*. *variabilis*; however, less-rigorous modeling approaches in those studies may have led to erroneous predictions. We evaluated the present and future distribution of this species using a correlative maximum entropy approach, using publicly available occurrence information. Future potential distributions were predicted under two representative concentration pathway (RCP) scenarios; RCP 4.5 for low-emissions and RCP 8.5 for high-emissions. Our results indicated a broader current distribution of this species in all directions relative to its currently known extent, and dramatic potential for westward and northward expansion of suitable areas under both climate change scenarios. Implications for disease ecology and public health are discussed.

## 1. Introduction

*Dermacentor variabilis* (Say) (Acari: Ixodidae), commonly referred as the American dog tick or wood tick is a broadly distributed tick species throughout central and eastern North America [1,2], extending north to southern Canada east of Saskatchewan [3], and ranging in California along west of the Cascade and Sierra Nevada mountain ranges [2,4], and reaching to Mexico to the south [5]. The medical and veterinary significance of *D*. *variabilis* is well documented (e.g., Winer and Strakosch, 1941; Kocan et al., 1981; Schriefer and Azad, 1994) [6–8], and incidence rates of some of the diseases vectored by *D*. *variabilis* appear to have worsened over recent decades in the US. Year-to-year and county-to-county increase in incidence of Rocky Mountain spotted fever (RMSF) [9] and bovine anaplasmosis cases [10] are just two examples of this trend. Even though RMSF is reported along with other spotted fever group rickettsioses, which are likely transmitted by other ticks and mites, the most serious and commonly reported spotted fever group rickettsiosis (SFGR) in the U.S is RMSF [11]. The reasons for these trends could be an increased abundance of *D*. *variabilis* in the environment and subsequent higher human contact rates with these ticks, and potentially also the expansion and/or shifts in its geographic distribution. Changes to the spatial distribution of *D*. *variabilis*, in terms of an increase in geographic area or a shift in distribution can have direct implications on the burden of diseases transmitted by this species, as well as exert severe economic impacts due to an increase in production losses attributable to bovine anaplasmosis. For instance, the local maintenance of *Anaplasma marginale*, the causative agent of bovine anaplasmosis along the current northern distributional extent that are currently free of *D*. *variabilis*, such as Manitoba may permanently change the ecology of this disease [12].

Spatial distributions of ticks are influenced by many factors, viz., land cover/landscape, host availability, host density and dispersal, and vegetation all modulate the spatial dynamics of ticks [13,14]. As ticks are ectothermic, hematophagous arthropods, their life-cycle, phenology, and spatial distribution are largely influenced by ambient temperature and other abiotic and biotic environmental conditions. Different species of ticks require unique but optimal weather conditions to start and finish their life cycle, to start looking for blood-meal hosts (questing behavior), and to transmit pathogens without killing their hosts [15]. It has also been known that other biological functions of a tick, such as reproduction [16] and ability to survive [17] depend on optimal weather conditions. Changes to the abiotic determinants of tick biology is likely to affect the way they adapt to newer environments and subsequently affect tick-borne disease ecology.

Recent changes in temperature and humidity profiles for different regions of the world are attributed to climate change (https://www.ipcc.ch/site/assets/uploads/2018/02/SYR_AR5_FINAL_full.pdf). For this reason, ongoing climate change is suggested already to have caused [e.g., 18], or is anticipated to cause in the near future, range shifts [e.g., 19,20], range expansions [21], and increased in incidence of tick-borne diseases worldwide [22]. Naturally, such changes have direct implications for public health, and, in the case of *D*. *variabilis*, losses in bovine production in the hundreds of millions of dollars every year in the U.S alone. The American dog tick is presently implicated in the transmission of RMSF, tularemia [23], and bovine anaplasmosis [7]. Reports indicate that these ticks may also carry *Anaplasma phagocytophilum*, which causes human granulocytic ehrlichiosis, and *Ehrlichia chaffensis* that causes human monocytic ehrlichiosis but further studies are essential to prove vector-competence [24]. Public health practice relies on accurate and up-to-date information on vector distribution patterns at fine spatial scales for efficient planning and resource allocation.

Species distribution modeling using presence-only data allows us to predict present and future distributions of ticks [e.g., 25,26], and to a limited extent also their potential abundances, which can be useful information in public health settings. A general view of the geographic distribution of *D*. *variabilis* is given in the form of a map by CDC [2]; however, the methods used in developing that are not available in detail, so its accuracy cannot be ascertained. Two recent studies used ecological niche modeling approach to determine the habitat suitability and potential distribution of *D*. *variabilis* in the United States; (James et al., 2015) [27] and for North America by (Minigan et al., 2017) [28]. These studies, however, have not taken advantage of some of the current recommendations for niche modeling that have significant implications for the reliability of predictions. Briefly, these studies did not adequately rarefy occurrence locations, consider an accessible area (**M**) for the species in calibrating models [29,30] did not adequately explore the complexity of covariate relationships with occurrence data [31], used AICc and/or AUC criteria alone for model selection, which is problematic [32,33]. Also, the extent to which their models are affected by explicit extrapolation alone, which can be evaluated using the Mobility-oriented Parity (MOP) metric [34], is not clear. We recently showed that neglect of some or all these issues could lead to biased distributional estimates for a different tick species, *Ixodes scapularis* [25,26].

The purpose of this study was to evaluate the spatial distribution of *D*. *variabilis* in North America under present-day and future climatic conditions using presence-only occurrence data. We used Maxent, a widely utilized approach among ecologists and public health researchers for species distribution modeling in this study. One could expect variations in the spatial distribution of *D*. *variabilis* on the use of a different model algorithm, the evaluation of which was beyond the scope of this study. We simulated future potential distributions under low- and high- greenhouse gas emission scenarios to account for uncertainties in projections of climate change. Our results indicate potential distribution of this medically important tick species in N. America is broader than the species’ currently assumed range, with different levels of suitability within its range; and, *D*. *variabilis* is likely to expand in to newer territories in the coming decades under both low and high emissions climate change scenarios.

## 2. Materials and methods

### 2.1 Occurrence data

Occurrence data for *D*. *variabilis* were obtained from the Walter Reed Bio-systematics Unit (WRBU), a unique national resource for systematics research on medically important arthropods and maintenance of the U.S. national mosquito collection [35]. This collection also includes tick specimen records for North America and other parts of the world. Records in the WRBU database included specimens submitted by 9 institutions, Australian museum (1), Berkeley Natural History Museum (22), Illinois Natural History Survey (308), Ohio State University Acarology Collection (373), The University of Arizona School of Geography and Development (13), U.S Army Institute of Public Health (5114), Uniformed Services University of the Health Sciences (9), University of Alberta, Entomology Collection (276), and Walter Reed Biosystematics Unit (108). The earliest specimen in the record was collected in July of 1898 submitted by Ohio State University Acarology Collection. There were 2228 specimens submitted between years 1898 and 1999, and 3967 specimens collected between 2000 to 2014. Of these, 1690 were between 2010 and 2014. Twenty-nine records lacked collection date. Five thousand one hundred and thirty-seven specimens were based on human observations, which were likely ticks attached to humans, 1016 were preserved specimens, which likely included field collections of questing ticks as well as human and animal host-attached ticks. The source of 71 specimens were not recorded. Quality control filters were applied to this dataset in sequential steps. First, data records with inadequacies (misspelled species names, no location information, or uncertainty information, and records with uncertainty >10,000 m) were excluded. Verbal descriptions of occurrence locations had previously been converted by WRBU to geographic coordinates following the Biogeomancer (http://www.biogeomancer.org) and Mammal Networked Information System (MANIS) protocol (http://www.manisnet.org). It is not uncommon for museum records such as those available from WRBU to include spatial biases, with some regions being better represented than others. To remove such bias, multiple occurrences at the same location were removed, followed by removal of occurrence points falling within 10,000 m from each other; one from each such pair was removed in random order using the Spatial Analyst Toolbox in ArcGIS. The occurrence data thus rarefied were then randomly divided into two equal sets, one for calibrating models, and the other for evaluating model performances. The geographic latitude and longitude coordinates of rarefied occurrences are present in (S1 File).

**Fig 1:**
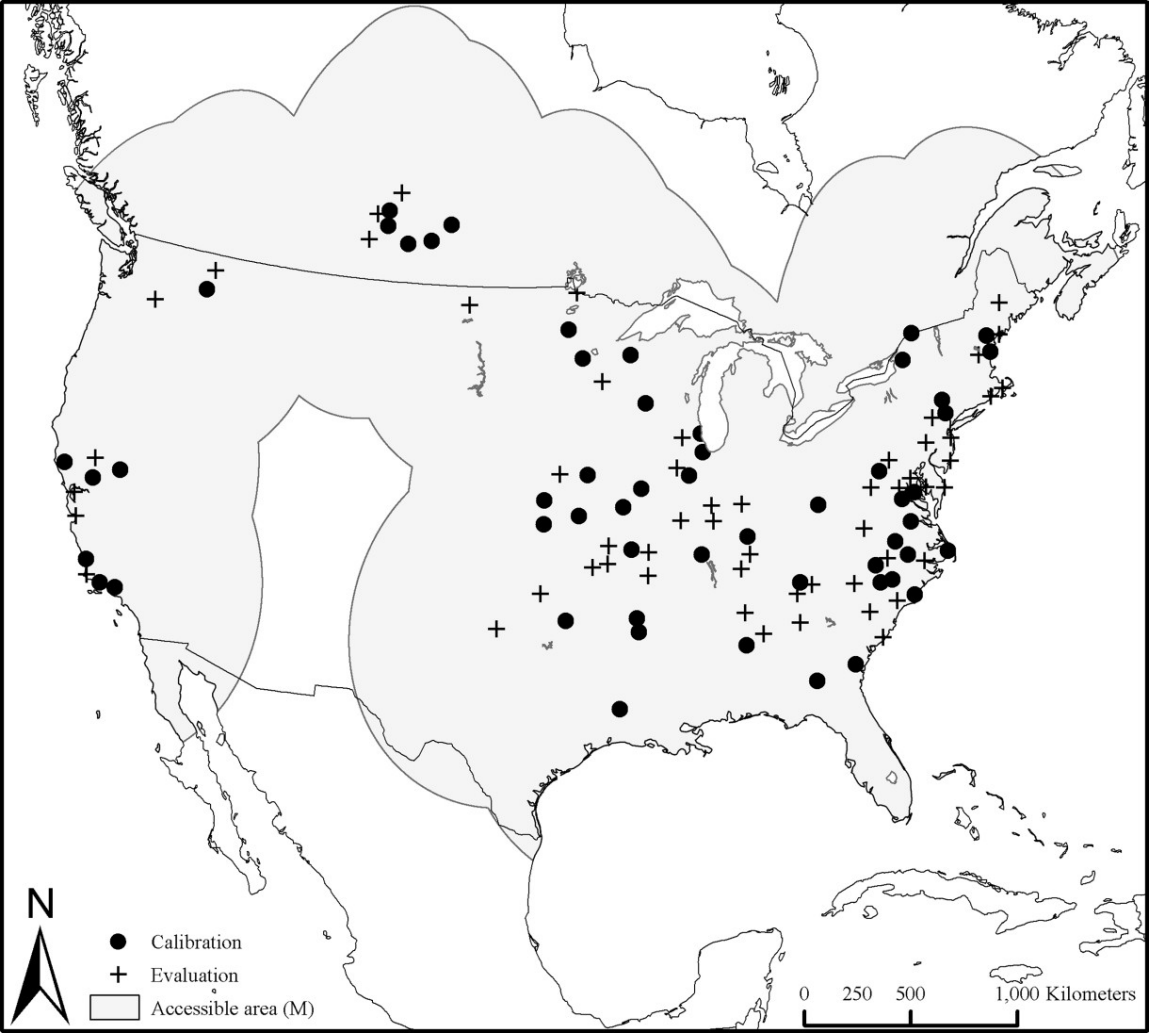
Occurrence locations of *Dermacentor variabilis* used for calibrating and evaluating ecological niche models, and the accessible area, M used in the study.

A search for *D*. *variabilis* records at WRBU archive yielded 6224 records, each representing a location at which one or more *D*. *variabilis* ticks of any of the three post-emergent life stages was collected. Among these records, the species’ name was spelled in two different ways: *variabilis* (correct) and *variablis* (incorrect); We corrected the latter to *D*. *variabilis*. Following removal of data records without location information and records with uncertainty measures >10,000 m, 2956 occurrence locations were available for analysis. Filtering data to 10 km separations, 109 occurrence data remained fit for ecological niche modeling. These steps helped reduce problems associated with artificial clustering of occurrence locations related to biases in sampling and reporting (Peterson and Raghavan 2017a, 2017b) [25,26]. The occurrence data were randomly divided equally into calibration and evaluation datasets (Fig 1). The hypothesized accessible area for *D*. *variabilis*, **M** is depicted in Fig 1.

*Dermacentor variabilis* is established in Mexico [36,37]. Our search for occurrence data in public data repositories *viz*., Global Biodiversity Information Facility (GBIF) (GBIF.org, 2016), species Link (http://www.splink.org.br/index?lang=en), and REMIB (http://www.conabio.gob.mx/remib_ingles/doctos/remib_ing.html) in Mexico and Central American countries did not return results. A recent publication [5] documented extensive historic observations on the presence of different *Dermacentor* spp. in Mexico, including some new records of *D*. *variabilis* collected from dog, bobcat, bovine and wildcat hosts, in three states, Coahuila, Nuevo Leon, and Tamaulipas. However, uncertainties associated with these occurrences could not be verified, and therefore they were not used in calibrating the models in this study.

Based on the rarefied occurrence locations, the accessible area (**M**) for *D*. *variabilis* was estimated using a 7.5 circular buffer surrounding each location in ArcGIS. Briefly, accessible area, **M** is the area that has been accessible to a species by dispersal over relevant time periods [29,30]. This area is the appropriate area for model calibration, testing and comparison [39].

### 2.2 Environmental data

We used a climatic dataset from WorldClim [38] at 30” spatial resolution for model development. WorldClim is derived from historical (1950–2000) monthly temperature and rainfall data to generate biologically meaningful variables. Of the 19 bioclim variables, mean temperature of the wettest quarter (bioclim 8), mean temperature of the driest quarter (bioclim 9), precipitation of the warmest quarter (bioclim 18), precipitation of the coldest quarter (bioclim 19) were *a priori* not considered because they include known spatial artifacts [39]. The jackknife procedure identified climatic variables contributing to shaping the spatial distribution of *D*. *variabilis*. The jackknife manipulation revealed minimum temperature seasonality, temperature annual range, and mean temperature of coldest quarter were the least contributors. These variables were removed and the model refit; the resulting model revealed minimum temperature of the coldest month, precipitation of the driest quarter, and mean temperature of the warmest quarter had low contributions. In a further step, precipitation of wettest month, annual precipitation, and isothermality were least contributors. Further jackknife iterations did not identify any low-contributing variables, so we used the final three sets of environmental variables in our model selection efforts. The jackknife plots used for variable selection and the three variable sets used for model evaluation are present in (S1 File). The final model that met all three statistical criteria included bioclimatic variables in Set-3, regularization multiplier value of 4. This model was constructed with quadratic, threshold and hinge feature classes. The performance statistics for the final model were: mean AUC value = 1.67, partial ROC = 0, omission rate (5%) = 0.03, AICc = 2173.56, ∂AIC = 0, and WAICc = 0.99. Detailed model evaluation results are present in (S2 and S3 Files).

For estimating future distributional potential of *D*. *variabilis*, data representing future climate conditions in year 2050 were downloaded from the Climate Change, Agriculture and Food Security (CCAFS)—Climate data portal (http://ccafs-climate.org/). To account for uncertainty in future climate, we considered two representative concentration pathways, RCP 4.5 and RCP 8.5, that correspond to the lower and higher greenhouse gas emissions scenarios, respectively. Under both scenarios, we considered four general circulation models (GCMs) representing different simulations of global climate dynamics CSIRO MK3, MIROC, NCAR CCSM4, and CCCMA CANESM2. Data for these models were downloaded in ASCII grid format for the North American region at 30” spatial resolution.

### 2.3. Ecological niche modeling

In view of the presence-only nature of the occurrence data that were available for this study, we used MaxEnt (version 3.3.3k) [40], which has design features that are particularly in accord with such data to create models [41]. One could expect distinct estimates of the geographic distribution of *D*. *variabilis* were a different model algorithm to be used, but we focused on Maxent because the assumptions of the approach were most appropriate. The software allows users to specify different model parameters; the caveats and the need for careful consideration of these options have been pointed out [31]. In particular, the feature type options represent mathematical transformation of environmental data allowing complex relationships [42], and the regularization multiplier, which determines the complexity or generality of the modeled response, in model calibration. Further, the number and the types of environmental covariates influence models.

Contributions of different covariates to model performance were determined using jackknife procedures in Maxent. Starting from a model based on 15 BioClim covariates, we constructed several models with progressively fewer covariates, each time removing the covariate or covariates that contributed least to model gain (a measure of fitness) in the previous step. Covariates in the final three jackknife iterations were kept as three sets of covariates (set 1, set 2, and set 3) for evaluating model performance.

In all, we explored and tested a total of 1479 models with different settings of regularization multiplier, response type, and environmental data set using the KUENM package in R-Statistical Program [43]. This included a combination of 17 regularization multipliers, 29 feature classes, and 3 distinct sets of environmental variables. Models were thresholded using a fixed, allowable omission error rate of E = 10% [44]. This trimming allows 10% of records (both occurrence and environmental data) with the lowest suitability, representing potential errors to be omitted without affecting results [44]. We used three model selection criteria to choose an adequate model; first, only models with significant partial ROC value (< 0.05) were considered, followed by models with an omission rate (OR) </ = 10%. Among the significant, low-omission models, we identified the model with lowest AICc value (i.e., least complex) and included all models within 2 AICc units from the least complex model. The median of these model outputs (predicted suitability) was used as the basis of interpretations.

The best model or models was then transferred to future climate scenarios (two RCP scenarios and four GCMs), and these models were replicated 10 times with the bootstrap function. The median of medians (predicted suitability) was used to interpret future potential geographic distributions of *D*. *variabilis* under different climate-change scenarios.

In a final step, the mobility-oriented parity (MOP) metric [34] was calculated in order to determine the novelty of future climate conditions relative to present-day conditions in the calibration area. MOP analysis helps reveal areas where strict extrapolation (i.e., transfer areas with values outside the range of climates in the calibration area) occurs. Areas with higher extrapolative values indicate higher uncertainty; and caution is required when interpreting likelihood of species presence in such areas [45,46].

## 3. Results

**Fig 2:**
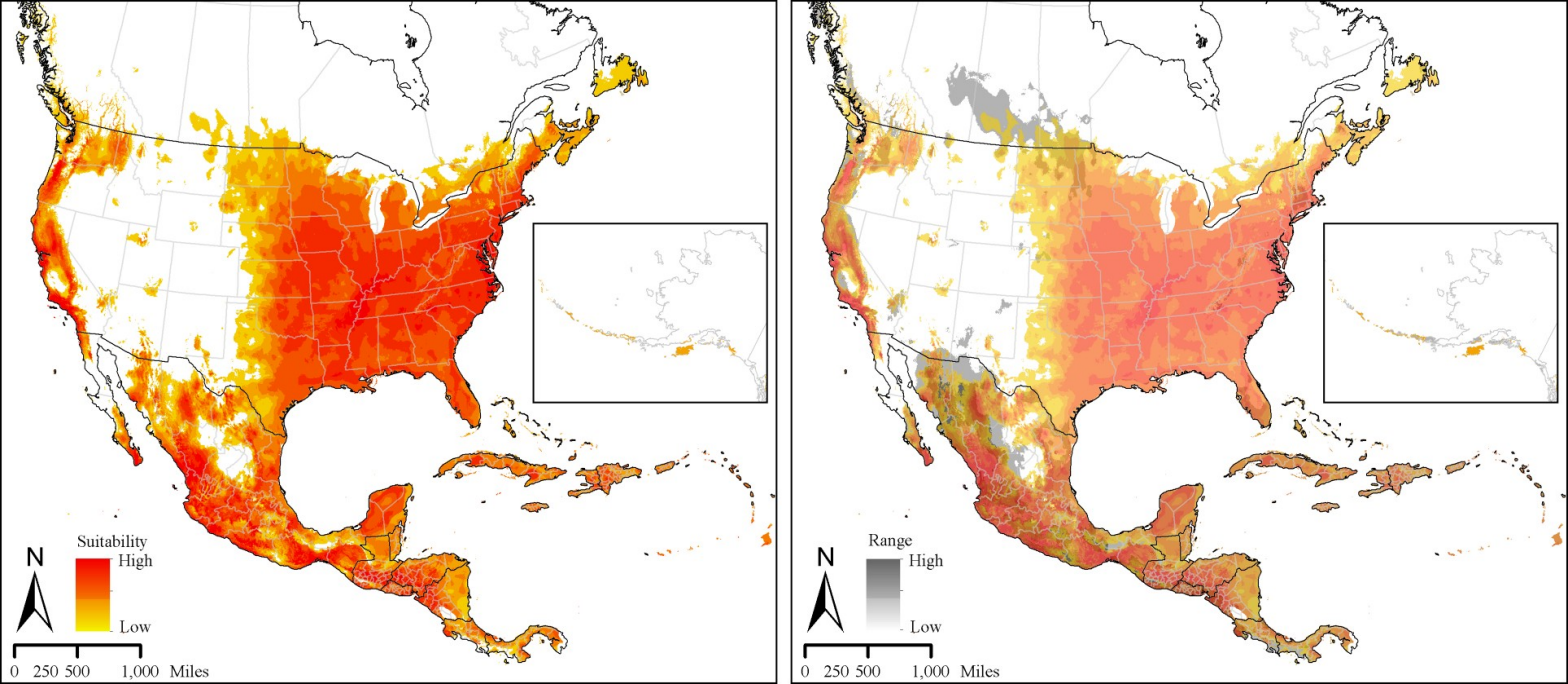
A. Prediction of bio-climatically suitable areas for *Dermacentor variabilis* ticks in North America. B. Uncertainty (range = maximum–minimum suitability value) associated with the prediction of suitable areas for *Dermacentor variabilis* distribution in North America.

The spatial distribution of *D*. *variabilis* based on the median of 10 individual replicate models and associated uncertainty in model predictions, are presented in Fig 2A and 2B. Predicted suitability patterns indicate that the entire eastern half of the United States Central Plains east to the east coast, and most areas along the west coast are also highly suitable. Southern Canada, most of Mexico, and parts of Central America the Caribbean are also climatically suitable for this tick species. Model uncertainty does not raise serious concerns for much of the United States but medium to low level uncertainty can be observed was higher on the west coast, in Saskatchewan and Manitoba in Canada, and in central Mexico (Fig 2B). Annual mean temperature and precipitation of wettest quarter together accounted for more than 74% of the variation in the models, annual precipitation contributed 16.3% and the remaining variables contributed < 4% each. Contribution by individual variables to the final model, their permutation importance and response curves are present in (S4 File).

**Fig 3:**
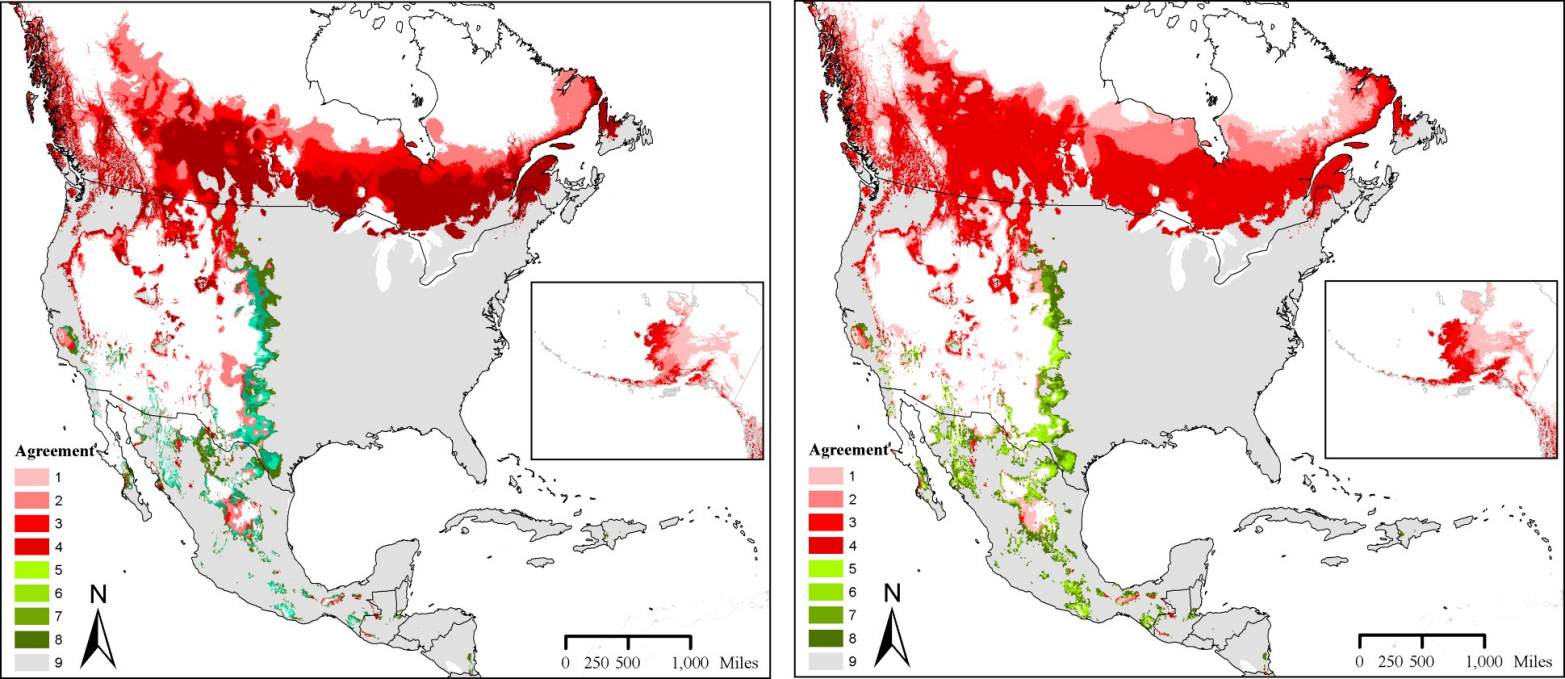
A. Predicted distribution of suitable regions under the Representative Concentration Pathway (RCP) 4.5, and agreement between different Global Circulation Models. B. Predicted distribution of suitable regions under the Representative Concentration Pathway (RCP) 8.5, and agreement between different Global Circulation Models.

Predicted future potential distribution of *D*. *variabilis* under RCP 4.5 (Fig 3A) and RCP 8.5 (Fig 3B) indicated potential for further changes in the spatial distribution in different areas in N. America. Suitability for *D*. *variabilis* generally expanded northward from present-day distribution. Some loss in *D*. *variabilis* distribution relative to the present-day distribution could be noted along the western range limit. Results were similar for the four GCMs under RCP 8.5 scenario, but future suitability expanded much further into the Central Plains and Northern Canada. The MOP analysis did not reveal any areas that would confront problems with model extrapolation.

1 = One of the future scenario models predicted suitability for *Dermacentor variabilis* distribution. 2, 3, 4 = two, three and four models, respectively, predicted suitability for *Dermacentor variabilis* distribution. 5 = One of the models predicted loss of territory, 6, 7, 8 = two, three and four models simulated predicted loss of territory for *Dermacentor variabilis* compared to present-day distribution represented in grey).

## 4. Discussion

It has been known for quite some time that *D*. *variabilis* is one of the most medically significant tick species that transmit disease-causing pathogens to humans and animals in North America [e.g., 47,48]. Spatiotemporal patterns of county-level incidences for some of the diseases transmitted by this tick species, tularemia [49], bovine anaplasmosis [10], and Rocky Mountain spotted fever (RMSF) [9] have worsened steadily over the past decade, indicating a steady increase in disease burden likely related to this tick species. Such increases in incidences could be related to several factors: better disease surveillance programs at state and local levels, expansion of non-Lyme tick-borne diseases on the Centers for Disease Control and Prevention (CDC)’s reportable diseases list, improved diagnostic methods, and increased awareness of, and interest in tick-borne diseases among physicians and patients, to name a few. An important factor, however, is the likely increase in the abundance of *D*. *variabilis* ticks across its distribution, and the potential expansion and shifts in its distribution owing to non-stationary forces such as climate change and exurbanization. Currently, however, only limited information is available on this topic.

The present-day model developed in this study (Fig 2A), which used occurrence data from WRBU alone, differs from currently available predictions for *D*. *variabilis* suitability areas across N. America. In general, our model indicated more suitability westward in the Great Plains than CDC (2018) [2], James et al., (2015) [27] and Minigan et al., (2017) [28]. Additionally, these studies indicated vast areas within the *D*. *variabilis* range as low-suitable and unsuitable, where our model revealed a more continuous but diffused pattern of suitability. The most striking difference in the predictions between the present study and Minigan et al., (2017) [28] however, is that they determined most of Mexico as unsuitable for *D*. *variabilis*, except for narrow areas along the southeastern Texas border, despite including occurrence locations from Mexico in that study. It is likely that their suitability predictions were disproportionately influenced by the presence of large clusters of occurrence locations, particularly in southeast Texas, Florida peninsula, and southern Saskatchewan/Manitoba.

The present-day distribution of *D*. *variabilis* in the current study is similar to, but broader than the generally assumed distribution for this species by the CDC, particularly along the coast of western US and the interiors of the states of Oregon and Washington. Like CDC’s distribution map, the present-day distribution in this study indicates suitable regions for this species in two fronts, one covering the relatively densely populated eastern half of the US, and a second relatively narrow front along the western coast and the interiors of northwestern states, which overlaps the home range of the Rocky Mountain wood tick, *D*. *andersoni* in some areas. The niche suitability for this species gradually decreases along the western edge of its eastern front (Fig 2A); however, much of the areas covering the Central Plains appears suitable to various degrees for the establishment of this species. The Central Plains states of Texas, Oklahoma, Kansas as well as their northern neighbors are major beef cattle producers in the US, which are likely to suffer losses as *D*. *variabilis* further establishes.

The spatial distribution of *D*. *variabilis* determined in this study is largely affected by three climate parameters; annual mean temperature, precipitation of wettest quarter, and annual precipitation, in that order. No other ecological variables such as host abundance, and host density were included nor available at a desirable spatial resolution when building these models. Ticks are exposed to the environment during most of their life-cycle, and they are dependent on a complex combination of climate conditions for their survival and other physiological functions; and, it is widely known that different tick species have specific climate preferences [13,50]. Niche models built using climate variables alone, as in this study, and the climate variable associations found with tick spatial distributions however, must be interpreted with caution [13] as they are more likely to represent areas that are climatically restrictive (climatological niche) and do not include other ecological factors that shape the potential spatial distributions. It is likely that annual mean temperature and precipitation may act as limiting factors for *D*. *variabilis*, and there is potentially an upper and lower limit for these two parameters beyond which physiological processes and life-cycle attributes of these ticks may be affected. Additionally, precipitation during wettest quarter is likely to indirectly affect tick life-cycle by vegetation changes, host availability and abundance and microclimate effects that depend on precipitation, which could affect egg and larval development.

The present-day model in this study when projected into the future (year 2050) indicated marked expansion of suitable areas for *D*. *variabilis* in North America. All eight future-climate scenarios showed a slight eastward retraction of suitability along the western edge of present distribution, however, the model anticipated striking expansion of suitable areas for this species northwards into Canada (Fig 3A and 3B). The potential for northward expansion of *D*. *variabilis* is consistent with the expected distributional shifts of other tick species in North America [e.g., 19] and Europe [e.g., 18], an effect largely attributed to shifting climate patterns. Climate-change influences tick biology and spatiotemporal distribution, and interactions with intermediate hosts and pathogens therefore must be taken into consideration in anticipating tick-borne disease dynamics [50–53] for proper longer-term public health decisions. Studies of such climate change influences relevant to tick-borne disease ecology in North America are severely lacking at present time. It is important to note that we used environmental data for future climate conditions in CCAFS that were potentially downscaled from WorldClim version 1.4, while the niche models for present-day conditions were built using version 2.0 of the same data. Therefore, there is a possibility for some error in our future predictions. However, both datasets are based on a broadly overlapping set of weather station data, such that the differences overall between them are likely to be minor.

Present-day suitability for *D*. *variabilis* ticks as well as changes in suitability resulting from climate change indicate conditions of concern for public health and potential further losses in bovine production. Climate is well-known as a major structuring force in species’ distributional ecology [53]; although other factors certainly also enter the picture (e.g., land use, introductions, host distributions), a reasonable assumption is that climate is an important structuring force. Precise estimates of potential economic losses due to bovine anaplasmosis in the U.S are not readily available. However, older estimates of the cost of a clinical case due to this disease is estimated to be around $400/animal [54,55], and the total economic impact is roughly estimated to be around $300 million/year [56]. The cases of diseases transmitted by *D*. *variabilis*, particularly RMSF has steadily increased over the years (e.g., Raghavan et al., 2016a; CDC) [9,11]. The monetary losses associated with RMSF is likely to be in the millions of dollars and not readily available. Deaths due to RMSF in the U.S are around 2% even with appropriate and timely treatment [11].

The future suitability maps presented here indicate potential areas in which *D*. *variabilis* will be able to establish population if they are able to disperse there. More importantly, the present-day and future predictions here are not indications of the abundance and/or density of ticks present in these areas, but rather only the likelihood of conditions being suitable. Further, tick distribution and infection prevalence with different pathogens are highly spatially heterogeneous [57–59]. Therefore, further studies are needed that address local variation in densities, contact rates with humans and bovine, infection prevalence and host associations of *D*. *variabilis* in areas that are highly suitable and central for this species, as well as the leading edge of its distribution where it may occur at low densities or it may be recently established. Finally, it is important to consider that choice of model algorithm, in addition to data quality and parameter value selection, is an important source of uncertainty in species distribution modeling outcomes.

## Supporting information

S1 File (CSV)

S2 File (DOCX)

S3 File (ZIP)

S4 File (DOCX)
