# Supplementary material for: Assessing the current and future potential geographic distribution of the American dog tick, Dermacentor variabilis (Say) (Acari: Ixodidae) in North America
Source: PLoS One. 2020 Aug 10;15(8):e0237191. doi: 10.1371/journal.pone.0237191 (PMC7416948; doi:10.1371/journal.pone.0237191)
Supplement: S2 File — (DOCX) [file pone.0237191.s002.docx]

**Bioclimatic Variables:**

BIO1 = Annual Mean Temperature
BIO2 = Mean Diurnal Range (Mean of monthly (max temp - min temp))
BIO3 = Isothermality (BIO2/BIO7) (* 100)
BIO4 = Temperature Seasonality (standard deviation *100)
BIO5 = Max Temperature of Warmest Month
BIO6 = Min Temperature of Coldest Month
BIO7 = Temperature Annual Range (BIO5-BIO6)
BIO8 = Mean Temperature of Wettest Quarter
BIO9 = Mean Temperature of Driest Quarter
BIO10 = Mean Temperature of Warmest Quarter
BIO11 = Mean Temperature of Coldest Quarter
BIO12 = Annual Precipitation
BIO13 = Precipitation of Wettest Month
BIO14 = Precipitation of Driest Month
BIO15 = Precipitation Seasonality (Coefficient of Variation)
BIO16 = Precipitation of Wettest Quarter
BIO17 = Precipitation of Driest Quarter
BIO18 = Precipitation of Warmest Quarter
BIO19 = Precipitation of Coldest Quarter

**Table 1. Jackknife variable evaluations:**

| Step | Variable | Removed variables |
| --- | --- | --- |
| Step 1 | BIO1 = Annual Mean Temperature BIO2 = Mean Diurnal Range (Mean of monthly (max temp - min temp)) BIO3 = Isothermality (BIO2/BIO7) (* 100) BIO4 = Temperature Seasonality (standard deviation *100) BIO5 = Max Temperature of Warmest Month BIO6 = Min Temperature of Coldest Month BIO7 = Temperature Annual Range (BIO5-BIO6) BIO10 = Mean Temperature of Warmest Quarter BIO11 = Mean Temperature of Coldest Quarter BIO12 = Annual Precipitation BIO13 = Precipitation of Wettest Month BIO14 = Precipitation of Driest Month BIO15 = Precipitation Seasonality (Coefficient of Variation) BIO16 = Precipitation of Wettest Quarter BIO17 = Precipitation of Driest Quarter |  |
|  | BIO1 = Annual Mean Temperature BIO2 = Mean Diurnal Range (Mean of monthly (max temp - min temp)) BIO3 = Isothermality (BIO2/BIO7) (* 100) BIO5 = Max Temperature of Warmest Month BIO6 = Min Temperature of Coldest Month BIO10 = Mean Temperature of Warmest Quarter BIO12 = Annual Precipitation BIO13 = Precipitation of Wettest Month BIO14 = Precipitation of Driest Month BIO15 = Precipitation Seasonality (Coefficient of Variation) BIO16 = Precipitation of Wettest Quarter BIO17 = Precipitation of Driest Quarter | BIO4, BIO7, BIO11 |
|  | BIO1 = Annual Mean Temperature BIO2 = Mean Diurnal Range (Mean of monthly (max temp - min temp)) BIO3 = Isothermality (BIO2/BIO7) (* 100) BIO5 = Max Temperature of Warmest Month BIO12 = Annual Precipitation BIO13 = Precipitation of Wettest Month BIO14 = Precipitation of Driest Month BIO15 = Precipitation Seasonality (Coefficient of Variation) BIO16 = Precipitation of Wettest Quarter | BIO6, BIO17, BIO10 |
|  | BIO1 = Annual Mean Temperature BIO2 = Mean Diurnal Range (Mean of monthly (max temp - min temp)) (BIO2/BIO7) (* 100) BIO5 = Max Temperature of Warmest Month BIO14 = Precipitation of Driest Month BIO15 = Precipitation Seasonality (Coefficient of Variation) BIO16 = Precipitation of Wettest Quarter | BIO13, BIO12, BIO3 |

**Jackknife Plots:**

*Step 1:*


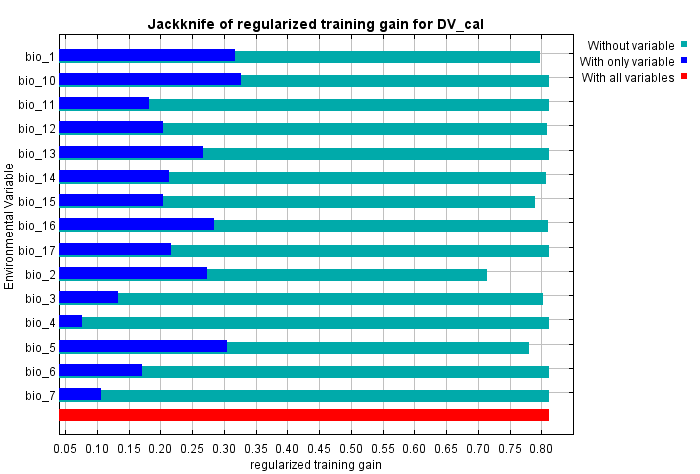


*Step 2*


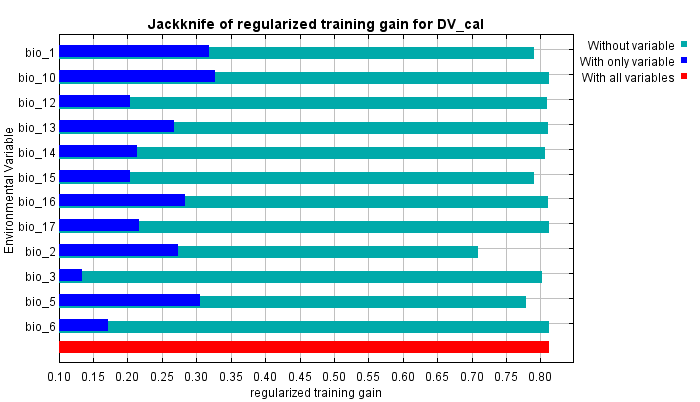


*Step 3*


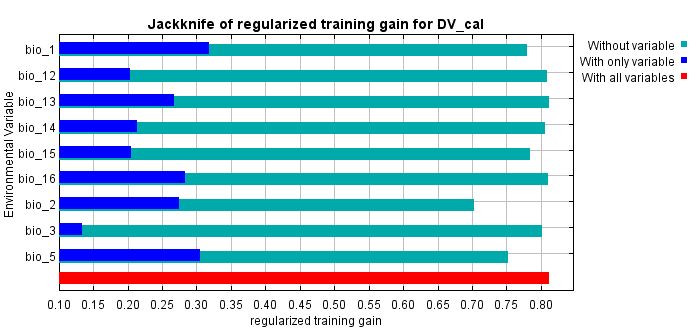


*Step 4*


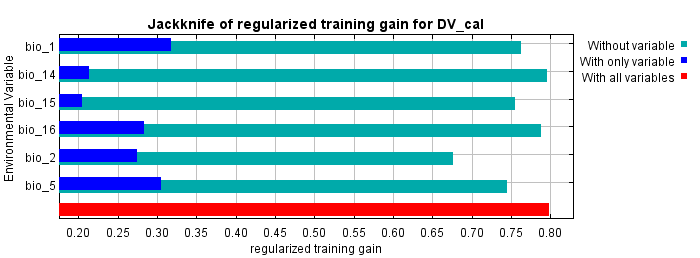


**Table 2. Variable sets for model evaluation:**

| Variable set | Variables |
| --- | --- |
| Set 1 | BIO1 = Annual Mean Temperature BIO2 = Mean Diurnal Range (Mean of monthly (max temp - min temp)) (BIO2/BIO7) (* 100) BIO5 = Max Temperature of Warmest Month BIO14 = Precipitation of Driest Month BIO15 = Precipitation Seasonality (Coefficient of Variation) BIO16 = Precipitation of Wettest Quarter |
| Set 2 | BIO1 = Annual Mean Temperature BIO2 = Mean Diurnal Range (Mean of monthly (max temp - min temp)) BIO3 = Isothermality (BIO2/BIO7) (* 100) BIO5 = Max Temperature of Warmest Month BIO12 = Annual Precipitation BIO13 = Precipitation of Wettest Month BIO14 = Precipitation of Driest Month BIO15 = Precipitation Seasonality (Coefficient of Variation) BIO16 = Precipitation of Wettest Quarter |
| Set 3 | BIO1 = Annual Mean Temperature BIO2 = Mean Diurnal Range (Mean of monthly (max temp - min temp)) BIO3 = Isothermality (BIO2/BIO7) (* 100) BIO5 = Max Temperature of Warmest Month BIO6 = Min Temperature of Coldest Month BIO10 = Mean Temperature of Warmest Quarter BIO12 = Annual Precipitation BIO13 = Precipitation of Wettest Month BIO14 = Precipitation of Driest Month BIO15 = Precipitation Seasonality (Coefficient of Variation) BIO16 = Precipitation of Wettest Quarter BIO17 = Precipitation of Driest Quarter |
