# Supplementary material for: Assessing the current and future potential geographic distribution of the American dog tick, Dermacentor variabilis (Say) (Acari: Ixodidae) in North America
Source: PLoS One. 2020 Aug 10;15(8):e0237191. doi: 10.1371/journal.pone.0237191 (PMC7416948; doi:10.1371/journal.pone.0237191)
Supplement: S3 File — (ZIP) [file pone.0237191.s003.zip › SF3/evaluation_results.html]

ku.enm: evaluation results


# ku.enm: evaluation results

- Breif description of the models evaluation and selection process
- Models evaluation statistics
- Best models according to pre-defined criteria
- Models preformance plot
- Performance evaluation statistics for all models

### Breif description of the models evaluation and selection process

This is the final report of the ku.enm.eval function implemented in the ku.enm R package.

A total of 1479 calibration models with parameters resulted from the combination of 17 regularization multipliers, 29 feature classes, and 3 distinct sets of environmental variables, have been evaluated. Models peformance was evaluated based on statistical significance (Partial\_ROC), omission rates, and the Akaike information criterion corrected for small sample sizes (AICc).

Table 1. Parameters of the evaluated models.

|  | Parameters |
| --- | --- |
| Regularization multipliers | 0.1, 0.2, 0.3, 0.4, 0.5, 0.6, 0.7, 0.8, 0.9, 1, 2, 3, 4, 5, 6, 8, 10 |
| Feature classes | l, q, p, t, h, lq, lp, lt, lh, qp, qt, qh, pt, ph, th, lqp, lqt, lqh, lpt, lph, qpt, qph, qth, pth, lqpt, lqph, lqth, lpth, lqpth |
| Sets of predictors | Set1, Set2, Set3 |

All the results presented below can be found in the evaluation output folder for further analyses.

### Models evaluation statistics

In the following table you are going to find information about how many models meet the four criteria of selection that this function uses.

Table 2. General statistics of models that meet distinct criteria.

| Criteria | Number\_of\_models |
| --- | --- |
| Statistically significant models | 1479 |
| Models meeting OR criteria | 47 |
| Models meeting AICc critera | 2 |
| Models meeting OR and AICc criteria | 1 |

### Best models according to pre-defined criteria

The following table contains the best models selected by your pre-defined criteria.

Notice that if the selection criterion was, models with the best Omission rates and among them those with lower AICc values, the delta\_AICc values were recalculated among the new candidate models.

Table 3. Performance statistics of the best models selected based on the pre-defined critera.

| Model | Mean\_AUC\_ratio | Partial\_ROC | Ommission\_rate\_5% | AICc | delta\_AICc | W\_AICc | num\_parameters |
| --- | --- | --- | --- | --- | --- | --- | --- |
| M\_4\_F\_qth\_Set3 | 1.674 | 0 | 0.036 | 2173.567 | 0 | 0.997 | 18 |

### Models preformance plot

The figure below shows the position of your selected models in the distribution of all the calibration models according to omission rates and AICc values.

Figure 1. Distribution of all, non-statistically significant, and selected models according to their omission rates and AICc values.

### Performance evaluation statistics for all models

Following you will find the performance statistics for all the calibration models.

Table 4. Performance statistics of all the calibration models.

| Model | Mean\_AUC\_ratio | Partial\_ROC | Ommission\_rate\_5% | AICc | delta\_AICc | W\_AICc | num\_parameters |
| --- | --- | --- | --- | --- | --- | --- | --- |
| M\_0.1\_F\_l\_Set1 | 1.511 | 0 | 0.0360000000000000 | 2272.210000000000 | 123.688000000000002 | 0.000 | 6 |
| M\_0.1\_F\_l\_Set2 | 1.549 | 0 | 0.0180000000000000 | 2270.985000000000 | 122.462999999999994 | 0.000 | 9 |
| M\_0.1\_F\_l\_Set3 | 1.544 | 0 | 0.0550000000000000 | 2275.280000000000 | 126.757000000000005 | 0.000 | 12 |
| M\_0.1\_F\_q\_Set1 | 1.552 | 0 | 0.0730000000000000 | 2236.523000000000 | 88.001000000000005 | 0.000 | 6 |
| M\_0.1\_F\_q\_Set2 | 1.606 | 0 | 0.0180000000000000 | 2201.175000000000 | 52.652000000000001 | 0.000 | 9 |
| M\_0.1\_F\_q\_Set3 | 1.640 | 0 | 0.0910000000000000 | 2192.001000000000 | 43.478000000000002 | 0.000 | 12 |
| M\_0.1\_F\_p\_Set1 | 1.589 | 0 | 0.1090000000000000 | 2229.252000000000 | 80.730000000000004 | 0.000 | 15 |
| M\_0.1\_F\_p\_Set2 | 1.622 | 0 | 0.1090000000000000 | 2238.071000000000 | 89.548000000000002 | 0.000 | 27 |
| M\_0.1\_F\_p\_Set3 | 1.647 | 0 | 0.0910000000000000 | 2295.033000000000 | 146.509999999999991 | 0.000 | 45 |
| M\_0.1\_F\_t\_Set1 | 1.663 | 0 | 0.6180000000000000 | NA | NA | NA | 156 |
| M\_0.1\_F\_t\_Set2 | 1.673 | 0 | 0.8000000000000000 | NA | NA | NA | 186 |
| M\_0.1\_F\_t\_Set3 | 1.653 | 0 | 0.9090000000000000 | NA | NA | NA | 211 |
| M\_0.1\_F\_h\_Set1 | 1.688 | 0 | 0.2180000000000000 | 5753.442000000000 | 3604.920000000000073 | 0.000 | 102 |
| M\_0.1\_F\_h\_Set2 | 1.658 | 0 | 0.2550000000000000 | NA | NA | NA | 134 |
| M\_0.1\_F\_h\_Set3 | 1.649 | 0 | 0.3270000000000000 | NA | NA | NA | 166 |
| M\_0.1\_F\_lq\_Set1 | 1.657 | 0 | 0.0910000000000000 | 2165.969000000000 | 17.446000000000002 | 0.000 | 12 |
| M\_0.1\_F\_lq\_Set2 | 1.671 | 0 | 0.0910000000000000 | 2169.838000000000 | 21.315999999999999 | 0.000 | 16 |
| M\_0.1\_F\_lq\_Set3 | 1.677 | 0 | 0.0730000000000000 | 2186.054000000000 | 37.530999999999999 | 0.000 | 22 |
| M\_0.1\_F\_lp\_Set1 | 1.657 | 0 | 0.0910000000000000 | 2194.361000000000 | 45.838000000000001 | 0.000 | 19 |
| M\_0.1\_F\_lp\_Set2 | 1.659 | 0 | 0.0910000000000000 | 2239.024000000000 | 90.501999999999995 | 0.000 | 33 |
| M\_0.1\_F\_lp\_Set3 | 1.652 | 0 | 0.0910000000000000 | 2343.203000000000 | 194.681000000000012 | 0.000 | 54 |
| M\_0.1\_F\_lt\_Set1 | 1.668 | 0 | 0.6180000000000000 | NA | NA | NA | 156 |
| M\_0.1\_F\_lt\_Set2 | 1.674 | 0 | 0.8000000000000000 | NA | NA | NA | 186 |
| M\_0.1\_F\_lt\_Set3 | 1.648 | 0 | 0.9090000000000000 | NA | NA | NA | 211 |
| M\_0.1\_F\_lh\_Set1 | 1.678 | 0 | 0.2360000000000000 | 4774.364000000000 | 2625.842000000000098 | 0.000 | 100 |
| M\_0.1\_F\_lh\_Set2 | 1.654 | 0 | 0.2550000000000000 | NA | NA | NA | 134 |
| M\_0.1\_F\_lh\_Set3 | 1.649 | 0 | 0.3270000000000000 | NA | NA | NA | 156 |
| M\_0.1\_F\_qp\_Set1 | 1.642 | 0 | 0.0910000000000000 | 2202.747000000000 | 54.225000000000001 | 0.000 | 20 |
| M\_0.1\_F\_qp\_Set2 | 1.651 | 0 | 0.0910000000000000 | 2257.912000000000 | 109.388999999999996 | 0.000 | 37 |
| M\_0.1\_F\_qp\_Set3 | 1.661 | 0 | 0.0910000000000000 | 2335.477000000000 | 186.954000000000008 | 0.000 | 52 |
| M\_0.1\_F\_qt\_Set1 | 1.661 | 0 | 0.6180000000000000 | NA | NA | NA | 156 |
| M\_0.1\_F\_qt\_Set2 | 1.675 | 0 | 0.8000000000000000 | NA | NA | NA | 186 |
| M\_0.1\_F\_qt\_Set3 | 1.654 | 0 | 0.9090000000000000 | NA | NA | NA | 211 |
| M\_0.1\_F\_qh\_Set1 | 1.692 | 0 | 0.2180000000000000 | 3509.833000000000 | 1361.309999999999945 | 0.000 | 94 |
| M\_0.1\_F\_qh\_Set2 | 1.653 | 0 | 0.2550000000000000 | NA | NA | NA | 136 |
| M\_0.1\_F\_qh\_Set3 | 1.654 | 0 | 0.3270000000000000 | NA | NA | NA | 157 |
| M\_0.1\_F\_pt\_Set1 | 1.664 | 0 | 0.6180000000000000 | NA | NA | NA | 156 |
| M\_0.1\_F\_pt\_Set2 | 1.681 | 0 | 0.8000000000000000 | NA | NA | NA | 186 |
| M\_0.1\_F\_pt\_Set3 | 1.654 | 0 | 0.9090000000000000 | NA | NA | NA | 211 |
| M\_0.1\_F\_ph\_Set1 | 1.684 | 0 | 0.2360000000000000 | 4772.476000000000 | 2623.952999999999975 | 0.000 | 100 |
| M\_0.1\_F\_ph\_Set2 | 1.659 | 0 | 0.2550000000000000 | NA | NA | NA | 133 |
| M\_0.1\_F\_ph\_Set3 | 1.646 | 0 | 0.3090000000000000 | NA | NA | NA | 175 |
| M\_0.1\_F\_th\_Set1 | 1.662 | 0 | 0.5820000000000000 | NA | NA | NA | 160 |
| M\_0.1\_F\_th\_Set2 | 1.682 | 0 | 0.7450000000000000 | NA | NA | NA | 204 |
| M\_0.1\_F\_th\_Set3 | 1.660 | 0 | 0.9270000000000000 | NA | NA | NA | 216 |
| M\_0.1\_F\_lqp\_Set1 | 1.664 | 0 | 0.0910000000000000 | 2192.295000000000 | 43.773000000000003 | 0.000 | 23 |
| M\_0.1\_F\_lqp\_Set2 | 1.665 | 0 | 0.1090000000000000 | 2249.337000000000 | 100.813999999999993 | 0.000 | 39 |
| M\_0.1\_F\_lqp\_Set3 | 1.666 | 0 | 0.0910000000000000 | 2390.340000000000 | 241.818000000000012 | 0.000 | 59 |
| M\_0.1\_F\_lqt\_Set1 | 1.667 | 0 | 0.6180000000000000 | NA | NA | NA | 156 |
| M\_0.1\_F\_lqt\_Set2 | 1.677 | 0 | 0.8000000000000000 | NA | NA | NA | 186 |
| M\_0.1\_F\_lqt\_Set3 | 1.653 | 0 | 0.9090000000000000 | NA | NA | NA | 211 |
| M\_0.1\_F\_lqh\_Set1 | 1.683 | 0 | 0.2360000000000000 | 3790.501000000000 | 1641.979000000000042 | 0.000 | 96 |
| M\_0.1\_F\_lqh\_Set2 | 1.658 | 0 | 0.2550000000000000 | NA | NA | NA | 136 |
| M\_0.1\_F\_lqh\_Set3 | 1.651 | 0 | 0.3270000000000000 | NA | NA | NA | 160 |
| M\_0.1\_F\_lpt\_Set1 | 1.667 | 0 | 0.6180000000000000 | NA | NA | NA | 156 |
| M\_0.1\_F\_lpt\_Set2 | 1.677 | 0 | 0.8000000000000000 | NA | NA | NA | 186 |
| M\_0.1\_F\_lpt\_Set3 | 1.650 | 0 | 0.9090000000000000 | NA | NA | NA | 211 |
| M\_0.1\_F\_lph\_Set1 | 1.683 | 0 | 0.2360000000000000 | 5752.491000000000 | 3603.969000000000051 | 0.000 | 102 |
| M\_0.1\_F\_lph\_Set2 | 1.664 | 0 | 0.2550000000000000 | NA | NA | NA | 133 |
| M\_0.1\_F\_lph\_Set3 | 1.639 | 0 | 0.3820000000000000 | NA | NA | NA | 175 |
| M\_0.1\_F\_qpt\_Set1 | 1.667 | 0 | 0.6180000000000000 | NA | NA | NA | 156 |
| M\_0.1\_F\_qpt\_Set2 | 1.676 | 0 | 0.8000000000000000 | NA | NA | NA | 186 |
| M\_0.1\_F\_qpt\_Set3 | 1.651 | 0 | 0.9090000000000000 | NA | NA | NA | 211 |
| M\_0.1\_F\_qph\_Set1 | 1.680 | 0 | 0.2360000000000000 | NA | NA | NA | 108 |
| M\_0.1\_F\_qph\_Set2 | 1.657 | 0 | 0.2550000000000000 | NA | NA | NA | 142 |
| M\_0.1\_F\_qph\_Set3 | 1.650 | 0 | 0.3090000000000000 | NA | NA | NA | 172 |
| M\_0.1\_F\_qth\_Set1 | 1.662 | 0 | 0.5820000000000000 | NA | NA | NA | 160 |
| M\_0.1\_F\_qth\_Set2 | 1.681 | 0 | 0.7450000000000000 | NA | NA | NA | 204 |
| M\_0.1\_F\_qth\_Set3 | 1.664 | 0 | 0.9270000000000000 | NA | NA | NA | 216 |
| M\_0.1\_F\_pth\_Set1 | 1.664 | 0 | 0.5820000000000000 | NA | NA | NA | 160 |
| M\_0.1\_F\_pth\_Set2 | 1.677 | 0 | 0.7450000000000000 | NA | NA | NA | 204 |
| M\_0.1\_F\_pth\_Set3 | 1.659 | 0 | 0.9270000000000000 | NA | NA | NA | 216 |
| M\_0.1\_F\_lqpt\_Set1 | 1.664 | 0 | 0.6180000000000000 | NA | NA | NA | 156 |
| M\_0.1\_F\_lqpt\_Set2 | 1.677 | 0 | 0.8000000000000000 | NA | NA | NA | 186 |
| M\_0.1\_F\_lqpt\_Set3 | 1.649 | 0 | 0.9090000000000000 | NA | NA | NA | 211 |
| M\_0.1\_F\_lqph\_Set1 | 1.685 | 0 | 0.2360000000000000 | 7714.105000000000 | 5565.582999999999629 | 0.000 | 104 |
| M\_0.1\_F\_lqph\_Set2 | 1.670 | 0 | 0.2730000000000000 | NA | NA | NA | 154 |
| M\_0.1\_F\_lqph\_Set3 | 1.653 | 0 | 0.3090000000000000 | NA | NA | NA | 173 |
| M\_0.1\_F\_lqth\_Set1 | 1.666 | 0 | 0.5820000000000000 | NA | NA | NA | 160 |
| M\_0.1\_F\_lqth\_Set2 | 1.678 | 0 | 0.7450000000000000 | NA | NA | NA | 204 |
| M\_0.1\_F\_lqth\_Set3 | 1.660 | 0 | 0.9270000000000000 | NA | NA | NA | 216 |
| M\_0.1\_F\_lpth\_Set1 | 1.662 | 0 | 0.5820000000000000 | NA | NA | NA | 160 |
| M\_0.1\_F\_lpth\_Set2 | 1.680 | 0 | 0.7450000000000000 | NA | NA | NA | 204 |
| M\_0.1\_F\_lpth\_Set3 | 1.666 | 0 | 0.9270000000000000 | NA | NA | NA | 216 |
| M\_0.1\_F\_lqpth\_Set1 | 1.670 | 0 | 0.5820000000000000 | NA | NA | NA | 160 |
| M\_0.1\_F\_lqpth\_Set2 | 1.679 | 0 | 0.7450000000000000 | NA | NA | NA | 204 |
| M\_0.1\_F\_lqpth\_Set3 | 1.665 | 0 | 0.9270000000000000 | NA | NA | NA | 216 |
| M\_0.2\_F\_l\_Set1 | 1.514 | 0 | 0.0360000000000000 | 2272.189000000000 | 123.667000000000002 | 0.000 | 6 |
| M\_0.2\_F\_l\_Set2 | 1.552 | 0 | 0.0180000000000000 | 2271.157000000000 | 122.634000000000000 | 0.000 | 9 |
| M\_0.2\_F\_l\_Set3 | 1.551 | 0 | 0.0360000000000000 | 2273.553000000000 | 125.031000000000006 | 0.000 | 12 |
| M\_0.2\_F\_q\_Set1 | 1.555 | 0 | 0.0730000000000000 | 2236.546000000000 | 88.022999999999996 | 0.000 | 6 |
| M\_0.2\_F\_q\_Set2 | 1.611 | 0 | 0.0180000000000000 | 2201.635000000000 | 53.113000000000000 | 0.000 | 9 |
| M\_0.2\_F\_q\_Set3 | 1.637 | 0 | 0.0910000000000000 | 2189.816000000000 | 41.293999999999997 | 0.000 | 11 |
| M\_0.2\_F\_p\_Set1 | 1.591 | 0 | 0.0910000000000000 | 2229.240000000000 | 80.718000000000004 | 0.000 | 15 |
| M\_0.2\_F\_p\_Set2 | 1.608 | 0 | 0.0550000000000000 | 2237.875000000000 | 89.352999999999994 | 0.000 | 27 |
| M\_0.2\_F\_p\_Set3 | 1.646 | 0 | 0.0730000000000000 | 2326.484000000000 | 177.961999999999989 | 0.000 | 50 |
| M\_0.2\_F\_t\_Set1 | 1.692 | 0 | 0.5270000000000000 | NA | NA | NA | 136 |
| M\_0.2\_F\_t\_Set2 | 1.673 | 0 | 0.7090000000000000 | NA | NA | NA | 165 |
| M\_0.2\_F\_t\_Set3 | 1.665 | 0 | 0.6909999999999999 | NA | NA | NA | 184 |
| M\_0.2\_F\_h\_Set1 | 1.703 | 0 | 0.1090000000000000 | 2867.398000000000 | 718.875999999999976 | 0.000 | 85 |
| M\_0.2\_F\_h\_Set2 | 1.682 | 0 | 0.1640000000000000 | 5186.492000000000 | 3037.969999999999800 | 0.000 | 101 |
| M\_0.2\_F\_h\_Set3 | 1.688 | 0 | 0.1820000000000000 | NA | NA | NA | 119 |
| M\_0.2\_F\_lq\_Set1 | 1.664 | 0 | 0.0910000000000000 | 2164.692000000000 | 16.170000000000002 | 0.000 | 12 |
| M\_0.2\_F\_lq\_Set2 | 1.666 | 0 | 0.0910000000000000 | 2170.170000000000 | 21.648000000000000 | 0.000 | 16 |
| M\_0.2\_F\_lq\_Set3 | 1.675 | 0 | 0.0730000000000000 | 2180.378000000000 | 31.855000000000000 | 0.000 | 20 |
| M\_0.2\_F\_lp\_Set1 | 1.658 | 0 | 0.0910000000000000 | 2194.394000000000 | 45.871000000000002 | 0.000 | 18 |
| M\_0.2\_F\_lp\_Set2 | 1.661 | 0 | 0.0910000000000000 | 2237.341000000000 | 88.819000000000003 | 0.000 | 32 |
| M\_0.2\_F\_lp\_Set3 | 1.662 | 0 | 0.0730000000000000 | 2352.990000000000 | 204.467000000000013 | 0.000 | 54 |
| M\_0.2\_F\_lt\_Set1 | 1.691 | 0 | 0.5270000000000000 | NA | NA | NA | 136 |
| M\_0.2\_F\_lt\_Set2 | 1.678 | 0 | 0.7090000000000000 | NA | NA | NA | 165 |
| M\_0.2\_F\_lt\_Set3 | 1.663 | 0 | 0.6909999999999999 | NA | NA | NA | 184 |
| M\_0.2\_F\_lh\_Set1 | 1.693 | 0 | 0.1090000000000000 | 2715.110000000000 | 566.587999999999965 | 0.000 | 81 |
| M\_0.2\_F\_lh\_Set2 | 1.683 | 0 | 0.1640000000000000 | 4767.780000000000 | 2619.257999999999811 | 0.000 | 100 |
| M\_0.2\_F\_lh\_Set3 | 1.685 | 0 | 0.1820000000000000 | NA | NA | NA | 110 |
| M\_0.2\_F\_qp\_Set1 | 1.637 | 0 | 0.0910000000000000 | 2203.143000000000 | 54.619999999999997 | 0.000 | 20 |
| M\_0.2\_F\_qp\_Set2 | 1.652 | 0 | 0.0730000000000000 | 2236.414000000000 | 87.891000000000005 | 0.000 | 33 |
| M\_0.2\_F\_qp\_Set3 | 1.653 | 0 | 0.0730000000000000 | 2363.810000000000 | 215.287000000000006 | 0.000 | 55 |
| M\_0.2\_F\_qt\_Set1 | 1.687 | 0 | 0.5270000000000000 | NA | NA | NA | 136 |
| M\_0.2\_F\_qt\_Set2 | 1.682 | 0 | 0.7090000000000000 | NA | NA | NA | 165 |
| M\_0.2\_F\_qt\_Set3 | 1.662 | 0 | 0.6909999999999999 | NA | NA | NA | 184 |
| M\_0.2\_F\_qh\_Set1 | 1.704 | 0 | 0.0730000000000000 | 2784.923000000000 | 636.400999999999954 | 0.000 | 83 |
| M\_0.2\_F\_qh\_Set2 | 1.684 | 0 | 0.1640000000000000 | 5185.214000000000 | 3036.692000000000007 | 0.000 | 101 |
| M\_0.2\_F\_qh\_Set3 | 1.690 | 0 | 0.1820000000000000 | NA | NA | NA | 120 |
| M\_0.2\_F\_pt\_Set1 | 1.685 | 0 | 0.5270000000000000 | NA | NA | NA | 136 |
| M\_0.2\_F\_pt\_Set2 | 1.674 | 0 | 0.7090000000000000 | NA | NA | NA | 165 |
| M\_0.2\_F\_pt\_Set3 | 1.668 | 0 | 0.6909999999999999 | NA | NA | NA | 184 |
| M\_0.2\_F\_ph\_Set1 | 1.702 | 0 | 0.1090000000000000 | 2681.869000000000 | 533.346999999999980 | 0.000 | 80 |
| M\_0.2\_F\_ph\_Set2 | 1.679 | 0 | 0.1450000000000000 | 3782.887000000000 | 1634.365000000000009 | 0.000 | 96 |
| M\_0.2\_F\_ph\_Set3 | 1.687 | 0 | 0.1820000000000000 | NA | NA | NA | 115 |
| M\_0.2\_F\_th\_Set1 | 1.679 | 0 | 0.5450000000000000 | NA | NA | NA | 143 |
| M\_0.2\_F\_th\_Set2 | 1.676 | 0 | 0.6730000000000000 | NA | NA | NA | 180 |
| M\_0.2\_F\_th\_Set3 | 1.663 | 0 | 0.7270000000000000 | NA | NA | NA | 187 |
| M\_0.2\_F\_lqp\_Set1 | 1.661 | 0 | 0.0910000000000000 | 2202.083000000000 | 53.560000000000002 | 0.000 | 25 |
| M\_0.2\_F\_lqp\_Set2 | 1.655 | 0 | 0.0910000000000000 | 2243.109000000000 | 94.587000000000003 | 0.000 | 37 |
| M\_0.2\_F\_lqp\_Set3 | 1.660 | 0 | 0.0730000000000000 | 2346.694000000000 | 198.171999999999997 | 0.000 | 54 |
| M\_0.2\_F\_lqt\_Set1 | 1.697 | 0 | 0.5270000000000000 | NA | NA | NA | 136 |
| M\_0.2\_F\_lqt\_Set2 | 1.676 | 0 | 0.7090000000000000 | NA | NA | NA | 165 |
| M\_0.2\_F\_lqt\_Set3 | 1.666 | 0 | 0.6909999999999999 | NA | NA | NA | 184 |
| M\_0.2\_F\_lqh\_Set1 | 1.697 | 0 | 0.0730000000000000 | 2785.440000000000 | 636.917000000000030 | 0.000 | 83 |
| M\_0.2\_F\_lqh\_Set2 | 1.682 | 0 | 0.1640000000000000 | 4766.289000000000 | 2617.766000000000076 | 0.000 | 100 |
| M\_0.2\_F\_lqh\_Set3 | 1.687 | 0 | 0.1820000000000000 | NA | NA | NA | 115 |
| M\_0.2\_F\_lpt\_Set1 | 1.679 | 0 | 0.5270000000000000 | NA | NA | NA | 136 |
| M\_0.2\_F\_lpt\_Set2 | 1.678 | 0 | 0.7090000000000000 | NA | NA | NA | 165 |
| M\_0.2\_F\_lpt\_Set3 | 1.664 | 0 | 0.6909999999999999 | NA | NA | NA | 184 |
| M\_0.2\_F\_lph\_Set1 | 1.698 | 0 | 0.1090000000000000 | 2681.945000000000 | 533.423000000000002 | 0.000 | 80 |
| M\_0.2\_F\_lph\_Set2 | 1.681 | 0 | 0.1450000000000000 | 9669.486000000001 | 7520.963999999999942 | 0.000 | 105 |
| M\_0.2\_F\_lph\_Set3 | 1.685 | 0 | 0.1820000000000000 | NA | NA | NA | 132 |
| M\_0.2\_F\_qpt\_Set1 | 1.688 | 0 | 0.5270000000000000 | NA | NA | NA | 136 |
| M\_0.2\_F\_qpt\_Set2 | 1.680 | 0 | 0.7090000000000000 | NA | NA | NA | 165 |
| M\_0.2\_F\_qpt\_Set3 | 1.663 | 0 | 0.6909999999999999 | NA | NA | NA | 184 |
| M\_0.2\_F\_qph\_Set1 | 1.707 | 0 | 0.0910000000000000 | 2782.192000000000 | 633.669999999999959 | 0.000 | 83 |
| M\_0.2\_F\_qph\_Set2 | 1.684 | 0 | 0.1450000000000000 | 9669.809999999999 | 7521.287000000000262 | 0.000 | 105 |
| M\_0.2\_F\_qph\_Set3 | 1.679 | 0 | 0.1820000000000000 | NA | NA | NA | 123 |
| M\_0.2\_F\_qth\_Set1 | 1.683 | 0 | 0.5450000000000000 | NA | NA | NA | 143 |
| M\_0.2\_F\_qth\_Set2 | 1.679 | 0 | 0.6730000000000000 | NA | NA | NA | 180 |
| M\_0.2\_F\_qth\_Set3 | 1.662 | 0 | 0.7270000000000000 | NA | NA | NA | 187 |
| M\_0.2\_F\_pth\_Set1 | 1.682 | 0 | 0.5450000000000000 | NA | NA | NA | 143 |
| M\_0.2\_F\_pth\_Set2 | 1.679 | 0 | 0.6730000000000000 | NA | NA | NA | 180 |
| M\_0.2\_F\_pth\_Set3 | 1.666 | 0 | 0.7270000000000000 | NA | NA | NA | 187 |
| M\_0.2\_F\_lqpt\_Set1 | 1.681 | 0 | 0.5270000000000000 | NA | NA | NA | 136 |
| M\_0.2\_F\_lqpt\_Set2 | 1.676 | 0 | 0.7090000000000000 | NA | NA | NA | 165 |
| M\_0.2\_F\_lqpt\_Set3 | 1.669 | 0 | 0.6909999999999999 | NA | NA | NA | 184 |
| M\_0.2\_F\_lqph\_Set1 | 1.705 | 0 | 0.0910000000000000 | 2713.894000000000 | 565.371999999999957 | 0.000 | 81 |
| M\_0.2\_F\_lqph\_Set2 | 1.676 | 0 | 0.1450000000000000 | 6530.777000000000 | 4382.255000000000109 | 0.000 | 103 |
| M\_0.2\_F\_lqph\_Set3 | 1.688 | 0 | 0.1820000000000000 | NA | NA | NA | 134 |
| M\_0.2\_F\_lqth\_Set1 | 1.681 | 0 | 0.5450000000000000 | NA | NA | NA | 143 |
| M\_0.2\_F\_lqth\_Set2 | 1.677 | 0 | 0.6730000000000000 | NA | NA | NA | 180 |
| M\_0.2\_F\_lqth\_Set3 | 1.666 | 0 | 0.7270000000000000 | NA | NA | NA | 187 |
| M\_0.2\_F\_lpth\_Set1 | 1.676 | 0 | 0.5450000000000000 | NA | NA | NA | 143 |
| M\_0.2\_F\_lpth\_Set2 | 1.676 | 0 | 0.6730000000000000 | NA | NA | NA | 180 |
| M\_0.2\_F\_lpth\_Set3 | 1.670 | 0 | 0.7270000000000000 | NA | NA | NA | 187 |
| M\_0.2\_F\_lqpth\_Set1 | 1.685 | 0 | 0.5450000000000000 | NA | NA | NA | 143 |
| M\_0.2\_F\_lqpth\_Set2 | 1.678 | 0 | 0.6730000000000000 | NA | NA | NA | 180 |
| M\_0.2\_F\_lqpth\_Set3 | 1.667 | 0 | 0.7270000000000000 | NA | NA | NA | 187 |
| M\_0.3\_F\_l\_Set1 | 1.512 | 0 | 0.0360000000000000 | 2272.189000000000 | 123.665999999999997 | 0.000 | 6 |
| M\_0.3\_F\_l\_Set2 | 1.544 | 0 | 0.0180000000000000 | 2271.359000000000 | 122.837000000000003 | 0.000 | 9 |
| M\_0.3\_F\_l\_Set3 | 1.547 | 0 | 0.0180000000000000 | 2274.814000000000 | 126.292000000000002 | 0.000 | 12 |
| M\_0.3\_F\_q\_Set1 | 1.550 | 0 | 0.0730000000000000 | 2236.577000000000 | 88.055000000000007 | 0.000 | 6 |
| M\_0.3\_F\_q\_Set2 | 1.604 | 0 | 0.0180000000000000 | 2202.411000000000 | 53.889000000000003 | 0.000 | 9 |
| M\_0.3\_F\_q\_Set3 | 1.635 | 0 | 0.0910000000000000 | 2192.971000000000 | 44.448000000000000 | 0.000 | 12 |
| M\_0.3\_F\_p\_Set1 | 1.582 | 0 | 0.0730000000000000 | 2224.244000000000 | 75.721999999999994 | 0.000 | 13 |
| M\_0.3\_F\_p\_Set2 | 1.594 | 0 | 0.0730000000000000 | 2227.285000000000 | 78.762000000000000 | 0.000 | 23 |
| M\_0.3\_F\_p\_Set3 | 1.643 | 0 | 0.0730000000000000 | 2313.764000000000 | 165.241999999999990 | 0.000 | 47 |
| M\_0.3\_F\_t\_Set1 | 1.702 | 0 | 0.2360000000000000 | NA | NA | NA | 121 |
| M\_0.3\_F\_t\_Set2 | 1.695 | 0 | 0.4360000000000000 | NA | NA | NA | 147 |
| M\_0.3\_F\_t\_Set3 | 1.693 | 0 | 0.5270000000000000 | NA | NA | NA | 172 |
| M\_0.3\_F\_h\_Set1 | 1.707 | 0 | 0.0910000000000000 | 2417.134000000000 | 268.612000000000023 | 0.000 | 66 |
| M\_0.3\_F\_h\_Set2 | 1.689 | 0 | 0.1270000000000000 | 2908.476000000000 | 759.953999999999951 | 0.000 | 86 |
| M\_0.3\_F\_h\_Set3 | 1.710 | 0 | 0.1450000000000000 | 2947.993000000000 | 799.471000000000004 | 0.000 | 87 |
| M\_0.3\_F\_lq\_Set1 | 1.669 | 0 | 0.0910000000000000 | 2165.020000000000 | 16.497000000000000 | 0.000 | 12 |
| M\_0.3\_F\_lq\_Set2 | 1.662 | 0 | 0.0910000000000000 | 2172.228000000000 | 23.704999999999998 | 0.000 | 16 |
| M\_0.3\_F\_lq\_Set3 | 1.676 | 0 | 0.0730000000000000 | 2186.877000000000 | 38.354999999999997 | 0.000 | 22 |
| M\_0.3\_F\_lp\_Set1 | 1.649 | 0 | 0.0910000000000000 | 2191.646000000000 | 43.122999999999998 | 0.000 | 17 |
| M\_0.3\_F\_lp\_Set2 | 1.649 | 0 | 0.0910000000000000 | 2226.766000000000 | 78.244000000000000 | 0.000 | 28 |
| M\_0.3\_F\_lp\_Set3 | 1.653 | 0 | 0.0730000000000000 | 2329.421000000000 | 180.899000000000001 | 0.000 | 51 |
| M\_0.3\_F\_lt\_Set1 | 1.709 | 0 | 0.2000000000000000 | NA | NA | NA | 121 |
| M\_0.3\_F\_lt\_Set2 | 1.696 | 0 | 0.4360000000000000 | NA | NA | NA | 147 |
| M\_0.3\_F\_lt\_Set3 | 1.693 | 0 | 0.5270000000000000 | NA | NA | NA | 172 |
| M\_0.3\_F\_lh\_Set1 | 1.701 | 0 | 0.0910000000000000 | 2444.745000000000 | 296.223000000000013 | 0.000 | 68 |
| M\_0.3\_F\_lh\_Set2 | 1.697 | 0 | 0.1270000000000000 | 2650.705000000000 | 502.182999999999993 | 0.000 | 79 |
| M\_0.3\_F\_lh\_Set3 | 1.713 | 0 | 0.1270000000000000 | 3066.461000000000 | 917.938999999999965 | 0.000 | 89 |
| M\_0.3\_F\_qp\_Set1 | 1.642 | 0 | 0.0910000000000000 | 2198.058000000000 | 49.534999999999997 | 0.000 | 18 |
| M\_0.3\_F\_qp\_Set2 | 1.650 | 0 | 0.0730000000000000 | 2225.260000000000 | 76.736999999999995 | 0.000 | 29 |
| M\_0.3\_F\_qp\_Set3 | 1.652 | 0 | 0.0730000000000000 | 2358.928000000000 | 210.406000000000006 | 0.000 | 54 |
| M\_0.3\_F\_qt\_Set1 | 1.708 | 0 | 0.2000000000000000 | NA | NA | NA | 121 |
| M\_0.3\_F\_qt\_Set2 | 1.700 | 0 | 0.4550000000000000 | NA | NA | NA | 147 |
| M\_0.3\_F\_qt\_Set3 | 1.697 | 0 | 0.5270000000000000 | NA | NA | NA | 172 |
| M\_0.3\_F\_qh\_Set1 | 1.707 | 0 | 0.0910000000000000 | 2415.560000000000 | 267.038000000000011 | 0.000 | 66 |
| M\_0.3\_F\_qh\_Set2 | 1.700 | 0 | 0.1270000000000000 | 2780.183000000000 | 631.659999999999968 | 0.000 | 83 |
| M\_0.3\_F\_qh\_Set3 | 1.717 | 0 | 0.1270000000000000 | 3135.650000000000 | 987.126999999999953 | 0.000 | 90 |
| M\_0.3\_F\_pt\_Set1 | 1.706 | 0 | 0.2360000000000000 | NA | NA | NA | 121 |
| M\_0.3\_F\_pt\_Set2 | 1.692 | 0 | 0.4360000000000000 | NA | NA | NA | 147 |
| M\_0.3\_F\_pt\_Set3 | 1.693 | 0 | 0.5270000000000000 | NA | NA | NA | 172 |
| M\_0.3\_F\_ph\_Set1 | 1.705 | 0 | 0.0910000000000000 | 2387.692000000000 | 239.169999999999987 | 0.000 | 64 |
| M\_0.3\_F\_ph\_Set2 | 1.698 | 0 | 0.1270000000000000 | 2956.603000000000 | 808.081000000000017 | 0.000 | 87 |
| M\_0.3\_F\_ph\_Set3 | 1.706 | 0 | 0.1450000000000000 | 5747.299000000000 | 3598.775999999999840 | 0.000 | 102 |
| M\_0.3\_F\_th\_Set1 | 1.714 | 0 | 0.2180000000000000 | NA | NA | NA | 126 |
| M\_0.3\_F\_th\_Set2 | 1.690 | 0 | 0.4910000000000000 | NA | NA | NA | 148 |
| M\_0.3\_F\_th\_Set3 | 1.682 | 0 | 0.5820000000000000 | NA | NA | NA | 175 |
| M\_0.3\_F\_lqp\_Set1 | 1.657 | 0 | 0.0910000000000000 | 2202.502000000000 | 53.979999999999997 | 0.000 | 25 |
| M\_0.3\_F\_lqp\_Set2 | 1.655 | 0 | 0.0730000000000000 | 2231.036000000000 | 82.513000000000005 | 0.000 | 35 |
| M\_0.3\_F\_lqp\_Set3 | 1.659 | 0 | 0.0730000000000000 | 2370.269000000000 | 221.746000000000009 | 0.000 | 57 |
| M\_0.3\_F\_lqt\_Set1 | 1.708 | 0 | 0.2000000000000000 | NA | NA | NA | 121 |
| M\_0.3\_F\_lqt\_Set2 | 1.698 | 0 | 0.4550000000000000 | NA | NA | NA | 147 |
| M\_0.3\_F\_lqt\_Set3 | 1.689 | 0 | 0.5270000000000000 | NA | NA | NA | 172 |
| M\_0.3\_F\_lqh\_Set1 | 1.710 | 0 | 0.0910000000000000 | 2475.127000000000 | 326.603999999999985 | 0.000 | 70 |
| M\_0.3\_F\_lqh\_Set2 | 1.693 | 0 | 0.1270000000000000 | 2744.132000000000 | 595.609000000000037 | 0.000 | 82 |
| M\_0.3\_F\_lqh\_Set3 | 1.707 | 0 | 0.1270000000000000 | 3135.789000000000 | 987.267000000000053 | 0.000 | 90 |
| M\_0.3\_F\_lpt\_Set1 | 1.706 | 0 | 0.2360000000000000 | NA | NA | NA | 121 |
| M\_0.3\_F\_lpt\_Set2 | 1.694 | 0 | 0.4360000000000000 | NA | NA | NA | 147 |
| M\_0.3\_F\_lpt\_Set3 | 1.696 | 0 | 0.5270000000000000 | NA | NA | NA | 172 |
| M\_0.3\_F\_lph\_Set1 | 1.714 | 0 | 0.0910000000000000 | 2412.459000000000 | 263.937000000000012 | 0.000 | 66 |
| M\_0.3\_F\_lph\_Set2 | 1.698 | 0 | 0.1270000000000000 | 2905.266000000000 | 756.744000000000028 | 0.000 | 86 |
| M\_0.3\_F\_lph\_Set3 | 1.712 | 0 | 0.1450000000000000 | NA | NA | NA | 108 |
| M\_0.3\_F\_qpt\_Set1 | 1.705 | 0 | 0.2180000000000000 | NA | NA | NA | 121 |
| M\_0.3\_F\_qpt\_Set2 | 1.699 | 0 | 0.4360000000000000 | NA | NA | NA | 147 |
| M\_0.3\_F\_qpt\_Set3 | 1.698 | 0 | 0.5270000000000000 | NA | NA | NA | 172 |
| M\_0.3\_F\_qph\_Set1 | 1.708 | 0 | 0.0910000000000000 | 2471.308000000000 | 322.786000000000001 | 0.000 | 70 |
| M\_0.3\_F\_qph\_Set2 | 1.692 | 0 | 0.1270000000000000 | 3797.080000000000 | 1648.557999999999993 | 0.000 | 96 |
| M\_0.3\_F\_qph\_Set3 | 1.715 | 0 | 0.1270000000000000 | NA | NA | NA | 112 |
| M\_0.3\_F\_qth\_Set1 | 1.712 | 0 | 0.2180000000000000 | NA | NA | NA | 126 |
| M\_0.3\_F\_qth\_Set2 | 1.693 | 0 | 0.4910000000000000 | NA | NA | NA | 148 |
| M\_0.3\_F\_qth\_Set3 | 1.686 | 0 | 0.5820000000000000 | NA | NA | NA | 175 |
| M\_0.3\_F\_pth\_Set1 | 1.711 | 0 | 0.2180000000000000 | NA | NA | NA | 126 |
| M\_0.3\_F\_pth\_Set2 | 1.691 | 0 | 0.4910000000000000 | NA | NA | NA | 148 |
| M\_0.3\_F\_pth\_Set3 | 1.686 | 0 | 0.5820000000000000 | NA | NA | NA | 175 |
| M\_0.3\_F\_lqpt\_Set1 | 1.708 | 0 | 0.2180000000000000 | NA | NA | NA | 121 |
| M\_0.3\_F\_lqpt\_Set2 | 1.690 | 0 | 0.4360000000000000 | NA | NA | NA | 147 |
| M\_0.3\_F\_lqpt\_Set3 | 1.691 | 0 | 0.5270000000000000 | NA | NA | NA | 172 |
| M\_0.3\_F\_lqph\_Set1 | 1.712 | 0 | 0.0910000000000000 | 2455.513000000000 | 306.990999999999985 | 0.000 | 69 |
| M\_0.3\_F\_lqph\_Set2 | 1.688 | 0 | 0.1270000000000000 | 4189.973000000000 | 2041.451000000000022 | 0.000 | 98 |
| M\_0.3\_F\_lqph\_Set3 | 1.714 | 0 | 0.1450000000000000 | NA | NA | NA | 108 |
| M\_0.3\_F\_lqth\_Set1 | 1.710 | 0 | 0.2180000000000000 | NA | NA | NA | 126 |
| M\_0.3\_F\_lqth\_Set2 | 1.696 | 0 | 0.4910000000000000 | NA | NA | NA | 148 |
| M\_0.3\_F\_lqth\_Set3 | 1.676 | 0 | 0.5820000000000000 | NA | NA | NA | 175 |
| M\_0.3\_F\_lpth\_Set1 | 1.715 | 0 | 0.2180000000000000 | NA | NA | NA | 126 |
| M\_0.3\_F\_lpth\_Set2 | 1.695 | 0 | 0.4910000000000000 | NA | NA | NA | 148 |
| M\_0.3\_F\_lpth\_Set3 | 1.683 | 0 | 0.5820000000000000 | NA | NA | NA | 175 |
| M\_0.3\_F\_lqpth\_Set1 | 1.710 | 0 | 0.2180000000000000 | NA | NA | NA | 126 |
| M\_0.3\_F\_lqpth\_Set2 | 1.697 | 0 | 0.4910000000000000 | NA | NA | NA | 148 |
| M\_0.3\_F\_lqpth\_Set3 | 1.682 | 0 | 0.5820000000000000 | NA | NA | NA | 175 |
| M\_0.4\_F\_l\_Set1 | 1.514 | 0 | 0.0360000000000000 | 2272.196000000000 | 123.673000000000002 | 0.000 | 6 |
| M\_0.4\_F\_l\_Set2 | 1.539 | 0 | 0.0360000000000000 | 2271.770000000000 | 123.247000000000000 | 0.000 | 9 |
| M\_0.4\_F\_l\_Set3 | 1.542 | 0 | 0.0360000000000000 | 2275.252000000000 | 126.730000000000004 | 0.000 | 12 |
| M\_0.4\_F\_q\_Set1 | 1.547 | 0 | 0.0730000000000000 | 2236.617000000000 | 88.093999999999994 | 0.000 | 6 |
| M\_0.4\_F\_q\_Set2 | 1.602 | 0 | 0.0180000000000000 | 2203.730000000000 | 55.207999999999998 | 0.000 | 9 |
| M\_0.4\_F\_q\_Set3 | 1.628 | 0 | 0.0550000000000000 | 2191.591000000000 | 43.067999999999998 | 0.000 | 11 |
| M\_0.4\_F\_p\_Set1 | 1.578 | 0 | 0.0730000000000000 | 2226.725000000000 | 78.203000000000003 | 0.000 | 14 |
| M\_0.4\_F\_p\_Set2 | 1.586 | 0 | 0.0910000000000000 | 2232.050000000000 | 83.527000000000001 | 0.000 | 24 |
| M\_0.4\_F\_p\_Set3 | 1.647 | 0 | 0.0730000000000000 | 2252.353000000000 | 103.829999999999998 | 0.000 | 35 |
| M\_0.4\_F\_t\_Set1 | 1.699 | 0 | 0.1640000000000000 | 4135.577000000000 | 1987.055000000000064 | 0.000 | 98 |
| M\_0.4\_F\_t\_Set2 | 1.710 | 0 | 0.2730000000000000 | NA | NA | NA | 125 |
| M\_0.4\_F\_t\_Set3 | 1.714 | 0 | 0.2910000000000000 | NA | NA | NA | 145 |
| M\_0.4\_F\_h\_Set1 | 1.708 | 0 | 0.1090000000000000 | 2301.656000000000 | 153.133000000000010 | 0.000 | 54 |
| M\_0.4\_F\_h\_Set2 | 1.706 | 0 | 0.1270000000000000 | 2452.249000000000 | 303.726999999999975 | 0.000 | 69 |
| M\_0.4\_F\_h\_Set3 | 1.720 | 0 | 0.1270000000000000 | 2443.078000000000 | 294.555000000000007 | 0.000 | 69 |
| M\_0.4\_F\_lq\_Set1 | 1.668 | 0 | 0.0730000000000000 | 2165.987000000000 | 17.465000000000000 | 0.000 | 12 |
| M\_0.4\_F\_lq\_Set2 | 1.665 | 0 | 0.0730000000000000 | 2169.871000000000 | 21.347999999999999 | 0.000 | 15 |
| M\_0.4\_F\_lq\_Set3 | 1.675 | 0 | 0.0730000000000000 | 2185.966000000000 | 37.442999999999998 | 0.000 | 21 |
| M\_0.4\_F\_lp\_Set1 | 1.637 | 0 | 0.1090000000000000 | 2190.437000000000 | 41.914000000000001 | 0.000 | 16 |
| M\_0.4\_F\_lp\_Set2 | 1.646 | 0 | 0.0910000000000000 | 2223.981000000000 | 75.459000000000003 | 0.000 | 30 |
| M\_0.4\_F\_lp\_Set3 | 1.655 | 0 | 0.0730000000000000 | 2304.160000000000 | 155.638000000000005 | 0.000 | 47 |
| M\_0.4\_F\_lt\_Set1 | 1.704 | 0 | 0.1640000000000000 | 4723.477000000000 | 2574.954000000000178 | 0.000 | 100 |
| M\_0.4\_F\_lt\_Set2 | 1.713 | 0 | 0.2550000000000000 | NA | NA | NA | 125 |
| M\_0.4\_F\_lt\_Set3 | 1.720 | 0 | 0.3090000000000000 | NA | NA | NA | 145 |
| M\_0.4\_F\_lh\_Set1 | 1.710 | 0 | 0.1090000000000000 | 2309.815000000000 | 161.293000000000006 | 0.000 | 55 |
| M\_0.4\_F\_lh\_Set2 | 1.709 | 0 | 0.1090000000000000 | 2453.732000000000 | 305.209000000000003 | 0.000 | 69 |
| M\_0.4\_F\_lh\_Set3 | 1.718 | 0 | 0.1090000000000000 | 2414.353000000000 | 265.831000000000017 | 0.000 | 67 |
| M\_0.4\_F\_qp\_Set1 | 1.633 | 0 | 0.0730000000000000 | 2198.573000000000 | 50.049999999999997 | 0.000 | 18 |
| M\_0.4\_F\_qp\_Set2 | 1.645 | 0 | 0.0730000000000000 | 2241.143000000000 | 92.620999999999995 | 0.000 | 32 |
| M\_0.4\_F\_qp\_Set3 | 1.654 | 0 | 0.0730000000000000 | 2285.116000000000 | 136.592999999999989 | 0.000 | 43 |
| M\_0.4\_F\_qt\_Set1 | 1.714 | 0 | 0.1450000000000000 | 5144.030000000000 | 2995.507999999999811 | 0.000 | 101 |
| M\_0.4\_F\_qt\_Set2 | 1.719 | 0 | 0.2730000000000000 | NA | NA | NA | 125 |
| M\_0.4\_F\_qt\_Set3 | 1.716 | 0 | 0.2910000000000000 | NA | NA | NA | 145 |
| M\_0.4\_F\_qh\_Set1 | 1.709 | 0 | 0.1090000000000000 | 2326.635000000000 | 178.113000000000000 | 0.000 | 57 |
| M\_0.4\_F\_qh\_Set2 | 1.706 | 0 | 0.1270000000000000 | 2362.602000000000 | 214.080000000000013 | 0.000 | 62 |
| M\_0.4\_F\_qh\_Set3 | 1.720 | 0 | 0.0910000000000000 | 2363.604000000000 | 215.081999999999994 | 0.000 | 63 |
| M\_0.4\_F\_pt\_Set1 | 1.707 | 0 | 0.1640000000000000 | 4394.489000000000 | 2245.967000000000098 | 0.000 | 99 |
| M\_0.4\_F\_pt\_Set2 | 1.707 | 0 | 0.2360000000000000 | NA | NA | NA | 125 |
| M\_0.4\_F\_pt\_Set3 | 1.720 | 0 | 0.2910000000000000 | NA | NA | NA | 145 |
| M\_0.4\_F\_ph\_Set1 | 1.701 | 0 | 0.0910000000000000 | 2267.173000000000 | 118.650000000000006 | 0.000 | 50 |
| M\_0.4\_F\_ph\_Set2 | 1.712 | 0 | 0.1270000000000000 | 2538.666000000000 | 390.144000000000005 | 0.000 | 74 |
| M\_0.4\_F\_ph\_Set3 | 1.719 | 0 | 0.1090000000000000 | 2776.052000000000 | 627.529999999999973 | 0.000 | 83 |
| M\_0.4\_F\_th\_Set1 | 1.719 | 0 | 0.1820000000000000 | 9626.496999999999 | 7477.975000000000364 | 0.000 | 105 |
| M\_0.4\_F\_th\_Set2 | 1.711 | 0 | 0.2730000000000000 | NA | NA | NA | 133 |
| M\_0.4\_F\_th\_Set3 | 1.708 | 0 | 0.3450000000000000 | NA | NA | NA | 147 |
| M\_0.4\_F\_lqp\_Set1 | 1.659 | 0 | 0.0730000000000000 | 2189.600000000000 | 41.076999999999998 | 0.000 | 21 |
| M\_0.4\_F\_lqp\_Set2 | 1.651 | 0 | 0.0730000000000000 | 2199.784000000000 | 51.262000000000000 | 0.000 | 26 |
| M\_0.4\_F\_lqp\_Set3 | 1.655 | 0 | 0.0730000000000000 | 2317.686000000000 | 169.163000000000011 | 0.000 | 50 |
| M\_0.4\_F\_lqt\_Set1 | 1.719 | 0 | 0.1450000000000000 | 6489.298000000000 | 4340.775999999999840 | 0.000 | 103 |
| M\_0.4\_F\_lqt\_Set2 | 1.719 | 0 | 0.2730000000000000 | NA | NA | NA | 125 |
| M\_0.4\_F\_lqt\_Set3 | 1.724 | 0 | 0.2910000000000000 | NA | NA | NA | 145 |
| M\_0.4\_F\_lqh\_Set1 | 1.711 | 0 | 0.1090000000000000 | 2336.032000000000 | 187.508999999999986 | 0.000 | 58 |
| M\_0.4\_F\_lqh\_Set2 | 1.708 | 0 | 0.1090000000000000 | 2386.002000000000 | 237.479999999999990 | 0.000 | 64 |
| M\_0.4\_F\_lqh\_Set3 | 1.720 | 0 | 0.0910000000000000 | 2444.101000000000 | 295.577999999999975 | 0.000 | 69 |
| M\_0.4\_F\_lpt\_Set1 | 1.707 | 0 | 0.1640000000000000 | 4133.145000000000 | 1984.622000000000071 | 0.000 | 98 |
| M\_0.4\_F\_lpt\_Set2 | 1.709 | 0 | 0.2730000000000000 | NA | NA | NA | 125 |
| M\_0.4\_F\_lpt\_Set3 | 1.714 | 0 | 0.2910000000000000 | NA | NA | NA | 145 |
| M\_0.4\_F\_lph\_Set1 | 1.705 | 0 | 0.0910000000000000 | 2322.616000000000 | 174.093999999999994 | 0.000 | 57 |
| M\_0.4\_F\_lph\_Set2 | 1.713 | 0 | 0.1270000000000000 | 2451.437000000000 | 302.915000000000020 | 0.000 | 69 |
| M\_0.4\_F\_lph\_Set3 | 1.723 | 0 | 0.1090000000000000 | 2708.207000000000 | 559.683999999999969 | 0.000 | 81 |
| M\_0.4\_F\_qpt\_Set1 | 1.715 | 0 | 0.1270000000000000 | 4721.744000000000 | 2573.222000000000207 | 0.000 | 100 |
| M\_0.4\_F\_qpt\_Set2 | 1.717 | 0 | 0.2550000000000000 | NA | NA | NA | 125 |
| M\_0.4\_F\_qpt\_Set3 | 1.717 | 0 | 0.3090000000000000 | NA | NA | NA | 145 |
| M\_0.4\_F\_qph\_Set1 | 1.708 | 0 | 0.0910000000000000 | 2323.717000000000 | 175.194999999999993 | 0.000 | 57 |
| M\_0.4\_F\_qph\_Set2 | 1.707 | 0 | 0.1270000000000000 | 2519.906000000000 | 371.382999999999981 | 0.000 | 73 |
| M\_0.4\_F\_qph\_Set3 | 1.721 | 0 | 0.1090000000000000 | 3308.992000000000 | 1160.470000000000027 | 0.000 | 92 |
| M\_0.4\_F\_qth\_Set1 | 1.716 | 0 | 0.1820000000000000 | 9626.291999999999 | 7477.770000000000437 | 0.000 | 105 |
| M\_0.4\_F\_qth\_Set2 | 1.711 | 0 | 0.2730000000000000 | NA | NA | NA | 133 |
| M\_0.4\_F\_qth\_Set3 | 1.701 | 0 | 0.3450000000000000 | NA | NA | NA | 147 |
| M\_0.4\_F\_pth\_Set1 | 1.717 | 0 | 0.1640000000000000 | NA | NA | NA | 108 |
| M\_0.4\_F\_pth\_Set2 | 1.710 | 0 | 0.2730000000000000 | NA | NA | NA | 133 |
| M\_0.4\_F\_pth\_Set3 | 1.706 | 0 | 0.3450000000000000 | NA | NA | NA | 147 |
| M\_0.4\_F\_lqpt\_Set1 | 1.712 | 0 | 0.1270000000000000 | 5702.898000000000 | 3554.376000000000204 | 0.000 | 102 |
| M\_0.4\_F\_lqpt\_Set2 | 1.719 | 0 | 0.2550000000000000 | NA | NA | NA | 125 |
| M\_0.4\_F\_lqpt\_Set3 | 1.720 | 0 | 0.3090000000000000 | NA | NA | NA | 145 |
| M\_0.4\_F\_lqph\_Set1 | 1.710 | 0 | 0.0910000000000000 | 2331.879000000000 | 183.355999999999995 | 0.000 | 58 |
| M\_0.4\_F\_lqph\_Set2 | 1.709 | 0 | 0.1270000000000000 | 2583.637000000000 | 435.113999999999976 | 0.000 | 76 |
| M\_0.4\_F\_lqph\_Set3 | 1.728 | 0 | 0.1090000000000000 | 2861.988000000000 | 713.465000000000032 | 0.000 | 85 |
| M\_0.4\_F\_lqth\_Set1 | 1.718 | 0 | 0.1820000000000000 | 9626.291999999999 | 7477.770000000000437 | 0.000 | 105 |
| M\_0.4\_F\_lqth\_Set2 | 1.716 | 0 | 0.2730000000000000 | NA | NA | NA | 133 |
| M\_0.4\_F\_lqth\_Set3 | 1.704 | 0 | 0.3450000000000000 | NA | NA | NA | 147 |
| M\_0.4\_F\_lpth\_Set1 | 1.711 | 0 | 0.1640000000000000 | NA | NA | NA | 108 |
| M\_0.4\_F\_lpth\_Set2 | 1.718 | 0 | 0.2730000000000000 | NA | NA | NA | 133 |
| M\_0.4\_F\_lpth\_Set3 | 1.709 | 0 | 0.3450000000000000 | NA | NA | NA | 147 |
| M\_0.4\_F\_lqpth\_Set1 | 1.713 | 0 | 0.1450000000000000 | NA | NA | NA | 108 |
| M\_0.4\_F\_lqpth\_Set2 | 1.715 | 0 | 0.2730000000000000 | NA | NA | NA | 133 |
| M\_0.4\_F\_lqpth\_Set3 | 1.707 | 0 | 0.3450000000000000 | NA | NA | NA | 147 |
| M\_0.5\_F\_l\_Set1 | 1.520 | 0 | 0.0550000000000000 | 2272.208000000000 | 123.686000000000007 | 0.000 | 6 |
| M\_0.5\_F\_l\_Set2 | 1.537 | 0 | 0.0550000000000000 | 2272.528000000000 | 124.004999999999995 | 0.000 | 9 |
| M\_0.5\_F\_l\_Set3 | 1.533 | 0 | 0.0730000000000000 | 2275.987000000000 | 127.465000000000003 | 0.000 | 12 |
| M\_0.5\_F\_q\_Set1 | 1.553 | 0 | 0.0730000000000000 | 2236.662000000000 | 88.140000000000001 | 0.000 | 6 |
| M\_0.5\_F\_q\_Set2 | 1.602 | 0 | 0.0180000000000000 | 2203.154000000000 | 54.631000000000000 | 0.000 | 8 |
| M\_0.5\_F\_q\_Set3 | 1.632 | 0 | 0.0550000000000000 | 2193.248000000000 | 44.725000000000001 | 0.000 | 11 |
| M\_0.5\_F\_p\_Set1 | 1.574 | 0 | 0.0550000000000000 | 2227.764000000000 | 79.242000000000004 | 0.000 | 14 |
| M\_0.5\_F\_p\_Set2 | 1.584 | 0 | 0.0910000000000000 | 2238.854000000000 | 90.331999999999994 | 0.000 | 25 |
| M\_0.5\_F\_p\_Set3 | 1.639 | 0 | 0.0730000000000000 | 2285.158000000000 | 136.634999999999991 | 0.000 | 42 |
| M\_0.5\_F\_t\_Set1 | 1.689 | 0 | 0.1640000000000000 | 2675.252000000000 | 526.729000000000042 | 0.000 | 81 |
| M\_0.5\_F\_t\_Set2 | 1.706 | 0 | 0.2000000000000000 | 25285.010999999999 | 23136.488000000001193 | 0.000 | 107 |
| M\_0.5\_F\_t\_Set3 | 1.722 | 0 | 0.1640000000000000 | NA | NA | NA | 120 |
| M\_0.5\_F\_h\_Set1 | 1.704 | 0 | 0.0910000000000000 | 2290.506000000000 | 141.984000000000009 | 0.000 | 52 |
| M\_0.5\_F\_h\_Set2 | 1.705 | 0 | 0.1090000000000000 | 2300.642000000000 | 152.120000000000005 | 0.000 | 55 |
| M\_0.5\_F\_h\_Set3 | 1.719 | 0 | 0.1090000000000000 | 2453.032000000000 | 304.509000000000015 | 0.000 | 69 |
| M\_0.5\_F\_lq\_Set1 | 1.669 | 0 | 0.0730000000000000 | 2166.995000000000 | 18.472999999999999 | 0.000 | 12 |
| M\_0.5\_F\_lq\_Set2 | 1.669 | 0 | 0.0730000000000000 | 2169.167000000000 | 20.645000000000000 | 0.000 | 15 |
| M\_0.5\_F\_lq\_Set3 | 1.673 | 0 | 0.0730000000000000 | 2181.903000000000 | 33.380000000000003 | 0.000 | 20 |
| M\_0.5\_F\_lp\_Set1 | 1.624 | 0 | 0.0910000000000000 | 2186.251000000000 | 37.728000000000002 | 0.000 | 14 |
| M\_0.5\_F\_lp\_Set2 | 1.630 | 0 | 0.0910000000000000 | 2205.323000000000 | 56.801000000000002 | 0.000 | 24 |
| M\_0.5\_F\_lp\_Set3 | 1.649 | 0 | 0.0730000000000000 | 2290.890000000000 | 142.367999999999995 | 0.000 | 45 |
| M\_0.5\_F\_lt\_Set1 | 1.710 | 0 | 0.1640000000000000 | 2644.640000000000 | 496.117999999999995 | 0.000 | 80 |
| M\_0.5\_F\_lt\_Set2 | 1.717 | 0 | 0.1820000000000000 | 25285.010999999999 | 23136.488000000001193 | 0.000 | 107 |
| M\_0.5\_F\_lt\_Set3 | 1.725 | 0 | 0.1450000000000000 | NA | NA | NA | 120 |
| M\_0.5\_F\_lh\_Set1 | 1.705 | 0 | 0.1270000000000000 | 2276.814000000000 | 128.292000000000002 | 0.000 | 50 |
| M\_0.5\_F\_lh\_Set2 | 1.714 | 0 | 0.1090000000000000 | 2318.831000000000 | 170.307999999999993 | 0.000 | 57 |
| M\_0.5\_F\_lh\_Set3 | 1.716 | 0 | 0.1090000000000000 | 2349.441000000000 | 200.919000000000011 | 0.000 | 61 |
| M\_0.5\_F\_qp\_Set1 | 1.627 | 0 | 0.0730000000000000 | 2198.806000000000 | 50.283999999999999 | 0.000 | 18 |
| M\_0.5\_F\_qp\_Set2 | 1.645 | 0 | 0.0730000000000000 | 2229.325000000000 | 80.802999999999997 | 0.000 | 30 |
| M\_0.5\_F\_qp\_Set3 | 1.646 | 0 | 0.0730000000000000 | 2260.381000000000 | 111.858999999999995 | 0.000 | 38 |
| M\_0.5\_F\_qt\_Set1 | 1.716 | 0 | 0.1270000000000000 | 2710.233000000000 | 561.711000000000013 | 0.000 | 82 |
| M\_0.5\_F\_qt\_Set2 | 1.723 | 0 | 0.1270000000000000 | NA | NA | NA | 109 |
| M\_0.5\_F\_qt\_Set3 | 1.727 | 0 | 0.1090000000000000 | NA | NA | NA | 119 |
| M\_0.5\_F\_qh\_Set1 | 1.697 | 0 | 0.1270000000000000 | 2250.269000000000 | 101.745999999999995 | 0.000 | 46 |
| M\_0.5\_F\_qh\_Set2 | 1.707 | 0 | 0.1090000000000000 | 2277.690000000000 | 129.167000000000002 | 0.000 | 52 |
| M\_0.5\_F\_qh\_Set3 | 1.722 | 0 | 0.1090000000000000 | 2329.794000000000 | 181.270999999999987 | 0.000 | 59 |
| M\_0.5\_F\_pt\_Set1 | 1.694 | 0 | 0.1640000000000000 | 2783.719000000000 | 635.196000000000026 | 0.000 | 84 |
| M\_0.5\_F\_pt\_Set2 | 1.708 | 0 | 0.1820000000000000 | NA | NA | NA | 110 |
| M\_0.5\_F\_pt\_Set3 | 1.728 | 0 | 0.1450000000000000 | NA | NA | NA | 120 |
| M\_0.5\_F\_ph\_Set1 | 1.705 | 0 | 0.0910000000000000 | 2238.925000000000 | 90.402000000000001 | 0.000 | 45 |
| M\_0.5\_F\_ph\_Set2 | 1.707 | 0 | 0.1090000000000000 | 2363.480000000000 | 214.957999999999998 | 0.000 | 62 |
| M\_0.5\_F\_ph\_Set3 | 1.720 | 0 | 0.1090000000000000 | 2629.822000000000 | 481.300000000000011 | 0.000 | 78 |
| M\_0.5\_F\_th\_Set1 | 1.713 | 0 | 0.1450000000000000 | 2923.413000000000 | 774.889999999999986 | 0.000 | 87 |
| M\_0.5\_F\_th\_Set2 | 1.720 | 0 | 0.1640000000000000 | NA | NA | NA | 115 |
| M\_0.5\_F\_th\_Set3 | 1.725 | 0 | 0.1820000000000000 | NA | NA | NA | 127 |
| M\_0.5\_F\_lqp\_Set1 | 1.647 | 0 | 0.0730000000000000 | 2181.577000000000 | 33.055000000000000 | 0.000 | 18 |
| M\_0.5\_F\_lqp\_Set2 | 1.652 | 0 | 0.0730000000000000 | 2213.425000000000 | 64.902000000000001 | 0.000 | 29 |
| M\_0.5\_F\_lqp\_Set3 | 1.650 | 0 | 0.0730000000000000 | 2300.839000000000 | 152.317000000000007 | 0.000 | 47 |
| M\_0.5\_F\_lqt\_Set1 | 1.718 | 0 | 0.1270000000000000 | 2710.233000000000 | 561.711000000000013 | 0.000 | 82 |
| M\_0.5\_F\_lqt\_Set2 | 1.725 | 0 | 0.1270000000000000 | NA | NA | NA | 109 |
| M\_0.5\_F\_lqt\_Set3 | 1.731 | 0 | 0.1090000000000000 | NA | NA | NA | 119 |
| M\_0.5\_F\_lqh\_Set1 | 1.700 | 0 | 0.1270000000000000 | 2275.969000000000 | 127.445999999999998 | 0.000 | 50 |
| M\_0.5\_F\_lqh\_Set2 | 1.705 | 0 | 0.1090000000000000 | 2293.816000000000 | 145.293000000000006 | 0.000 | 54 |
| M\_0.5\_F\_lqh\_Set3 | 1.716 | 0 | 0.1090000000000000 | 2319.205000000000 | 170.682999999999993 | 0.000 | 58 |
| M\_0.5\_F\_lpt\_Set1 | 1.703 | 0 | 0.1640000000000000 | 2826.943000000000 | 678.421000000000049 | 0.000 | 85 |
| M\_0.5\_F\_lpt\_Set2 | 1.718 | 0 | 0.1820000000000000 | NA | NA | NA | 110 |
| M\_0.5\_F\_lpt\_Set3 | 1.733 | 0 | 0.1450000000000000 | NA | NA | NA | 120 |
| M\_0.5\_F\_lph\_Set1 | 1.704 | 0 | 0.0910000000000000 | 2264.096000000000 | 115.573999999999998 | 0.000 | 49 |
| M\_0.5\_F\_lph\_Set2 | 1.709 | 0 | 0.1090000000000000 | 2364.210000000000 | 215.687000000000012 | 0.000 | 62 |
| M\_0.5\_F\_lph\_Set3 | 1.724 | 0 | 0.1090000000000000 | 2826.635000000000 | 678.113000000000056 | 0.000 | 84 |
| M\_0.5\_F\_qpt\_Set1 | 1.714 | 0 | 0.1270000000000000 | 2708.946000000000 | 560.423000000000002 | 0.000 | 82 |
| M\_0.5\_F\_qpt\_Set2 | 1.723 | 0 | 0.1640000000000000 | NA | NA | NA | 108 |
| M\_0.5\_F\_qpt\_Set3 | 1.728 | 0 | 0.1270000000000000 | NA | NA | NA | 119 |
| M\_0.5\_F\_qph\_Set1 | 1.705 | 0 | 0.0910000000000000 | 2294.613000000000 | 146.091000000000008 | 0.000 | 53 |
| M\_0.5\_F\_qph\_Set2 | 1.707 | 0 | 0.1090000000000000 | 2354.609000000000 | 206.086999999999989 | 0.000 | 61 |
| M\_0.5\_F\_qph\_Set3 | 1.720 | 0 | 0.1090000000000000 | 2559.746000000000 | 411.223000000000013 | 0.000 | 75 |
| M\_0.5\_F\_qth\_Set1 | 1.711 | 0 | 0.1450000000000000 | 2978.649000000000 | 830.126999999999953 | 0.000 | 88 |
| M\_0.5\_F\_qth\_Set2 | 1.717 | 0 | 0.1270000000000000 | NA | NA | NA | 119 |
| M\_0.5\_F\_qth\_Set3 | 1.721 | 0 | 0.1640000000000000 | NA | NA | NA | 127 |
| M\_0.5\_F\_pth\_Set1 | 1.713 | 0 | 0.1450000000000000 | 3039.370000000000 | 890.846999999999980 | 0.000 | 89 |
| M\_0.5\_F\_pth\_Set2 | 1.728 | 0 | 0.1640000000000000 | NA | NA | NA | 115 |
| M\_0.5\_F\_pth\_Set3 | 1.715 | 0 | 0.1820000000000000 | NA | NA | NA | 127 |
| M\_0.5\_F\_lqpt\_Set1 | 1.710 | 0 | 0.1270000000000000 | 2708.946000000000 | 560.423000000000002 | 0.000 | 82 |
| M\_0.5\_F\_lqpt\_Set2 | 1.718 | 0 | 0.1640000000000000 | NA | NA | NA | 110 |
| M\_0.5\_F\_lqpt\_Set3 | 1.732 | 0 | 0.1270000000000000 | NA | NA | NA | 119 |
| M\_0.5\_F\_lqph\_Set1 | 1.704 | 0 | 0.1090000000000000 | 2319.252000000000 | 170.729000000000013 | 0.000 | 56 |
| M\_0.5\_F\_lqph\_Set2 | 1.708 | 0 | 0.1090000000000000 | 2355.013000000000 | 206.491000000000014 | 0.000 | 61 |
| M\_0.5\_F\_lqph\_Set3 | 1.718 | 0 | 0.1090000000000000 | 2556.948000000000 | 408.425999999999988 | 0.000 | 75 |
| M\_0.5\_F\_lqth\_Set1 | 1.714 | 0 | 0.1450000000000000 | 3039.871000000000 | 891.349000000000046 | 0.000 | 89 |
| M\_0.5\_F\_lqth\_Set2 | 1.720 | 0 | 0.1640000000000000 | NA | NA | NA | 119 |
| M\_0.5\_F\_lqth\_Set3 | 1.720 | 0 | 0.1640000000000000 | NA | NA | NA | 127 |
| M\_0.5\_F\_lpth\_Set1 | 1.713 | 0 | 0.1450000000000000 | 2977.968000000000 | 829.446000000000026 | 0.000 | 88 |
| M\_0.5\_F\_lpth\_Set2 | 1.724 | 0 | 0.1640000000000000 | NA | NA | NA | 115 |
| M\_0.5\_F\_lpth\_Set3 | 1.719 | 0 | 0.1820000000000000 | NA | NA | NA | 127 |
| M\_0.5\_F\_lqpth\_Set1 | 1.716 | 0 | 0.1450000000000000 | 3039.303000000000 | 890.780999999999949 | 0.000 | 89 |
| M\_0.5\_F\_lqpth\_Set2 | 1.721 | 0 | 0.1450000000000000 | NA | NA | NA | 119 |
| M\_0.5\_F\_lqpth\_Set3 | 1.719 | 0 | 0.1820000000000000 | NA | NA | NA | 127 |
| M\_0.6\_F\_l\_Set1 | 1.516 | 0 | 0.0730000000000000 | 2272.241000000000 | 123.718999999999994 | 0.000 | 6 |
| M\_0.6\_F\_l\_Set2 | 1.522 | 0 | 0.0730000000000000 | 2270.754000000000 | 122.231999999999999 | 0.000 | 8 |
| M\_0.6\_F\_l\_Set3 | 1.533 | 0 | 0.0730000000000000 | 2277.608000000000 | 129.085000000000008 | 0.000 | 12 |
| M\_0.6\_F\_q\_Set1 | 1.558 | 0 | 0.0730000000000000 | 2236.716000000000 | 88.192999999999998 | 0.000 | 6 |
| M\_0.6\_F\_q\_Set2 | 1.601 | 0 | 0.0180000000000000 | 2202.045000000000 | 53.523000000000003 | 0.000 | 7 |
| M\_0.6\_F\_q\_Set3 | 1.626 | 0 | 0.0550000000000000 | 2191.873000000000 | 43.350000000000001 | 0.000 | 10 |
| M\_0.6\_F\_p\_Set1 | 1.571 | 0 | 0.0550000000000000 | 2224.151000000000 | 75.628000000000000 | 0.000 | 12 |
| M\_0.6\_F\_p\_Set2 | 1.587 | 0 | 0.0910000000000000 | 2236.092000000000 | 87.569000000000003 | 0.000 | 24 |
| M\_0.6\_F\_p\_Set3 | 1.641 | 0 | 0.0730000000000000 | 2255.075000000000 | 106.552999999999997 | 0.000 | 35 |
| M\_0.6\_F\_t\_Set1 | 1.693 | 0 | 0.1820000000000000 | 2439.132000000000 | 290.610000000000014 | 0.000 | 70 |
| M\_0.6\_F\_t\_Set2 | 1.694 | 0 | 0.1450000000000000 | 3577.064000000000 | 1428.541999999999916 | 0.000 | 95 |
| M\_0.6\_F\_t\_Set3 | 1.712 | 0 | 0.1450000000000000 | 4353.734000000000 | 2205.211999999999989 | 0.000 | 99 |
| M\_0.6\_F\_h\_Set1 | 1.698 | 0 | 0.1090000000000000 | 2267.116000000000 | 118.593999999999994 | 0.000 | 48 |
| M\_0.6\_F\_h\_Set2 | 1.700 | 0 | 0.1270000000000000 | 2283.377000000000 | 134.854000000000013 | 0.000 | 52 |
| M\_0.6\_F\_h\_Set3 | 1.716 | 0 | 0.1090000000000000 | 2325.996000000000 | 177.473000000000013 | 0.000 | 58 |
| M\_0.6\_F\_lq\_Set1 | 1.670 | 0 | 0.0550000000000000 | 2168.459000000000 | 19.936000000000000 | 0.000 | 12 |
| M\_0.6\_F\_lq\_Set2 | 1.667 | 0 | 0.0550000000000000 | 2173.216000000000 | 24.693000000000001 | 0.000 | 16 |
| M\_0.6\_F\_lq\_Set3 | 1.669 | 0 | 0.0730000000000000 | 2179.640000000000 | 31.117999999999999 | 0.000 | 18 |
| M\_0.6\_F\_lp\_Set1 | 1.621 | 0 | 0.0910000000000000 | 2191.951000000000 | 43.429000000000002 | 0.000 | 16 |
| M\_0.6\_F\_lp\_Set2 | 1.629 | 0 | 0.0910000000000000 | 2226.614000000000 | 78.090999999999994 | 0.000 | 29 |
| M\_0.6\_F\_lp\_Set3 | 1.652 | 0 | 0.0730000000000000 | 2270.366000000000 | 121.843999999999994 | 0.000 | 40 |
| M\_0.6\_F\_lt\_Set1 | 1.707 | 0 | 0.1450000000000000 | 2476.037000000000 | 327.514000000000010 | 0.000 | 72 |
| M\_0.6\_F\_lt\_Set2 | 1.711 | 0 | 0.1640000000000000 | 3729.792000000000 | 1581.269000000000005 | 0.000 | 96 |
| M\_0.6\_F\_lt\_Set3 | 1.723 | 0 | 0.1450000000000000 | 5662.198000000000 | 3513.675000000000182 | 0.000 | 102 |
| M\_0.6\_F\_lh\_Set1 | 1.698 | 0 | 0.1090000000000000 | 2211.045000000000 | 62.523000000000003 | 0.000 | 38 |
| M\_0.6\_F\_lh\_Set2 | 1.706 | 0 | 0.0910000000000000 | 2297.777000000000 | 149.253999999999991 | 0.000 | 54 |
| M\_0.6\_F\_lh\_Set3 | 1.715 | 0 | 0.1090000000000000 | 2281.073000000000 | 132.550999999999988 | 0.000 | 53 |
| M\_0.6\_F\_qp\_Set1 | 1.628 | 0 | 0.0730000000000000 | 2196.426000000000 | 47.902999999999999 | 0.000 | 17 |
| M\_0.6\_F\_qp\_Set2 | 1.640 | 0 | 0.0730000000000000 | 2217.327000000000 | 68.805000000000007 | 0.000 | 26 |
| M\_0.6\_F\_qp\_Set3 | 1.653 | 0 | 0.0730000000000000 | 2273.928000000000 | 125.406000000000006 | 0.000 | 41 |
| M\_0.6\_F\_qt\_Set1 | 1.716 | 0 | 0.1090000000000000 | 2442.888000000000 | 294.365999999999985 | 0.000 | 70 |
| M\_0.6\_F\_qt\_Set2 | 1.717 | 0 | 0.1270000000000000 | 4388.036000000000 | 2239.514000000000124 | 0.000 | 99 |
| M\_0.6\_F\_qt\_Set3 | 1.729 | 0 | 0.0910000000000000 | 7624.725000000000 | 5476.202000000000226 | 0.000 | 104 |
| M\_0.6\_F\_qh\_Set1 | 1.700 | 0 | 0.1090000000000000 | 2198.317000000000 | 49.793999999999997 | 0.000 | 35 |
| M\_0.6\_F\_qh\_Set2 | 1.704 | 0 | 0.0910000000000000 | 2241.333000000000 | 92.810000000000002 | 0.000 | 46 |
| M\_0.6\_F\_qh\_Set3 | 1.718 | 0 | 0.0910000000000000 | 2274.048000000000 | 125.525000000000006 | 0.000 | 52 |
| M\_0.6\_F\_pt\_Set1 | 1.691 | 0 | 0.1270000000000000 | 2491.592000000000 | 343.069000000000017 | 0.000 | 73 |
| M\_0.6\_F\_pt\_Set2 | 1.711 | 0 | 0.1820000000000000 | 4385.515000000000 | 2236.992999999999938 | 0.000 | 99 |
| M\_0.6\_F\_pt\_Set3 | 1.718 | 0 | 0.1450000000000000 | NA | NA | NA | 111 |
| M\_0.6\_F\_ph\_Set1 | 1.698 | 0 | 0.1090000000000000 | 2196.684000000000 | 48.161999999999999 | 0.000 | 36 |
| M\_0.6\_F\_ph\_Set2 | 1.706 | 0 | 0.1270000000000000 | 2285.143000000000 | 136.620000000000005 | 0.000 | 53 |
| M\_0.6\_F\_ph\_Set3 | 1.720 | 0 | 0.1090000000000000 | 2373.871000000000 | 225.348999999999990 | 0.000 | 63 |
| M\_0.6\_F\_th\_Set1 | 1.712 | 0 | 0.1270000000000000 | 2513.361000000000 | 364.838999999999999 | 0.000 | 74 |
| M\_0.6\_F\_th\_Set2 | 1.717 | 0 | 0.1450000000000000 | 4716.003000000000 | 2567.480000000000018 | 0.000 | 100 |
| M\_0.6\_F\_th\_Set3 | 1.722 | 0 | 0.1270000000000000 | NA | NA | NA | 110 |
| M\_0.6\_F\_lqp\_Set1 | 1.650 | 0 | 0.0730000000000000 | 2181.676000000000 | 33.154000000000003 | 0.000 | 18 |
| M\_0.6\_F\_lqp\_Set2 | 1.650 | 0 | 0.0730000000000000 | 2217.250000000000 | 68.727999999999994 | 0.000 | 30 |
| M\_0.6\_F\_lqp\_Set3 | 1.648 | 0 | 0.0730000000000000 | 2268.419000000000 | 119.896000000000001 | 0.000 | 41 |
| M\_0.6\_F\_lqt\_Set1 | 1.722 | 0 | 0.1090000000000000 | 2459.463000000000 | 310.939999999999998 | 0.000 | 71 |
| M\_0.6\_F\_lqt\_Set2 | 1.720 | 0 | 0.1270000000000000 | 4388.036000000000 | 2239.514000000000124 | 0.000 | 99 |
| M\_0.6\_F\_lqt\_Set3 | 1.725 | 0 | 0.0910000000000000 | 7624.725000000000 | 5476.202000000000226 | 0.000 | 104 |
| M\_0.6\_F\_lqh\_Set1 | 1.698 | 0 | 0.1090000000000000 | 2221.363000000000 | 72.840000000000003 | 0.000 | 40 |
| M\_0.6\_F\_lqh\_Set2 | 1.710 | 0 | 0.0910000000000000 | 2260.197000000000 | 111.674999999999997 | 0.000 | 49 |
| M\_0.6\_F\_lqh\_Set3 | 1.719 | 0 | 0.0910000000000000 | 2273.970000000000 | 125.447999999999993 | 0.000 | 52 |
| M\_0.6\_F\_lpt\_Set1 | 1.698 | 0 | 0.1270000000000000 | 2492.147000000000 | 343.625000000000000 | 0.000 | 73 |
| M\_0.6\_F\_lpt\_Set2 | 1.713 | 0 | 0.1820000000000000 | 4385.032000000000 | 2236.510000000000218 | 0.000 | 99 |
| M\_0.6\_F\_lpt\_Set3 | 1.729 | 0 | 0.1640000000000000 | NA | NA | NA | 112 |
| M\_0.6\_F\_lph\_Set1 | 1.697 | 0 | 0.1090000000000000 | 2206.487000000000 | 57.965000000000003 | 0.000 | 38 |
| M\_0.6\_F\_lph\_Set2 | 1.703 | 0 | 0.1270000000000000 | 2347.574000000000 | 199.051999999999992 | 0.000 | 60 |
| M\_0.6\_F\_lph\_Set3 | 1.716 | 0 | 0.1090000000000000 | 2453.453000000000 | 304.930000000000007 | 0.000 | 69 |
| M\_0.6\_F\_qpt\_Set1 | 1.708 | 0 | 0.1090000000000000 | 2512.918000000000 | 364.396000000000015 | 0.000 | 74 |
| M\_0.6\_F\_qpt\_Set2 | 1.716 | 0 | 0.1450000000000000 | 5135.710000000000 | 2987.188000000000102 | 0.000 | 101 |
| M\_0.6\_F\_qpt\_Set3 | 1.730 | 0 | 0.1640000000000000 | 25283.596000000001 | 23135.074000000000524 | 0.000 | 107 |
| M\_0.6\_F\_qph\_Set1 | 1.703 | 0 | 0.1090000000000000 | 2216.379000000000 | 67.856999999999999 | 0.000 | 40 |
| M\_0.6\_F\_qph\_Set2 | 1.707 | 0 | 0.0910000000000000 | 2262.683000000000 | 114.161000000000001 | 0.000 | 50 |
| M\_0.6\_F\_qph\_Set3 | 1.719 | 0 | 0.1090000000000000 | 2397.792000000000 | 249.269000000000005 | 0.000 | 65 |
| M\_0.6\_F\_qth\_Set1 | 1.718 | 0 | 0.1640000000000000 | 2557.610000000000 | 409.088000000000022 | 0.000 | 76 |
| M\_0.6\_F\_qth\_Set2 | 1.714 | 0 | 0.1450000000000000 | 5137.985000000000 | 2989.461999999999989 | 0.000 | 101 |
| M\_0.6\_F\_qth\_Set3 | 1.720 | 0 | 0.1450000000000000 | NA | NA | NA | 110 |
| M\_0.6\_F\_pth\_Set1 | 1.718 | 0 | 0.1270000000000000 | 2472.627000000000 | 324.103999999999985 | 0.000 | 72 |
| M\_0.6\_F\_pth\_Set2 | 1.718 | 0 | 0.1270000000000000 | 6481.354000000000 | 4332.831000000000131 | 0.000 | 103 |
| M\_0.6\_F\_pth\_Set3 | 1.724 | 0 | 0.1450000000000000 | NA | NA | NA | 113 |
| M\_0.6\_F\_lqpt\_Set1 | 1.710 | 0 | 0.1090000000000000 | 2512.918000000000 | 364.396000000000015 | 0.000 | 74 |
| M\_0.6\_F\_lqpt\_Set2 | 1.712 | 0 | 0.1450000000000000 | 5696.302000000000 | 3547.778999999999996 | 0.000 | 102 |
| M\_0.6\_F\_lqpt\_Set3 | 1.725 | 0 | 0.1640000000000000 | 25283.596000000001 | 23135.074000000000524 | 0.000 | 107 |
| M\_0.6\_F\_lqph\_Set1 | 1.702 | 0 | 0.0910000000000000 | 2233.157000000000 | 84.634000000000000 | 0.000 | 43 |
| M\_0.6\_F\_lqph\_Set2 | 1.702 | 0 | 0.0910000000000000 | 2319.646000000000 | 171.123999999999995 | 0.000 | 57 |
| M\_0.6\_F\_lqph\_Set3 | 1.717 | 0 | 0.1090000000000000 | 2422.492000000000 | 273.970000000000027 | 0.000 | 67 |
| M\_0.6\_F\_lqth\_Set1 | 1.717 | 0 | 0.1450000000000000 | 2557.252000000000 | 408.730000000000018 | 0.000 | 76 |
| M\_0.6\_F\_lqth\_Set2 | 1.714 | 0 | 0.1270000000000000 | 5138.898000000000 | 2990.376000000000204 | 0.000 | 101 |
| M\_0.6\_F\_lqth\_Set3 | 1.717 | 0 | 0.1450000000000000 | NA | NA | NA | 110 |
| M\_0.6\_F\_lpth\_Set1 | 1.716 | 0 | 0.1270000000000000 | 2555.280000000000 | 406.757999999999981 | 0.000 | 76 |
| M\_0.6\_F\_lpth\_Set2 | 1.723 | 0 | 0.1270000000000000 | 9621.008000000000 | 7472.484999999999673 | 0.000 | 105 |
| M\_0.6\_F\_lpth\_Set3 | 1.722 | 0 | 0.1450000000000000 | NA | NA | NA | 113 |
| M\_0.6\_F\_lqpth\_Set1 | 1.723 | 0 | 0.0910000000000000 | 2554.234000000000 | 405.711000000000013 | 0.000 | 76 |
| M\_0.6\_F\_lqpth\_Set2 | 1.724 | 0 | 0.1270000000000000 | 25317.314999999999 | 23168.793000000001484 | 0.000 | 107 |
| M\_0.6\_F\_lqpth\_Set3 | 1.718 | 0 | 0.1450000000000000 | NA | NA | NA | 112 |
| M\_0.7\_F\_l\_Set1 | 1.519 | 0 | 0.0730000000000000 | 2272.284000000000 | 123.760999999999996 | 0.000 | 6 |
| M\_0.7\_F\_l\_Set2 | 1.532 | 0 | 0.0730000000000000 | 2271.240000000000 | 122.716999999999999 | 0.000 | 8 |
| M\_0.7\_F\_l\_Set3 | 1.527 | 0 | 0.0730000000000000 | 2276.450000000000 | 127.927000000000007 | 0.000 | 11 |
| M\_0.7\_F\_q\_Set1 | 1.547 | 0 | 0.0730000000000000 | 2236.770000000000 | 88.248000000000005 | 0.000 | 6 |
| M\_0.7\_F\_q\_Set2 | 1.602 | 0 | 0.0180000000000000 | 2202.128000000000 | 53.604999999999997 | 0.000 | 7 |
| M\_0.7\_F\_q\_Set3 | 1.634 | 0 | 0.0550000000000000 | 2191.963000000000 | 43.441000000000003 | 0.000 | 10 |
| M\_0.7\_F\_p\_Set1 | 1.562 | 0 | 0.0550000000000000 | 2224.655000000000 | 76.132000000000005 | 0.000 | 12 |
| M\_0.7\_F\_p\_Set2 | 1.575 | 0 | 0.0910000000000000 | 2229.651000000000 | 81.129000000000005 | 0.000 | 22 |
| M\_0.7\_F\_p\_Set3 | 1.638 | 0 | 0.0730000000000000 | 2261.977000000000 | 113.453999999999994 | 0.000 | 37 |
| M\_0.7\_F\_t\_Set1 | 1.682 | 0 | 0.1450000000000000 | 2313.031000000000 | 164.508999999999986 | 0.000 | 59 |
| M\_0.7\_F\_t\_Set2 | 1.695 | 0 | 0.1450000000000000 | 2694.861000000000 | 546.337999999999965 | 0.000 | 82 |
| M\_0.7\_F\_t\_Set3 | 1.701 | 0 | 0.1450000000000000 | 3332.957000000000 | 1184.433999999999969 | 0.000 | 93 |
| M\_0.7\_F\_h\_Set1 | 1.698 | 0 | 0.1090000000000000 | 2230.635000000000 | 82.111999999999995 | 0.000 | 41 |
| M\_0.7\_F\_h\_Set2 | 1.701 | 0 | 0.1090000000000000 | 2230.402000000000 | 81.879000000000005 | 0.000 | 43 |
| M\_0.7\_F\_h\_Set3 | 1.708 | 0 | 0.1090000000000000 | 2266.208000000000 | 117.685000000000002 | 0.000 | 50 |
| M\_0.7\_F\_lq\_Set1 | 1.667 | 0 | 0.0550000000000000 | 2170.776000000000 | 22.253000000000000 | 0.000 | 12 |
| M\_0.7\_F\_lq\_Set2 | 1.664 | 0 | 0.0550000000000000 | 2171.940000000000 | 23.417999999999999 | 0.000 | 15 |
| M\_0.7\_F\_lq\_Set3 | 1.670 | 0 | 0.0730000000000000 | 2176.740000000000 | 28.218000000000000 | 0.000 | 16 |
| M\_0.7\_F\_lp\_Set1 | 1.611 | 0 | 0.0730000000000000 | 2185.393000000000 | 36.871000000000002 | 0.000 | 14 |
| M\_0.7\_F\_lp\_Set2 | 1.615 | 0 | 0.0910000000000000 | 2229.286000000000 | 80.763999999999996 | 0.000 | 29 |
| M\_0.7\_F\_lp\_Set3 | 1.646 | 0 | 0.0730000000000000 | 2229.488000000000 | 80.965000000000003 | 0.000 | 31 |
| M\_0.7\_F\_lt\_Set1 | 1.710 | 0 | 0.1090000000000000 | 2295.375000000000 | 146.852000000000004 | 0.000 | 57 |
| M\_0.7\_F\_lt\_Set2 | 1.712 | 0 | 0.1270000000000000 | 2698.871000000000 | 550.349000000000046 | 0.000 | 82 |
| M\_0.7\_F\_lt\_Set3 | 1.718 | 0 | 0.0910000000000000 | 3336.257000000000 | 1187.733999999999924 | 0.000 | 93 |
| M\_0.7\_F\_lh\_Set1 | 1.696 | 0 | 0.1090000000000000 | 2187.683000000000 | 39.159999999999997 | 0.000 | 32 |
| M\_0.7\_F\_lh\_Set2 | 1.704 | 0 | 0.1090000000000000 | 2212.186000000000 | 63.662999999999997 | 0.000 | 40 |
| M\_0.7\_F\_lh\_Set3 | 1.712 | 0 | 0.1090000000000000 | 2236.716000000000 | 88.192999999999998 | 0.000 | 46 |
| M\_0.7\_F\_qp\_Set1 | 1.627 | 0 | 0.0730000000000000 | 2197.425000000000 | 48.902000000000001 | 0.000 | 17 |
| M\_0.7\_F\_qp\_Set2 | 1.629 | 0 | 0.0730000000000000 | 2215.442000000000 | 66.920000000000002 | 0.000 | 25 |
| M\_0.7\_F\_qp\_Set3 | 1.645 | 0 | 0.0730000000000000 | 2254.824000000000 | 106.302000000000007 | 0.000 | 36 |
| M\_0.7\_F\_qt\_Set1 | 1.720 | 0 | 0.1090000000000000 | 2280.332000000000 | 131.810000000000002 | 0.000 | 55 |
| M\_0.7\_F\_qt\_Set2 | 1.718 | 0 | 0.1090000000000000 | 2636.538000000000 | 488.014999999999986 | 0.000 | 80 |
| M\_0.7\_F\_qt\_Set3 | 1.730 | 0 | 0.1090000000000000 | 2946.491000000000 | 797.967999999999961 | 0.000 | 88 |
| M\_0.7\_F\_qh\_Set1 | 1.699 | 0 | 0.1090000000000000 | 2176.240000000000 | 27.718000000000000 | 0.000 | 29 |
| M\_0.7\_F\_qh\_Set2 | 1.708 | 0 | 0.1090000000000000 | 2223.577000000000 | 75.055000000000007 | 0.000 | 42 |
| M\_0.7\_F\_qh\_Set3 | 1.714 | 0 | 0.1090000000000000 | 2278.539000000000 | 130.015999999999991 | 0.000 | 52 |
| M\_0.7\_F\_pt\_Set1 | 1.695 | 0 | 0.1270000000000000 | 2391.913000000000 | 243.389999999999986 | 0.000 | 66 |
| M\_0.7\_F\_pt\_Set2 | 1.702 | 0 | 0.1450000000000000 | 2818.916000000000 | 670.394000000000005 | 0.000 | 85 |
| M\_0.7\_F\_pt\_Set3 | 1.708 | 0 | 0.1090000000000000 | 2840.304000000000 | 691.782000000000039 | 0.000 | 86 |
| M\_0.7\_F\_ph\_Set1 | 1.699 | 0 | 0.1090000000000000 | 2214.621000000000 | 66.099000000000004 | 0.000 | 39 |
| M\_0.7\_F\_ph\_Set2 | 1.702 | 0 | 0.1090000000000000 | 2306.776000000000 | 158.252999999999986 | 0.000 | 55 |
| M\_0.7\_F\_ph\_Set3 | 1.714 | 0 | 0.1090000000000000 | 2325.053000000000 | 176.531000000000006 | 0.000 | 58 |
| M\_0.7\_F\_th\_Set1 | 1.713 | 0 | 0.1450000000000000 | 2357.797000000000 | 209.274000000000001 | 0.000 | 63 |
| M\_0.7\_F\_th\_Set2 | 1.715 | 0 | 0.1270000000000000 | 2822.136000000000 | 673.613000000000056 | 0.000 | 85 |
| M\_0.7\_F\_th\_Set3 | 1.721 | 0 | 0.1640000000000000 | 3238.891000000000 | 1090.368999999999915 | 0.000 | 92 |
| M\_0.7\_F\_lqp\_Set1 | 1.646 | 0 | 0.0730000000000000 | 2183.810000000000 | 35.287999999999997 | 0.000 | 18 |
| M\_0.7\_F\_lqp\_Set2 | 1.651 | 0 | 0.0730000000000000 | 2201.600000000000 | 53.076999999999998 | 0.000 | 25 |
| M\_0.7\_F\_lqp\_Set3 | 1.644 | 0 | 0.0730000000000000 | 2277.240000000000 | 128.717999999999989 | 0.000 | 42 |
| M\_0.7\_F\_lqt\_Set1 | 1.718 | 0 | 0.1090000000000000 | 2280.124000000000 | 131.602000000000004 | 0.000 | 55 |
| M\_0.7\_F\_lqt\_Set2 | 1.723 | 0 | 0.1090000000000000 | 2668.408000000000 | 519.884999999999991 | 0.000 | 81 |
| M\_0.7\_F\_lqt\_Set3 | 1.731 | 0 | 0.1090000000000000 | 3008.797000000000 | 860.274999999999977 | 0.000 | 89 |
| M\_0.7\_F\_lqh\_Set1 | 1.701 | 0 | 0.1090000000000000 | 2205.421000000000 | 56.898000000000003 | 0.000 | 36 |
| M\_0.7\_F\_lqh\_Set2 | 1.709 | 0 | 0.1090000000000000 | 2272.527000000000 | 124.004000000000005 | 0.000 | 50 |
| M\_0.7\_F\_lqh\_Set3 | 1.713 | 0 | 0.0910000000000000 | 2285.770000000000 | 137.247000000000014 | 0.000 | 53 |
| M\_0.7\_F\_lpt\_Set1 | 1.703 | 0 | 0.1270000000000000 | 2393.129000000000 | 244.605999999999995 | 0.000 | 66 |
| M\_0.7\_F\_lpt\_Set2 | 1.706 | 0 | 0.1450000000000000 | 2776.770000000000 | 628.246999999999957 | 0.000 | 84 |
| M\_0.7\_F\_lpt\_Set3 | 1.716 | 0 | 0.1090000000000000 | 3077.857000000000 | 929.333999999999946 | 0.000 | 90 |
| M\_0.7\_F\_lph\_Set1 | 1.699 | 0 | 0.1090000000000000 | 2213.997000000000 | 65.474999999999994 | 0.000 | 39 |
| M\_0.7\_F\_lph\_Set2 | 1.699 | 0 | 0.1090000000000000 | 2297.357000000000 | 148.835000000000008 | 0.000 | 54 |
| M\_0.7\_F\_lph\_Set3 | 1.713 | 0 | 0.1090000000000000 | 2315.517000000000 | 166.995000000000005 | 0.000 | 57 |
| M\_0.7\_F\_qpt\_Set1 | 1.712 | 0 | 0.1090000000000000 | 2333.928000000000 | 185.406000000000006 | 0.000 | 61 |
| M\_0.7\_F\_qpt\_Set2 | 1.712 | 0 | 0.1270000000000000 | 2702.240000000000 | 553.717999999999961 | 0.000 | 82 |
| M\_0.7\_F\_qpt\_Set3 | 1.719 | 0 | 0.1090000000000000 | 3340.114000000000 | 1191.590999999999894 | 0.000 | 93 |
| M\_0.7\_F\_qph\_Set1 | 1.701 | 0 | 0.1090000000000000 | 2191.942000000000 | 43.418999999999997 | 0.000 | 34 |
| M\_0.7\_F\_qph\_Set2 | 1.704 | 0 | 0.1090000000000000 | 2255.526000000000 | 107.003000000000000 | 0.000 | 48 |
| M\_0.7\_F\_qph\_Set3 | 1.719 | 0 | 0.1090000000000000 | 2314.353000000000 | 165.830999999999989 | 0.000 | 57 |
| M\_0.7\_F\_qth\_Set1 | 1.716 | 0 | 0.1820000000000000 | 2316.909000000000 | 168.385999999999996 | 0.000 | 59 |
| M\_0.7\_F\_qth\_Set2 | 1.717 | 0 | 0.1270000000000000 | 2584.967000000000 | 436.444000000000017 | 0.000 | 78 |
| M\_0.7\_F\_qth\_Set3 | 1.723 | 0 | 0.1640000000000000 | 3154.881000000000 | 1006.357999999999947 | 0.000 | 91 |
| M\_0.7\_F\_pth\_Set1 | 1.711 | 0 | 0.1270000000000000 | 2380.696000000000 | 232.174000000000007 | 0.000 | 65 |
| M\_0.7\_F\_pth\_Set2 | 1.721 | 0 | 0.1090000000000000 | 2820.943000000000 | 672.421000000000049 | 0.000 | 85 |
| M\_0.7\_F\_pth\_Set3 | 1.722 | 0 | 0.1450000000000000 | 3452.775000000000 | 1304.252999999999929 | 0.000 | 94 |
| M\_0.7\_F\_lqpt\_Set1 | 1.715 | 0 | 0.1090000000000000 | 2345.006000000000 | 196.484000000000009 | 0.000 | 62 |
| M\_0.7\_F\_lqpt\_Set2 | 1.714 | 0 | 0.1270000000000000 | 2669.201000000000 | 520.677999999999997 | 0.000 | 81 |
| M\_0.7\_F\_lqpt\_Set3 | 1.720 | 0 | 0.1090000000000000 | 3077.791000000000 | 929.269000000000005 | 0.000 | 90 |
| M\_0.7\_F\_lqph\_Set1 | 1.704 | 0 | 0.1090000000000000 | 2206.300000000000 | 57.777999999999999 | 0.000 | 37 |
| M\_0.7\_F\_lqph\_Set2 | 1.700 | 0 | 0.1090000000000000 | 2291.108000000000 | 142.586000000000013 | 0.000 | 53 |
| M\_0.7\_F\_lqph\_Set3 | 1.715 | 0 | 0.1090000000000000 | 2388.820000000000 | 240.296999999999997 | 0.000 | 64 |
| M\_0.7\_F\_lqth\_Set1 | 1.716 | 0 | 0.1450000000000000 | 2327.265000000000 | 178.741999999999990 | 0.000 | 60 |
| M\_0.7\_F\_lqth\_Set2 | 1.722 | 0 | 0.1270000000000000 | 2584.967000000000 | 436.444000000000017 | 0.000 | 78 |
| M\_0.7\_F\_lqth\_Set3 | 1.722 | 0 | 0.1640000000000000 | 3154.881000000000 | 1006.357999999999947 | 0.000 | 91 |
| M\_0.7\_F\_lpth\_Set1 | 1.718 | 0 | 0.1270000000000000 | 2393.375000000000 | 244.852000000000004 | 0.000 | 66 |
| M\_0.7\_F\_lpth\_Set2 | 1.719 | 0 | 0.1090000000000000 | 2918.801000000000 | 770.278000000000020 | 0.000 | 87 |
| M\_0.7\_F\_lpth\_Set3 | 1.725 | 0 | 0.1450000000000000 | 3732.474000000000 | 1583.951000000000022 | 0.000 | 96 |
| M\_0.7\_F\_lqpth\_Set1 | 1.720 | 0 | 0.1090000000000000 | 2407.717000000000 | 259.194000000000017 | 0.000 | 67 |
| M\_0.7\_F\_lqpth\_Set2 | 1.717 | 0 | 0.1090000000000000 | 2779.828000000000 | 631.304999999999950 | 0.000 | 84 |
| M\_0.7\_F\_lqpth\_Set3 | 1.717 | 0 | 0.1450000000000000 | 3582.355000000000 | 1433.833000000000084 | 0.000 | 95 |
| M\_0.8\_F\_l\_Set1 | 1.513 | 0 | 0.0730000000000000 | 2272.327000000000 | 123.804000000000002 | 0.000 | 6 |
| M\_0.8\_F\_l\_Set2 | 1.525 | 0 | 0.0730000000000000 | 2271.870000000000 | 123.347999999999999 | 0.000 | 8 |
| M\_0.8\_F\_l\_Set3 | 1.527 | 0 | 0.0730000000000000 | 2277.011000000000 | 128.488000000000000 | 0.000 | 11 |
| M\_0.8\_F\_q\_Set1 | 1.550 | 0 | 0.0730000000000000 | 2236.837000000000 | 88.313999999999993 | 0.000 | 6 |
| M\_0.8\_F\_q\_Set2 | 1.605 | 0 | 0.0180000000000000 | 2202.225000000000 | 53.703000000000003 | 0.000 | 7 |
| M\_0.8\_F\_q\_Set3 | 1.623 | 0 | 0.0550000000000000 | 2192.106000000000 | 43.584000000000003 | 0.000 | 10 |
| M\_0.8\_F\_p\_Set1 | 1.556 | 0 | 0.0550000000000000 | 2225.907000000000 | 77.385000000000005 | 0.000 | 12 |
| M\_0.8\_F\_p\_Set2 | 1.581 | 0 | 0.0910000000000000 | 2222.817000000000 | 74.293999999999997 | 0.000 | 21 |
| M\_0.8\_F\_p\_Set3 | 1.635 | 0 | 0.0730000000000000 | 2242.661000000000 | 94.138000000000005 | 0.000 | 32 |
| M\_0.8\_F\_t\_Set1 | 1.684 | 0 | 0.1270000000000000 | 2271.658000000000 | 123.135999999999996 | 0.000 | 53 |
| M\_0.8\_F\_t\_Set2 | 1.690 | 0 | 0.1450000000000000 | 2483.080000000000 | 334.557000000000016 | 0.000 | 73 |
| M\_0.8\_F\_t\_Set3 | 1.694 | 0 | 0.1450000000000000 | 2460.773000000000 | 312.250999999999976 | 0.000 | 73 |
| M\_0.8\_F\_h\_Set1 | 1.695 | 0 | 0.0910000000000000 | 2223.494000000000 | 74.971999999999994 | 0.000 | 39 |
| M\_0.8\_F\_h\_Set2 | 1.702 | 0 | 0.1090000000000000 | 2246.197000000000 | 97.674999999999997 | 0.000 | 45 |
| M\_0.8\_F\_h\_Set3 | 1.703 | 0 | 0.1090000000000000 | 2238.883000000000 | 90.361000000000004 | 0.000 | 45 |
| M\_0.8\_F\_lq\_Set1 | 1.659 | 0 | 0.0550000000000000 | 2169.091000000000 | 20.568999999999999 | 0.000 | 11 |
| M\_0.8\_F\_lq\_Set2 | 1.659 | 0 | 0.0550000000000000 | 2174.686000000000 | 26.164000000000001 | 0.000 | 15 |
| M\_0.8\_F\_lq\_Set3 | 1.666 | 0 | 0.0730000000000000 | 2173.660000000000 | 25.138000000000002 | 0.000 | 15 |
| M\_0.8\_F\_lp\_Set1 | 1.604 | 0 | 0.0730000000000000 | 2188.266000000000 | 39.744000000000000 | 0.000 | 14 |
| M\_0.8\_F\_lp\_Set2 | 1.606 | 0 | 0.0910000000000000 | 2227.227000000000 | 78.704999999999998 | 0.000 | 28 |
| M\_0.8\_F\_lp\_Set3 | 1.639 | 0 | 0.0910000000000000 | 2235.157000000000 | 86.634000000000000 | 0.000 | 32 |
| M\_0.8\_F\_lt\_Set1 | 1.711 | 0 | 0.1090000000000000 | 2249.305000000000 | 100.781999999999996 | 0.000 | 50 |
| M\_0.8\_F\_lt\_Set2 | 1.714 | 0 | 0.1270000000000000 | 2450.571000000000 | 302.048999999999978 | 0.000 | 71 |
| M\_0.8\_F\_lt\_Set3 | 1.722 | 0 | 0.0910000000000000 | 2486.030000000000 | 337.507000000000005 | 0.000 | 74 |
| M\_0.8\_F\_lh\_Set1 | 1.698 | 0 | 0.1090000000000000 | 2179.066000000000 | 30.544000000000000 | 0.000 | 29 |
| M\_0.8\_F\_lh\_Set2 | 1.707 | 0 | 0.1090000000000000 | 2227.719000000000 | 79.195999999999998 | 0.000 | 42 |
| M\_0.8\_F\_lh\_Set3 | 1.712 | 0 | 0.1090000000000000 | 2208.426000000000 | 59.904000000000003 | 0.000 | 40 |
| M\_0.8\_F\_qp\_Set1 | 1.619 | 0 | 0.0730000000000000 | 2191.133000000000 | 42.610999999999997 | 0.000 | 15 |
| M\_0.8\_F\_qp\_Set2 | 1.628 | 0 | 0.0730000000000000 | 2209.144000000000 | 60.621000000000002 | 0.000 | 23 |
| M\_0.8\_F\_qp\_Set3 | 1.640 | 0 | 0.0730000000000000 | 2240.969000000000 | 92.445999999999998 | 0.000 | 33 |
| M\_0.8\_F\_qt\_Set1 | 1.717 | 0 | 0.1090000000000000 | 2237.460000000000 | 88.938000000000002 | 0.000 | 48 |
| M\_0.8\_F\_qt\_Set2 | 1.719 | 0 | 0.1270000000000000 | 2325.330000000000 | 176.807999999999993 | 0.000 | 62 |
| M\_0.8\_F\_qt\_Set3 | 1.725 | 0 | 0.0910000000000000 | 2486.972000000000 | 338.449999999999989 | 0.000 | 74 |
| M\_0.8\_F\_qh\_Set1 | 1.703 | 0 | 0.1090000000000000 | 2182.174000000000 | 33.651000000000003 | 0.000 | 30 |
| M\_0.8\_F\_qh\_Set2 | 1.705 | 0 | 0.1090000000000000 | 2234.272000000000 | 85.750000000000000 | 0.000 | 43 |
| M\_0.8\_F\_qh\_Set3 | 1.707 | 0 | 0.1090000000000000 | 2276.985000000000 | 128.462999999999994 | 0.000 | 51 |
| M\_0.8\_F\_pt\_Set1 | 1.694 | 0 | 0.1450000000000000 | 2287.381000000000 | 138.859000000000009 | 0.000 | 55 |
| M\_0.8\_F\_pt\_Set2 | 1.705 | 0 | 0.1450000000000000 | 2390.199000000000 | 241.675999999999988 | 0.000 | 67 |
| M\_0.8\_F\_pt\_Set3 | 1.711 | 0 | 0.0910000000000000 | 2399.402000000000 | 250.879999999999995 | 0.000 | 69 |
| M\_0.8\_F\_ph\_Set1 | 1.696 | 0 | 0.1090000000000000 | 2206.891000000000 | 58.369000000000000 | 0.000 | 37 |
| M\_0.8\_F\_ph\_Set2 | 1.703 | 0 | 0.1090000000000000 | 2253.057000000000 | 104.534000000000006 | 0.000 | 47 |
| M\_0.8\_F\_ph\_Set3 | 1.709 | 0 | 0.1090000000000000 | 2294.319000000000 | 145.795999999999992 | 0.000 | 54 |
| M\_0.8\_F\_th\_Set1 | 1.707 | 0 | 0.1090000000000000 | 2326.597000000000 | 178.074999999999989 | 0.000 | 59 |
| M\_0.8\_F\_th\_Set2 | 1.712 | 0 | 0.1270000000000000 | 2629.328000000000 | 480.805999999999983 | 0.000 | 79 |
| M\_0.8\_F\_th\_Set3 | 1.713 | 0 | 0.1450000000000000 | 2816.040000000000 | 667.517000000000053 | 0.000 | 85 |
| M\_0.8\_F\_lqp\_Set1 | 1.641 | 0 | 0.0730000000000000 | 2189.854000000000 | 41.332000000000001 | 0.000 | 19 |
| M\_0.8\_F\_lqp\_Set2 | 1.645 | 0 | 0.0730000000000000 | 2197.610000000000 | 49.088000000000001 | 0.000 | 23 |
| M\_0.8\_F\_lqp\_Set3 | 1.650 | 0 | 0.0730000000000000 | 2250.379000000000 | 101.856999999999999 | 0.000 | 36 |
| M\_0.8\_F\_lqt\_Set1 | 1.720 | 0 | 0.1090000000000000 | 2258.000000000000 | 109.477999999999994 | 0.000 | 51 |
| M\_0.8\_F\_lqt\_Set2 | 1.718 | 0 | 0.1270000000000000 | 2361.408000000000 | 212.885999999999996 | 0.000 | 65 |
| M\_0.8\_F\_lqt\_Set3 | 1.725 | 0 | 0.0910000000000000 | 2486.456000000000 | 337.934000000000026 | 0.000 | 74 |
| M\_0.8\_F\_lqh\_Set1 | 1.698 | 0 | 0.1090000000000000 | 2208.639000000000 | 60.116999999999997 | 0.000 | 36 |
| M\_0.8\_F\_lqh\_Set2 | 1.699 | 0 | 0.1090000000000000 | 2245.639000000000 | 97.117000000000004 | 0.000 | 45 |
| M\_0.8\_F\_lqh\_Set3 | 1.711 | 0 | 0.1090000000000000 | 2255.337000000000 | 106.813999999999993 | 0.000 | 48 |
| M\_0.8\_F\_lpt\_Set1 | 1.700 | 0 | 0.1450000000000000 | 2304.536000000000 | 156.014000000000010 | 0.000 | 57 |
| M\_0.8\_F\_lpt\_Set2 | 1.711 | 0 | 0.1450000000000000 | 2375.314000000000 | 226.790999999999997 | 0.000 | 66 |
| M\_0.8\_F\_lpt\_Set3 | 1.711 | 0 | 0.1090000000000000 | 2509.548000000000 | 361.024999999999977 | 0.000 | 75 |
| M\_0.8\_F\_lph\_Set1 | 1.696 | 0 | 0.1090000000000000 | 2218.392000000000 | 69.869000000000000 | 0.000 | 39 |
| M\_0.8\_F\_lph\_Set2 | 1.701 | 0 | 0.1090000000000000 | 2253.023000000000 | 104.500000000000000 | 0.000 | 47 |
| M\_0.8\_F\_lph\_Set3 | 1.706 | 0 | 0.1090000000000000 | 2319.403000000000 | 170.881000000000000 | 0.000 | 57 |
| M\_0.8\_F\_qpt\_Set1 | 1.714 | 0 | 0.1090000000000000 | 2262.949000000000 | 114.427000000000007 | 0.000 | 52 |
| M\_0.8\_F\_qpt\_Set2 | 1.717 | 0 | 0.1090000000000000 | 2417.526000000000 | 269.002999999999986 | 0.000 | 69 |
| M\_0.8\_F\_qpt\_Set3 | 1.719 | 0 | 0.0910000000000000 | 2529.546000000000 | 381.024000000000001 | 0.000 | 76 |
| M\_0.8\_F\_qph\_Set1 | 1.700 | 0 | 0.1090000000000000 | 2185.513000000000 | 36.990000000000002 | 0.000 | 32 |
| M\_0.8\_F\_qph\_Set2 | 1.702 | 0 | 0.1090000000000000 | 2253.576000000000 | 105.052999999999997 | 0.000 | 47 |
| M\_0.8\_F\_qph\_Set3 | 1.710 | 0 | 0.1090000000000000 | 2302.764000000000 | 154.241999999999990 | 0.000 | 55 |
| M\_0.8\_F\_qth\_Set1 | 1.713 | 0 | 0.1090000000000000 | 2258.330000000000 | 109.808000000000007 | 0.000 | 51 |
| M\_0.8\_F\_qth\_Set2 | 1.710 | 0 | 0.1270000000000000 | 2404.082000000000 | 255.560000000000002 | 0.000 | 68 |
| M\_0.8\_F\_qth\_Set3 | 1.723 | 0 | 0.1450000000000000 | 2580.591000000000 | 432.069000000000017 | 0.000 | 78 |
| M\_0.8\_F\_pth\_Set1 | 1.708 | 0 | 0.1090000000000000 | 2344.850000000000 | 196.328000000000003 | 0.000 | 61 |
| M\_0.8\_F\_pth\_Set2 | 1.716 | 0 | 0.1090000000000000 | 2575.028000000000 | 426.505999999999972 | 0.000 | 77 |
| M\_0.8\_F\_pth\_Set3 | 1.726 | 0 | 0.1270000000000000 | 2818.967000000000 | 670.445000000000050 | 0.000 | 85 |
| M\_0.8\_F\_lqpt\_Set1 | 1.717 | 0 | 0.1090000000000000 | 2271.589000000000 | 123.066999999999993 | 0.000 | 53 |
| M\_0.8\_F\_lqpt\_Set2 | 1.718 | 0 | 0.1090000000000000 | 2449.970000000000 | 301.447999999999979 | 0.000 | 71 |
| M\_0.8\_F\_lqpt\_Set3 | 1.718 | 0 | 0.0910000000000000 | 2529.546000000000 | 381.024000000000001 | 0.000 | 76 |
| M\_0.8\_F\_lqph\_Set1 | 1.696 | 0 | 0.1090000000000000 | 2199.920000000000 | 51.398000000000003 | 0.000 | 35 |
| M\_0.8\_F\_lqph\_Set2 | 1.702 | 0 | 0.1090000000000000 | 2260.496000000000 | 111.972999999999999 | 0.000 | 48 |
| M\_0.8\_F\_lqph\_Set3 | 1.704 | 0 | 0.1090000000000000 | 2347.504000000000 | 198.981999999999999 | 0.000 | 60 |
| M\_0.8\_F\_lqth\_Set1 | 1.708 | 0 | 0.1090000000000000 | 2258.237000000000 | 109.713999999999999 | 0.000 | 51 |
| M\_0.8\_F\_lqth\_Set2 | 1.713 | 0 | 0.1270000000000000 | 2404.267000000000 | 255.744000000000000 | 0.000 | 68 |
| M\_0.8\_F\_lqth\_Set3 | 1.721 | 0 | 0.1270000000000000 | 2580.431000000000 | 431.908999999999992 | 0.000 | 78 |
| M\_0.8\_F\_lpth\_Set1 | 1.708 | 0 | 0.1090000000000000 | 2345.245000000000 | 196.722000000000008 | 0.000 | 61 |
| M\_0.8\_F\_lpth\_Set2 | 1.718 | 0 | 0.1090000000000000 | 2599.858000000000 | 451.336000000000013 | 0.000 | 78 |
| M\_0.8\_F\_lpth\_Set3 | 1.723 | 0 | 0.1270000000000000 | 2701.213000000000 | 552.691000000000031 | 0.000 | 82 |
| M\_0.8\_F\_lqpth\_Set1 | 1.713 | 0 | 0.1090000000000000 | 2270.658000000000 | 122.135000000000005 | 0.000 | 53 |
| M\_0.8\_F\_lqpth\_Set2 | 1.715 | 0 | 0.1090000000000000 | 2551.266000000000 | 402.744000000000028 | 0.000 | 76 |
| M\_0.8\_F\_lqpth\_Set3 | 1.724 | 0 | 0.1090000000000000 | 2635.906000000000 | 487.384000000000015 | 0.000 | 80 |
| M\_0.9\_F\_l\_Set1 | 1.521 | 0 | 0.0730000000000000 | 2270.096000000000 | 121.573999999999998 | 0.000 | 5 |
| M\_0.9\_F\_l\_Set2 | 1.518 | 0 | 0.0730000000000000 | 2270.060000000000 | 121.537999999999997 | 0.000 | 7 |
| M\_0.9\_F\_l\_Set3 | 1.527 | 0 | 0.0730000000000000 | 2278.147000000000 | 129.623999999999995 | 0.000 | 11 |
| M\_0.9\_F\_q\_Set1 | 1.557 | 0 | 0.0730000000000000 | 2236.911000000000 | 88.388999999999996 | 0.000 | 6 |
| M\_0.9\_F\_q\_Set2 | 1.603 | 0 | 0.0180000000000000 | 2202.333000000000 | 53.811000000000000 | 0.000 | 7 |
| M\_0.9\_F\_q\_Set3 | 1.626 | 0 | 0.0550000000000000 | 2192.233000000000 | 43.710999999999999 | 0.000 | 10 |
| M\_0.9\_F\_p\_Set1 | 1.549 | 0 | 0.0550000000000000 | 2227.430000000000 | 78.908000000000001 | 0.000 | 12 |
| M\_0.9\_F\_p\_Set2 | 1.574 | 0 | 0.0910000000000000 | 2220.850000000000 | 72.328000000000003 | 0.000 | 17 |
| M\_0.9\_F\_p\_Set3 | 1.631 | 0 | 0.0550000000000000 | 2235.688000000000 | 87.165000000000006 | 0.000 | 30 |
| M\_0.9\_F\_t\_Set1 | 1.681 | 0 | 0.1270000000000000 | 2230.360000000000 | 81.837000000000003 | 0.000 | 46 |
| M\_0.9\_F\_t\_Set2 | 1.684 | 0 | 0.1450000000000000 | 2325.702000000000 | 177.180000000000007 | 0.000 | 61 |
| M\_0.9\_F\_t\_Set3 | 1.696 | 0 | 0.1270000000000000 | 2349.952000000000 | 201.430000000000007 | 0.000 | 65 |
| M\_0.9\_F\_h\_Set1 | 1.694 | 0 | 0.0910000000000000 | 2226.288000000000 | 77.766000000000005 | 0.000 | 39 |
| M\_0.9\_F\_h\_Set2 | 1.701 | 0 | 0.0910000000000000 | 2217.641000000000 | 69.119000000000000 | 0.000 | 39 |
| M\_0.9\_F\_h\_Set3 | 1.707 | 0 | 0.0910000000000000 | 2201.470000000000 | 52.948000000000000 | 0.000 | 37 |
| M\_0.9\_F\_lq\_Set1 | 1.660 | 0 | 0.0550000000000000 | 2168.057000000000 | 19.535000000000000 | 0.000 | 10 |
| M\_0.9\_F\_lq\_Set2 | 1.662 | 0 | 0.0550000000000000 | 2174.172000000000 | 25.649999999999999 | 0.000 | 14 |
| M\_0.9\_F\_lq\_Set3 | 1.663 | 0 | 0.0730000000000000 | 2177.166000000000 | 28.643000000000001 | 0.000 | 16 |
| M\_0.9\_F\_lp\_Set1 | 1.598 | 0 | 0.0550000000000000 | 2188.346000000000 | 39.823000000000000 | 0.000 | 14 |
| M\_0.9\_F\_lp\_Set2 | 1.596 | 0 | 0.0910000000000000 | 2208.606000000000 | 60.082999999999998 | 0.000 | 23 |
| M\_0.9\_F\_lp\_Set3 | 1.639 | 0 | 0.0910000000000000 | 2230.626000000000 | 82.103999999999999 | 0.000 | 30 |
| M\_0.9\_F\_lt\_Set1 | 1.713 | 0 | 0.1090000000000000 | 2235.397000000000 | 86.873999999999995 | 0.000 | 47 |
| M\_0.9\_F\_lt\_Set2 | 1.716 | 0 | 0.1270000000000000 | 2278.588000000000 | 130.064999999999998 | 0.000 | 56 |
| M\_0.9\_F\_lt\_Set3 | 1.720 | 0 | 0.0910000000000000 | 2427.197000000000 | 278.673999999999978 | 0.000 | 70 |
| M\_0.9\_F\_lh\_Set1 | 1.701 | 0 | 0.1090000000000000 | 2163.948000000000 | 15.426000000000000 | 0.000 | 24 |
| M\_0.9\_F\_lh\_Set2 | 1.699 | 0 | 0.1090000000000000 | 2222.417000000000 | 73.894999999999996 | 0.000 | 40 |
| M\_0.9\_F\_lh\_Set3 | 1.707 | 0 | 0.1090000000000000 | 2229.955000000000 | 81.432000000000002 | 0.000 | 43 |
| M\_0.9\_F\_qp\_Set1 | 1.622 | 0 | 0.0730000000000000 | 2193.124000000000 | 44.600999999999999 | 0.000 | 15 |
| M\_0.9\_F\_qp\_Set2 | 1.621 | 0 | 0.0730000000000000 | 2205.229000000000 | 56.707000000000001 | 0.000 | 21 |
| M\_0.9\_F\_qp\_Set3 | 1.640 | 0 | 0.0730000000000000 | 2251.703000000000 | 103.180999999999997 | 0.000 | 35 |
| M\_0.9\_F\_qt\_Set1 | 1.716 | 0 | 0.1270000000000000 | 2219.114000000000 | 70.591999999999999 | 0.000 | 44 |
| M\_0.9\_F\_qt\_Set2 | 1.718 | 0 | 0.1270000000000000 | 2277.678000000000 | 129.156000000000006 | 0.000 | 56 |
| M\_0.9\_F\_qt\_Set3 | 1.726 | 0 | 0.0730000000000000 | 2355.762000000000 | 207.239000000000004 | 0.000 | 65 |
| M\_0.9\_F\_qh\_Set1 | 1.696 | 0 | 0.1090000000000000 | 2180.690000000000 | 32.167999999999999 | 0.000 | 29 |
| M\_0.9\_F\_qh\_Set2 | 1.700 | 0 | 0.1090000000000000 | 2189.758000000000 | 41.234999999999999 | 0.000 | 33 |
| M\_0.9\_F\_qh\_Set3 | 1.701 | 0 | 0.1090000000000000 | 2237.175000000000 | 88.653000000000006 | 0.000 | 44 |
| M\_0.9\_F\_pt\_Set1 | 1.696 | 0 | 0.1270000000000000 | 2271.118000000000 | 122.594999999999999 | 0.000 | 52 |
| M\_0.9\_F\_pt\_Set2 | 1.705 | 0 | 0.1450000000000000 | 2296.113000000000 | 147.590000000000003 | 0.000 | 58 |
| M\_0.9\_F\_pt\_Set3 | 1.705 | 0 | 0.0910000000000000 | 2341.660000000000 | 193.137000000000000 | 0.000 | 64 |
| M\_0.9\_F\_ph\_Set1 | 1.691 | 0 | 0.1090000000000000 | 2215.682000000000 | 67.159999999999997 | 0.000 | 38 |
| M\_0.9\_F\_ph\_Set2 | 1.697 | 0 | 0.1090000000000000 | 2216.371000000000 | 67.849000000000004 | 0.000 | 40 |
| M\_0.9\_F\_ph\_Set3 | 1.706 | 0 | 0.1090000000000000 | 2315.342000000000 | 166.819999999999993 | 0.000 | 56 |
| M\_0.9\_F\_th\_Set1 | 1.702 | 0 | 0.1090000000000000 | 2283.096000000000 | 134.574000000000012 | 0.000 | 53 |
| M\_0.9\_F\_th\_Set2 | 1.711 | 0 | 0.1090000000000000 | 2434.237000000000 | 285.714999999999975 | 0.000 | 69 |
| M\_0.9\_F\_th\_Set3 | 1.720 | 0 | 0.1270000000000000 | 2713.684000000000 | 565.162000000000035 | 0.000 | 82 |
| M\_0.9\_F\_lqp\_Set1 | 1.643 | 0 | 0.0730000000000000 | 2186.241000000000 | 37.718000000000004 | 0.000 | 18 |
| M\_0.9\_F\_lqp\_Set2 | 1.649 | 0 | 0.0730000000000000 | 2211.968000000000 | 63.445000000000000 | 0.000 | 27 |
| M\_0.9\_F\_lqp\_Set3 | 1.646 | 0 | 0.0730000000000000 | 2258.449000000000 | 109.926000000000002 | 0.000 | 37 |
| M\_0.9\_F\_lqt\_Set1 | 1.721 | 0 | 0.1270000000000000 | 2213.440000000000 | 64.917000000000002 | 0.000 | 43 |
| M\_0.9\_F\_lqt\_Set2 | 1.720 | 0 | 0.1270000000000000 | 2296.113000000000 | 147.590000000000003 | 0.000 | 58 |
| M\_0.9\_F\_lqt\_Set3 | 1.726 | 0 | 0.0730000000000000 | 2397.377000000000 | 248.854000000000013 | 0.000 | 68 |
| M\_0.9\_F\_lqh\_Set1 | 1.705 | 0 | 0.1090000000000000 | 2194.521000000000 | 45.999000000000002 | 0.000 | 32 |
| M\_0.9\_F\_lqh\_Set2 | 1.705 | 0 | 0.1090000000000000 | 2218.215000000000 | 69.692999999999998 | 0.000 | 39 |
| M\_0.9\_F\_lqh\_Set3 | 1.709 | 0 | 0.1090000000000000 | 2220.501000000000 | 71.978999999999999 | 0.000 | 41 |
| M\_0.9\_F\_lpt\_Set1 | 1.696 | 0 | 0.1270000000000000 | 2295.224000000000 | 146.701999999999998 | 0.000 | 55 |
| M\_0.9\_F\_lpt\_Set2 | 1.704 | 0 | 0.1270000000000000 | 2346.556000000000 | 198.033999999999992 | 0.000 | 63 |
| M\_0.9\_F\_lpt\_Set3 | 1.710 | 0 | 0.0910000000000000 | 2366.918000000000 | 218.395000000000010 | 0.000 | 66 |
| M\_0.9\_F\_lph\_Set1 | 1.694 | 0 | 0.1090000000000000 | 2202.104000000000 | 53.582000000000001 | 0.000 | 35 |
| M\_0.9\_F\_lph\_Set2 | 1.703 | 0 | 0.1090000000000000 | 2237.074000000000 | 88.552000000000007 | 0.000 | 44 |
| M\_0.9\_F\_lph\_Set3 | 1.701 | 0 | 0.1090000000000000 | 2315.539000000000 | 167.015999999999991 | 0.000 | 56 |
| M\_0.9\_F\_qpt\_Set1 | 1.717 | 0 | 0.0910000000000000 | 2235.822000000000 | 87.299999999999997 | 0.000 | 47 |
| M\_0.9\_F\_qpt\_Set2 | 1.718 | 0 | 0.1270000000000000 | 2303.861000000000 | 155.337999999999994 | 0.000 | 59 |
| M\_0.9\_F\_qpt\_Set3 | 1.719 | 0 | 0.0910000000000000 | 2380.068000000000 | 231.544999999999987 | 0.000 | 67 |
| M\_0.9\_F\_qph\_Set1 | 1.697 | 0 | 0.1090000000000000 | 2201.087000000000 | 52.564999999999998 | 0.000 | 35 |
| M\_0.9\_F\_qph\_Set2 | 1.703 | 0 | 0.1090000000000000 | 2227.115000000000 | 78.593000000000004 | 0.000 | 42 |
| M\_0.9\_F\_qph\_Set3 | 1.706 | 0 | 0.1090000000000000 | 2324.149000000000 | 175.627000000000010 | 0.000 | 57 |
| M\_0.9\_F\_qth\_Set1 | 1.712 | 0 | 0.1090000000000000 | 2226.107000000000 | 77.584000000000003 | 0.000 | 45 |
| M\_0.9\_F\_qth\_Set2 | 1.714 | 0 | 0.1090000000000000 | 2288.804000000000 | 140.282000000000011 | 0.000 | 57 |
| M\_0.9\_F\_qth\_Set3 | 1.720 | 0 | 0.1270000000000000 | 2412.804000000000 | 264.281999999999982 | 0.000 | 69 |
| M\_0.9\_F\_pth\_Set1 | 1.705 | 0 | 0.1090000000000000 | 2313.963000000000 | 165.441000000000003 | 0.000 | 57 |
| M\_0.9\_F\_pth\_Set2 | 1.713 | 0 | 0.1090000000000000 | 2385.065000000000 | 236.543000000000006 | 0.000 | 66 |
| M\_0.9\_F\_pth\_Set3 | 1.722 | 0 | 0.1270000000000000 | 2411.704000000000 | 263.182000000000016 | 0.000 | 69 |
| M\_0.9\_F\_lqpt\_Set1 | 1.711 | 0 | 0.1270000000000000 | 2242.712000000000 | 94.189999999999998 | 0.000 | 48 |
| M\_0.9\_F\_lqpt\_Set2 | 1.716 | 0 | 0.1270000000000000 | 2294.149000000000 | 145.627000000000010 | 0.000 | 58 |
| M\_0.9\_F\_lqpt\_Set3 | 1.721 | 0 | 0.0910000000000000 | 2380.068000000000 | 231.544999999999987 | 0.000 | 67 |
| M\_0.9\_F\_lqph\_Set1 | 1.693 | 0 | 0.1090000000000000 | 2206.489000000000 | 57.966999999999999 | 0.000 | 36 |
| M\_0.9\_F\_lqph\_Set2 | 1.701 | 0 | 0.1090000000000000 | 2232.020000000000 | 83.497000000000000 | 0.000 | 43 |
| M\_0.9\_F\_lqph\_Set3 | 1.708 | 0 | 0.1090000000000000 | 2306.583000000000 | 158.060000000000002 | 0.000 | 55 |
| M\_0.9\_F\_lqth\_Set1 | 1.706 | 0 | 0.1090000000000000 | 2232.226000000000 | 83.703000000000003 | 0.000 | 46 |
| M\_0.9\_F\_lqth\_Set2 | 1.711 | 0 | 0.1090000000000000 | 2338.980000000000 | 190.457999999999998 | 0.000 | 62 |
| M\_0.9\_F\_lqth\_Set3 | 1.718 | 0 | 0.1270000000000000 | 2384.662000000000 | 236.139000000000010 | 0.000 | 67 |
| M\_0.9\_F\_lpth\_Set1 | 1.710 | 0 | 0.1090000000000000 | 2288.050000000000 | 139.526999999999987 | 0.000 | 54 |
| M\_0.9\_F\_lpth\_Set2 | 1.717 | 0 | 0.1090000000000000 | 2398.656000000000 | 250.133000000000010 | 0.000 | 67 |
| M\_0.9\_F\_lpth\_Set3 | 1.724 | 0 | 0.1270000000000000 | 2396.288000000000 | 247.765999999999991 | 0.000 | 68 |
| M\_0.9\_F\_lqpth\_Set1 | 1.711 | 0 | 0.1090000000000000 | 2263.942000000000 | 115.420000000000002 | 0.000 | 51 |
| M\_0.9\_F\_lqpth\_Set2 | 1.717 | 0 | 0.1090000000000000 | 2348.426000000000 | 199.902999999999992 | 0.000 | 63 |
| M\_0.9\_F\_lqpth\_Set3 | 1.725 | 0 | 0.1090000000000000 | 2381.921000000000 | 233.399000000000001 | 0.000 | 67 |
| M\_1\_F\_l\_Set1 | 1.512 | 0 | 0.0730000000000000 | 2270.113000000000 | 121.590000000000003 | 0.000 | 5 |
| M\_1\_F\_l\_Set2 | 1.522 | 0 | 0.0730000000000000 | 2270.569000000000 | 122.046999999999997 | 0.000 | 7 |
| M\_1\_F\_l\_Set3 | 1.525 | 0 | 0.0730000000000000 | 2274.606000000000 | 126.084000000000003 | 0.000 | 9 |
| M\_1\_F\_q\_Set1 | 1.557 | 0 | 0.0730000000000000 | 2236.989000000000 | 88.466999999999999 | 0.000 | 6 |
| M\_1\_F\_q\_Set2 | 1.602 | 0 | 0.0180000000000000 | 2202.459000000000 | 53.936999999999998 | 0.000 | 7 |
| M\_1\_F\_q\_Set3 | 1.627 | 0 | 0.0360000000000000 | 2192.285000000000 | 43.762999999999998 | 0.000 | 10 |
| M\_1\_F\_p\_Set1 | 1.549 | 0 | 0.0550000000000000 | 2230.464000000000 | 81.941000000000003 | 0.000 | 12 |
| M\_1\_F\_p\_Set2 | 1.573 | 0 | 0.0910000000000000 | 2225.195000000000 | 76.673000000000002 | 0.000 | 17 |
| M\_1\_F\_p\_Set3 | 1.640 | 0 | 0.0550000000000000 | 2226.846000000000 | 78.322999999999993 | 0.000 | 27 |
| M\_1\_F\_t\_Set1 | 1.685 | 0 | 0.1270000000000000 | 2203.317000000000 | 54.795000000000002 | 0.000 | 40 |
| M\_1\_F\_t\_Set2 | 1.688 | 0 | 0.1450000000000000 | 2250.137000000000 | 101.614000000000004 | 0.000 | 51 |
| M\_1\_F\_t\_Set3 | 1.690 | 0 | 0.1270000000000000 | 2327.955000000000 | 179.432999999999993 | 0.000 | 62 |
| M\_1\_F\_h\_Set1 | 1.694 | 0 | 0.0910000000000000 | 2201.371000000000 | 52.848999999999997 | 0.000 | 33 |
| M\_1\_F\_h\_Set2 | 1.696 | 0 | 0.0910000000000000 | 2220.834000000000 | 72.311000000000007 | 0.000 | 39 |
| M\_1\_F\_h\_Set3 | 1.701 | 0 | 0.0910000000000000 | 2196.109000000000 | 47.587000000000003 | 0.000 | 35 |
| M\_1\_F\_lq\_Set1 | 1.657 | 0 | 0.0550000000000000 | 2168.503000000000 | 19.980000000000000 | 0.000 | 10 |
| M\_1\_F\_lq\_Set2 | 1.654 | 0 | 0.0550000000000000 | 2173.325000000000 | 24.803000000000001 | 0.000 | 13 |
| M\_1\_F\_lq\_Set3 | 1.664 | 0 | 0.0730000000000000 | 2173.321000000000 | 24.798999999999999 | 0.000 | 14 |
| M\_1\_F\_lp\_Set1 | 1.589 | 0 | 0.0550000000000000 | 2189.902000000000 | 41.380000000000003 | 0.000 | 14 |
| M\_1\_F\_lp\_Set2 | 1.593 | 0 | 0.0910000000000000 | 2213.192000000000 | 64.670000000000002 | 0.000 | 24 |
| M\_1\_F\_lp\_Set3 | 1.632 | 0 | 0.0730000000000000 | 2225.641000000000 | 77.119000000000000 | 0.000 | 29 |
| M\_1\_F\_lt\_Set1 | 1.714 | 0 | 0.1090000000000000 | 2212.186000000000 | 63.662999999999997 | 0.000 | 42 |
| M\_1\_F\_lt\_Set2 | 1.719 | 0 | 0.1270000000000000 | 2255.599000000000 | 107.076999999999998 | 0.000 | 52 |
| M\_1\_F\_lt\_Set3 | 1.719 | 0 | 0.0910000000000000 | 2299.867000000000 | 151.343999999999994 | 0.000 | 59 |
| M\_1\_F\_lh\_Set1 | 1.697 | 0 | 0.1090000000000000 | 2172.683000000000 | 24.160000000000000 | 0.000 | 26 |
| M\_1\_F\_lh\_Set2 | 1.697 | 0 | 0.1090000000000000 | 2189.305000000000 | 40.783000000000001 | 0.000 | 32 |
| M\_1\_F\_lh\_Set3 | 1.706 | 0 | 0.1090000000000000 | 2199.748000000000 | 51.225000000000001 | 0.000 | 36 |
| M\_1\_F\_qp\_Set1 | 1.609 | 0 | 0.0730000000000000 | 2190.831000000000 | 42.308000000000000 | 0.000 | 14 |
| M\_1\_F\_qp\_Set2 | 1.616 | 0 | 0.0730000000000000 | 2203.002000000000 | 54.478999999999999 | 0.000 | 21 |
| M\_1\_F\_qp\_Set3 | 1.644 | 0 | 0.0730000000000000 | 2226.192000000000 | 77.670000000000002 | 0.000 | 28 |
| M\_1\_F\_qt\_Set1 | 1.711 | 0 | 0.1090000000000000 | 2197.617000000000 | 49.094999999999999 | 0.000 | 39 |
| M\_1\_F\_qt\_Set2 | 1.720 | 0 | 0.1270000000000000 | 2254.313000000000 | 105.790999999999997 | 0.000 | 52 |
| M\_1\_F\_qt\_Set3 | 1.725 | 0 | 0.0730000000000000 | 2282.103000000000 | 133.580999999999989 | 0.000 | 57 |
| M\_1\_F\_qh\_Set1 | 1.698 | 0 | 0.1090000000000000 | 2186.481000000000 | 37.959000000000003 | 0.000 | 30 |
| M\_1\_F\_qh\_Set2 | 1.701 | 0 | 0.1090000000000000 | 2192.686000000000 | 44.162999999999997 | 0.000 | 33 |
| M\_1\_F\_qh\_Set3 | 1.695 | 0 | 0.1090000000000000 | 2225.646000000000 | 77.123999999999995 | 0.000 | 41 |
| M\_1\_F\_pt\_Set1 | 1.693 | 0 | 0.1090000000000000 | 2256.877000000000 | 108.355000000000004 | 0.000 | 49 |
| M\_1\_F\_pt\_Set2 | 1.706 | 0 | 0.1270000000000000 | 2277.017000000000 | 128.494000000000000 | 0.000 | 55 |
| M\_1\_F\_pt\_Set3 | 1.703 | 0 | 0.0910000000000000 | 2307.521000000000 | 158.997999999999990 | 0.000 | 60 |
| M\_1\_F\_ph\_Set1 | 1.702 | 0 | 0.0910000000000000 | 2200.045000000000 | 51.523000000000003 | 0.000 | 34 |
| M\_1\_F\_ph\_Set2 | 1.699 | 0 | 0.0910000000000000 | 2253.237000000000 | 104.715000000000003 | 0.000 | 46 |
| M\_1\_F\_ph\_Set3 | 1.700 | 0 | 0.1090000000000000 | 2265.687000000000 | 117.165000000000006 | 0.000 | 49 |
| M\_1\_F\_th\_Set1 | 1.703 | 0 | 0.1090000000000000 | 2248.371000000000 | 99.849000000000004 | 0.000 | 47 |
| M\_1\_F\_th\_Set2 | 1.701 | 0 | 0.1090000000000000 | 2343.258000000000 | 194.735999999999990 | 0.000 | 61 |
| M\_1\_F\_th\_Set3 | 1.709 | 0 | 0.1090000000000000 | 2441.480000000000 | 292.956999999999994 | 0.000 | 70 |
| M\_1\_F\_lqp\_Set1 | 1.637 | 0 | 0.0730000000000000 | 2181.401000000000 | 32.878000000000000 | 0.000 | 16 |
| M\_1\_F\_lqp\_Set2 | 1.637 | 0 | 0.0730000000000000 | 2204.786000000000 | 56.264000000000003 | 0.000 | 24 |
| M\_1\_F\_lqp\_Set3 | 1.643 | 0 | 0.0730000000000000 | 2231.830000000000 | 83.307000000000002 | 0.000 | 31 |
| M\_1\_F\_lqt\_Set1 | 1.714 | 0 | 0.1090000000000000 | 2208.189000000000 | 59.667000000000002 | 0.000 | 41 |
| M\_1\_F\_lqt\_Set2 | 1.724 | 0 | 0.1270000000000000 | 2269.685000000000 | 121.162999999999997 | 0.000 | 54 |
| M\_1\_F\_lqt\_Set3 | 1.726 | 0 | 0.0730000000000000 | 2264.670000000000 | 116.147000000000006 | 0.000 | 55 |
| M\_1\_F\_lqh\_Set1 | 1.699 | 0 | 0.1090000000000000 | 2181.111000000000 | 32.588999999999999 | 0.000 | 28 |
| M\_1\_F\_lqh\_Set2 | 1.700 | 0 | 0.1090000000000000 | 2203.083000000000 | 54.560000000000002 | 0.000 | 35 |
| M\_1\_F\_lqh\_Set3 | 1.706 | 0 | 0.1090000000000000 | 2215.059000000000 | 66.536000000000001 | 0.000 | 39 |
| M\_1\_F\_lpt\_Set1 | 1.701 | 0 | 0.1090000000000000 | 2230.671000000000 | 82.147999999999996 | 0.000 | 45 |
| M\_1\_F\_lpt\_Set2 | 1.711 | 0 | 0.1270000000000000 | 2303.003000000000 | 154.480999999999995 | 0.000 | 58 |
| M\_1\_F\_lpt\_Set3 | 1.704 | 0 | 0.0910000000000000 | 2364.024000000000 | 215.502000000000010 | 0.000 | 65 |
| M\_1\_F\_lph\_Set1 | 1.700 | 0 | 0.0910000000000000 | 2214.342000000000 | 65.819999999999993 | 0.000 | 37 |
| M\_1\_F\_lph\_Set2 | 1.703 | 0 | 0.0910000000000000 | 2259.800000000000 | 111.278000000000006 | 0.000 | 47 |
| M\_1\_F\_lph\_Set3 | 1.699 | 0 | 0.1090000000000000 | 2319.216000000000 | 170.693000000000012 | 0.000 | 56 |
| M\_1\_F\_qpt\_Set1 | 1.716 | 0 | 0.1090000000000000 | 2211.285000000000 | 62.762999999999998 | 0.000 | 42 |
| M\_1\_F\_qpt\_Set2 | 1.717 | 0 | 0.1270000000000000 | 2276.174000000000 | 127.650999999999996 | 0.000 | 55 |
| M\_1\_F\_qpt\_Set3 | 1.719 | 0 | 0.0910000000000000 | 2328.610000000000 | 180.087999999999994 | 0.000 | 62 |
| M\_1\_F\_qph\_Set1 | 1.697 | 0 | 0.0910000000000000 | 2190.294000000000 | 41.771999999999998 | 0.000 | 32 |
| M\_1\_F\_qph\_Set2 | 1.697 | 0 | 0.0910000000000000 | 2241.304000000000 | 92.781000000000006 | 0.000 | 44 |
| M\_1\_F\_qph\_Set3 | 1.706 | 0 | 0.1090000000000000 | 2280.687000000000 | 132.164999999999992 | 0.000 | 51 |
| M\_1\_F\_qth\_Set1 | 1.710 | 0 | 0.1090000000000000 | 2186.319000000000 | 37.796999999999997 | 0.000 | 36 |
| M\_1\_F\_qth\_Set2 | 1.711 | 0 | 0.1090000000000000 | 2250.551000000000 | 102.028999999999996 | 0.000 | 51 |
| M\_1\_F\_qth\_Set3 | 1.715 | 0 | 0.1270000000000000 | 2274.636000000000 | 126.114000000000004 | 0.000 | 56 |
| M\_1\_F\_pth\_Set1 | 1.704 | 0 | 0.1090000000000000 | 2258.540000000000 | 110.018000000000001 | 0.000 | 49 |
| M\_1\_F\_pth\_Set2 | 1.710 | 0 | 0.1090000000000000 | 2239.726000000000 | 91.203999999999994 | 0.000 | 50 |
| M\_1\_F\_pth\_Set3 | 1.718 | 0 | 0.1090000000000000 | 2331.047000000000 | 182.524000000000001 | 0.000 | 62 |
| M\_1\_F\_lqpt\_Set1 | 1.711 | 0 | 0.1270000000000000 | 2212.347000000000 | 63.823999999999998 | 0.000 | 42 |
| M\_1\_F\_lqpt\_Set2 | 1.719 | 0 | 0.1270000000000000 | 2284.443000000000 | 135.920999999999992 | 0.000 | 56 |
| M\_1\_F\_lqpt\_Set3 | 1.712 | 0 | 0.0910000000000000 | 2328.173000000000 | 179.650000000000006 | 0.000 | 62 |
| M\_1\_F\_lqph\_Set1 | 1.696 | 0 | 0.0910000000000000 | 2196.852000000000 | 48.329000000000001 | 0.000 | 33 |
| M\_1\_F\_lqph\_Set2 | 1.694 | 0 | 0.0910000000000000 | 2236.207000000000 | 87.683999999999997 | 0.000 | 43 |
| M\_1\_F\_lqph\_Set3 | 1.703 | 0 | 0.1090000000000000 | 2259.340000000000 | 110.817999999999998 | 0.000 | 48 |
| M\_1\_F\_lqth\_Set1 | 1.705 | 0 | 0.1090000000000000 | 2205.864000000000 | 57.341999999999999 | 0.000 | 40 |
| M\_1\_F\_lqth\_Set2 | 1.712 | 0 | 0.1090000000000000 | 2257.853000000000 | 109.329999999999998 | 0.000 | 52 |
| M\_1\_F\_lqth\_Set3 | 1.720 | 0 | 0.1090000000000000 | 2283.896000000000 | 135.372999999999990 | 0.000 | 57 |
| M\_1\_F\_lpth\_Set1 | 1.701 | 0 | 0.1090000000000000 | 2227.152000000000 | 78.629999999999995 | 0.000 | 44 |
| M\_1\_F\_lpth\_Set2 | 1.708 | 0 | 0.1090000000000000 | 2295.025000000000 | 146.502999999999986 | 0.000 | 57 |
| M\_1\_F\_lpth\_Set3 | 1.718 | 0 | 0.1090000000000000 | 2331.503000000000 | 182.980999999999995 | 0.000 | 62 |
| M\_1\_F\_lqpth\_Set1 | 1.705 | 0 | 0.1090000000000000 | 2209.926000000000 | 61.402999999999999 | 0.000 | 41 |
| M\_1\_F\_lqpth\_Set2 | 1.703 | 0 | 0.1090000000000000 | 2314.719000000000 | 166.195999999999998 | 0.000 | 59 |
| M\_1\_F\_lqpth\_Set3 | 1.714 | 0 | 0.1090000000000000 | 2330.364000000000 | 181.841000000000008 | 0.000 | 62 |
| M\_2\_F\_l\_Set1 | 1.504 | 0 | 0.0730000000000000 | 2270.516000000000 | 121.994000000000000 | 0.000 | 5 |
| M\_2\_F\_l\_Set2 | 1.499 | 0 | 0.0730000000000000 | 2272.019000000000 | 123.497000000000000 | 0.000 | 6 |
| M\_2\_F\_l\_Set3 | 1.506 | 0 | 0.0730000000000000 | 2274.360000000000 | 125.837999999999994 | 0.000 | 7 |
| M\_2\_F\_q\_Set1 | 1.551 | 0 | 0.0910000000000000 | 2238.106000000000 | 89.582999999999998 | 0.000 | 6 |
| M\_2\_F\_q\_Set2 | 1.587 | 0 | 0.0180000000000000 | 2204.355000000000 | 55.832999999999998 | 0.000 | 7 |
| M\_2\_F\_q\_Set3 | 1.598 | 0 | 0.0550000000000000 | 2192.442000000000 | 43.918999999999997 | 0.000 | 9 |
| M\_2\_F\_p\_Set1 | 1.532 | 0 | 0.0730000000000000 | 2243.056000000000 | 94.533000000000001 | 0.000 | 11 |
| M\_2\_F\_p\_Set2 | 1.542 | 0 | 0.0910000000000000 | 2233.380000000000 | 84.858000000000004 | 0.000 | 17 |
| M\_2\_F\_p\_Set3 | 1.615 | 0 | 0.0550000000000000 | 2215.230000000000 | 66.707999999999998 | 0.000 | 21 |
| M\_2\_F\_t\_Set1 | 1.617 | 0 | 0.1090000000000000 | 2173.899000000000 | 25.376999999999999 | 0.000 | 25 |
| M\_2\_F\_t\_Set2 | 1.622 | 0 | 0.1090000000000000 | 2166.612000000000 | 18.090000000000000 | 0.000 | 24 |
| M\_2\_F\_t\_Set3 | 1.638 | 0 | 0.0910000000000000 | 2181.198000000000 | 32.674999999999997 | 0.000 | 29 |
| M\_2\_F\_h\_Set1 | 1.686 | 0 | 0.0910000000000000 | 2188.001000000000 | 39.478000000000002 | 0.000 | 25 |
| M\_2\_F\_h\_Set2 | 1.692 | 0 | 0.0910000000000000 | 2196.649000000000 | 48.127000000000002 | 0.000 | 28 |
| M\_2\_F\_h\_Set3 | 1.697 | 0 | 0.0910000000000000 | 2182.798000000000 | 34.274999999999999 | 0.000 | 25 |
| M\_2\_F\_lq\_Set1 | 1.612 | 0 | 0.0550000000000000 | 2178.138000000000 | 29.616000000000000 | 0.000 | 9 |
| M\_2\_F\_lq\_Set2 | 1.623 | 0 | 0.0550000000000000 | 2183.906000000000 | 35.384000000000000 | 0.000 | 11 |
| M\_2\_F\_lq\_Set3 | 1.636 | 0 | 0.0550000000000000 | 2184.450000000000 | 35.927999999999997 | 0.000 | 12 |
| M\_2\_F\_lp\_Set1 | 1.547 | 0 | 0.0550000000000000 | 2189.031000000000 | 40.509000000000000 | 0.000 | 10 |
| M\_2\_F\_lp\_Set2 | 1.554 | 0 | 0.0730000000000000 | 2202.824000000000 | 54.301000000000002 | 0.000 | 16 |
| M\_2\_F\_lp\_Set3 | 1.612 | 0 | 0.0550000000000000 | 2202.485000000000 | 53.962000000000003 | 0.000 | 19 |
| M\_2\_F\_lt\_Set1 | 1.672 | 0 | 0.1090000000000000 | 2163.595000000000 | 15.073000000000000 | 0.000 | 22 |
| M\_2\_F\_lt\_Set2 | 1.675 | 0 | 0.1090000000000000 | 2180.540000000000 | 32.017000000000003 | 0.000 | 29 |
| M\_2\_F\_lt\_Set3 | 1.689 | 0 | 0.0910000000000000 | 2195.305000000000 | 46.783000000000001 | 0.000 | 34 |
| M\_2\_F\_lh\_Set1 | 1.698 | 0 | 0.0730000000000000 | 2166.517000000000 | 17.995000000000001 | 0.000 | 19 |
| M\_2\_F\_lh\_Set2 | 1.695 | 0 | 0.0730000000000000 | 2160.988000000000 | 12.465999999999999 | 0.000 | 18 |
| M\_2\_F\_lh\_Set3 | 1.699 | 0 | 0.0730000000000000 | 2162.908000000000 | 14.385000000000000 | 0.000 | 20 |
| M\_2\_F\_qp\_Set1 | 1.567 | 0 | 0.0910000000000000 | 2194.986000000000 | 46.463999999999999 | 0.000 | 13 |
| M\_2\_F\_qp\_Set2 | 1.561 | 0 | 0.0910000000000000 | 2204.720000000000 | 56.198000000000000 | 0.000 | 18 |
| M\_2\_F\_qp\_Set3 | 1.633 | 0 | 0.0550000000000000 | 2208.862000000000 | 60.340000000000003 | 0.000 | 21 |
| M\_2\_F\_qt\_Set1 | 1.679 | 0 | 0.0910000000000000 | 2179.656000000000 | 31.134000000000000 | 0.000 | 28 |
| M\_2\_F\_qt\_Set2 | 1.686 | 0 | 0.1270000000000000 | 2174.501000000000 | 25.978999999999999 | 0.000 | 28 |
| M\_2\_F\_qt\_Set3 | 1.695 | 0 | 0.1090000000000000 | 2176.813000000000 | 28.289999999999999 | 0.000 | 30 |
| M\_2\_F\_qh\_Set1 | 1.698 | 0 | 0.0910000000000000 | 2157.036000000000 | 8.513000000000000 | 0.000 | 15 |
| M\_2\_F\_qh\_Set2 | 1.696 | 0 | 0.0910000000000000 | 2154.918000000000 | 6.395000000000000 | 0.000 | 15 |
| M\_2\_F\_qh\_Set3 | 1.696 | 0 | 0.0910000000000000 | 2160.338000000000 | 11.815000000000000 | 0.000 | 18 |
| M\_2\_F\_pt\_Set1 | 1.656 | 0 | 0.1090000000000000 | 2167.693000000000 | 19.170999999999999 | 0.000 | 23 |
| M\_2\_F\_pt\_Set2 | 1.654 | 0 | 0.1090000000000000 | 2183.027000000000 | 34.505000000000003 | 0.000 | 30 |
| M\_2\_F\_pt\_Set3 | 1.678 | 0 | 0.1270000000000000 | 2210.249000000000 | 61.726999999999997 | 0.000 | 37 |
| M\_2\_F\_ph\_Set1 | 1.687 | 0 | 0.0910000000000000 | 2177.190000000000 | 28.667999999999999 | 0.000 | 21 |
| M\_2\_F\_ph\_Set2 | 1.698 | 0 | 0.0910000000000000 | 2169.402000000000 | 20.879000000000001 | 0.000 | 21 |
| M\_2\_F\_ph\_Set3 | 1.689 | 0 | 0.0910000000000000 | 2194.721000000000 | 46.198999999999998 | 0.000 | 29 |
| M\_2\_F\_th\_Set1 | 1.696 | 0 | 0.0910000000000000 | 2182.737000000000 | 34.215000000000003 | 0.000 | 27 |
| M\_2\_F\_th\_Set2 | 1.699 | 0 | 0.0910000000000000 | 2195.041000000000 | 46.518000000000001 | 0.000 | 31 |
| M\_2\_F\_th\_Set3 | 1.684 | 0 | 0.0910000000000000 | 2183.720000000000 | 35.198000000000000 | 0.000 | 30 |
| M\_2\_F\_lqp\_Set1 | 1.566 | 0 | 0.0550000000000000 | 2187.212000000000 | 38.689000000000000 | 0.000 | 13 |
| M\_2\_F\_lqp\_Set2 | 1.574 | 0 | 0.0550000000000000 | 2191.128000000000 | 42.606000000000002 | 0.000 | 16 |
| M\_2\_F\_lqp\_Set3 | 1.632 | 0 | 0.0550000000000000 | 2202.367000000000 | 53.844999999999999 | 0.000 | 20 |
| M\_2\_F\_lqt\_Set1 | 1.688 | 0 | 0.1090000000000000 | 2168.104000000000 | 19.582000000000001 | 0.000 | 24 |
| M\_2\_F\_lqt\_Set2 | 1.692 | 0 | 0.1090000000000000 | 2187.731000000000 | 39.209000000000003 | 0.000 | 31 |
| M\_2\_F\_lqt\_Set3 | 1.697 | 0 | 0.0910000000000000 | 2184.682000000000 | 36.159999999999997 | 0.000 | 32 |
| M\_2\_F\_lqh\_Set1 | 1.700 | 0 | 0.0910000000000000 | 2159.745000000000 | 11.223000000000001 | 0.000 | 16 |
| M\_2\_F\_lqh\_Set2 | 1.694 | 0 | 0.0910000000000000 | 2149.646000000000 | 1.123000000000000 | 0.001 | 13 |
| M\_2\_F\_lqh\_Set3 | 1.698 | 0 | 0.0910000000000000 | 2166.186000000000 | 17.664000000000001 | 0.000 | 20 |
| M\_2\_F\_lpt\_Set1 | 1.654 | 0 | 0.1090000000000000 | 2169.606000000000 | 21.084000000000000 | 0.000 | 24 |
| M\_2\_F\_lpt\_Set2 | 1.660 | 0 | 0.1090000000000000 | 2181.924000000000 | 33.402000000000001 | 0.000 | 29 |
| M\_2\_F\_lpt\_Set3 | 1.674 | 0 | 0.1090000000000000 | 2206.640000000000 | 58.116999999999997 | 0.000 | 36 |
| M\_2\_F\_lph\_Set1 | 1.693 | 0 | 0.0910000000000000 | 2172.446000000000 | 23.922999999999998 | 0.000 | 20 |
| M\_2\_F\_lph\_Set2 | 1.694 | 0 | 0.0910000000000000 | 2182.889000000000 | 34.366999999999997 | 0.000 | 25 |
| M\_2\_F\_lph\_Set3 | 1.685 | 0 | 0.0910000000000000 | 2180.330000000000 | 31.806999999999999 | 0.000 | 25 |
| M\_2\_F\_qpt\_Set1 | 1.673 | 0 | 0.1090000000000000 | 2179.294000000000 | 30.771999999999998 | 0.000 | 27 |
| M\_2\_F\_qpt\_Set2 | 1.677 | 0 | 0.1090000000000000 | 2207.974000000000 | 59.451999999999998 | 0.000 | 36 |
| M\_2\_F\_qpt\_Set3 | 1.691 | 0 | 0.0910000000000000 | 2211.503000000000 | 62.981000000000002 | 0.000 | 37 |
| M\_2\_F\_qph\_Set1 | 1.694 | 0 | 0.0910000000000000 | 2178.137000000000 | 29.614999999999998 | 0.000 | 22 |
| M\_2\_F\_qph\_Set2 | 1.698 | 0 | 0.0910000000000000 | 2167.926000000000 | 19.404000000000000 | 0.000 | 21 |
| M\_2\_F\_qph\_Set3 | 1.687 | 0 | 0.0910000000000000 | 2199.495000000000 | 50.972000000000001 | 0.000 | 30 |
| M\_2\_F\_qth\_Set1 | 1.700 | 0 | 0.0910000000000000 | 2165.250000000000 | 16.728000000000002 | 0.000 | 23 |
| M\_2\_F\_qth\_Set2 | 1.701 | 0 | 0.0910000000000000 | 2173.009000000000 | 24.486999999999998 | 0.000 | 27 |
| M\_2\_F\_qth\_Set3 | 1.700 | 0 | 0.0910000000000000 | 2191.155000000000 | 42.633000000000003 | 0.000 | 33 |
| M\_2\_F\_pth\_Set1 | 1.698 | 0 | 0.0910000000000000 | 2179.492000000000 | 30.969999999999999 | 0.000 | 26 |
| M\_2\_F\_pth\_Set2 | 1.695 | 0 | 0.0910000000000000 | 2203.565000000000 | 55.042999999999999 | 0.000 | 35 |
| M\_2\_F\_pth\_Set3 | 1.690 | 0 | 0.0910000000000000 | 2192.870000000000 | 44.347999999999999 | 0.000 | 34 |
| M\_2\_F\_lqpt\_Set1 | 1.677 | 0 | 0.1090000000000000 | 2183.961000000000 | 35.439000000000000 | 0.000 | 28 |
| M\_2\_F\_lqpt\_Set2 | 1.679 | 0 | 0.1090000000000000 | 2204.079000000000 | 55.557000000000002 | 0.000 | 35 |
| M\_2\_F\_lqpt\_Set3 | 1.691 | 0 | 0.0910000000000000 | 2211.491000000000 | 62.969000000000001 | 0.000 | 37 |
| M\_2\_F\_lqph\_Set1 | 1.694 | 0 | 0.0910000000000000 | 2174.770000000000 | 26.248000000000001 | 0.000 | 21 |
| M\_2\_F\_lqph\_Set2 | 1.697 | 0 | 0.0910000000000000 | 2164.717000000000 | 16.195000000000000 | 0.000 | 19 |
| M\_2\_F\_lqph\_Set3 | 1.690 | 0 | 0.0910000000000000 | 2191.924000000000 | 43.401000000000003 | 0.000 | 28 |
| M\_2\_F\_lqth\_Set1 | 1.706 | 0 | 0.0910000000000000 | 2148.522000000000 | 0.000000000000000 | 0.002 | 17 |
| M\_2\_F\_lqth\_Set2 | 1.701 | 0 | 0.0910000000000000 | 2166.744000000000 | 18.222000000000001 | 0.000 | 25 |
| M\_2\_F\_lqth\_Set3 | 1.705 | 0 | 0.0910000000000000 | 2174.639000000000 | 26.117000000000001 | 0.000 | 29 |
| M\_2\_F\_lpth\_Set1 | 1.697 | 0 | 0.0910000000000000 | 2184.333000000000 | 35.810000000000002 | 0.000 | 28 |
| M\_2\_F\_lpth\_Set2 | 1.693 | 0 | 0.0910000000000000 | 2207.777000000000 | 59.255000000000003 | 0.000 | 36 |
| M\_2\_F\_lpth\_Set3 | 1.695 | 0 | 0.0910000000000000 | 2211.436000000000 | 62.914000000000001 | 0.000 | 38 |
| M\_2\_F\_lqpth\_Set1 | 1.699 | 0 | 0.0910000000000000 | 2154.724000000000 | 6.202000000000000 | 0.000 | 19 |
| M\_2\_F\_lqpth\_Set2 | 1.696 | 0 | 0.0910000000000000 | 2186.578000000000 | 38.055000000000000 | 0.000 | 31 |
| M\_2\_F\_lqpth\_Set3 | 1.692 | 0 | 0.0910000000000000 | 2206.999000000000 | 58.476999999999997 | 0.000 | 37 |
| M\_3\_F\_l\_Set1 | 1.480 | 0 | 0.0910000000000000 | 2271.416000000000 | 122.893000000000001 | 0.000 | 5 |
| M\_3\_F\_l\_Set2 | 1.478 | 0 | 0.0910000000000000 | 2273.048000000000 | 124.525999999999996 | 0.000 | 6 |
| M\_3\_F\_l\_Set3 | 1.479 | 0 | 0.1090000000000000 | 2275.605000000000 | 127.082999999999998 | 0.000 | 7 |
| M\_3\_F\_q\_Set1 | 1.547 | 0 | 0.0730000000000000 | 2239.798000000000 | 91.275000000000006 | 0.000 | 6 |
| M\_3\_F\_q\_Set2 | 1.579 | 0 | 0.0360000000000000 | 2207.391000000000 | 58.868000000000002 | 0.000 | 7 |
| M\_3\_F\_q\_Set3 | 1.568 | 0 | 0.0360000000000000 | 2193.702000000000 | 45.180000000000000 | 0.000 | 8 |
| M\_3\_F\_p\_Set1 | 1.509 | 0 | 0.0730000000000000 | 2241.479000000000 | 92.956000000000003 | 0.000 | 7 |
| M\_3\_F\_p\_Set2 | 1.501 | 0 | 0.0910000000000000 | 2246.163000000000 | 97.641000000000005 | 0.000 | 16 |
| M\_3\_F\_p\_Set3 | 1.563 | 0 | 0.0550000000000000 | 2206.121000000000 | 57.598999999999997 | 0.000 | 16 |
| M\_3\_F\_t\_Set1 | 1.564 | 0 | 0.0910000000000000 | 2180.364000000000 | 31.841999999999999 | 0.000 | 15 |
| M\_3\_F\_t\_Set2 | 1.567 | 0 | 0.0910000000000000 | 2191.403000000000 | 42.881000000000000 | 0.000 | 19 |
| M\_3\_F\_t\_Set3 | 1.606 | 0 | 0.0910000000000000 | 2186.788000000000 | 38.265000000000001 | 0.000 | 20 |
| M\_3\_F\_h\_Set1 | 1.688 | 0 | 0.0730000000000000 | 2177.460000000000 | 28.937000000000001 | 0.000 | 18 |
| M\_3\_F\_h\_Set2 | 1.681 | 0 | 0.0730000000000000 | 2177.126000000000 | 28.603999999999999 | 0.000 | 18 |
| M\_3\_F\_h\_Set3 | 1.680 | 0 | 0.0730000000000000 | 2180.095000000000 | 31.571999999999999 | 0.000 | 19 |
| M\_3\_F\_lq\_Set1 | 1.606 | 0 | 0.0550000000000000 | 2192.838000000000 | 44.316000000000003 | 0.000 | 9 |
| M\_3\_F\_lq\_Set2 | 1.607 | 0 | 0.0550000000000000 | 2190.983000000000 | 42.460999999999999 | 0.000 | 9 |
| M\_3\_F\_lq\_Set3 | 1.607 | 0 | 0.0550000000000000 | 2186.740000000000 | 38.216999999999999 | 0.000 | 11 |
| M\_3\_F\_lp\_Set1 | 1.523 | 0 | 0.0550000000000000 | 2200.537000000000 | 52.015000000000001 | 0.000 | 9 |
| M\_3\_F\_lp\_Set2 | 1.515 | 0 | 0.0730000000000000 | 2212.665000000000 | 64.143000000000001 | 0.000 | 15 |
| M\_3\_F\_lp\_Set3 | 1.590 | 0 | 0.0550000000000000 | 2194.013000000000 | 45.490000000000002 | 0.000 | 13 |
| M\_3\_F\_lt\_Set1 | 1.589 | 0 | 0.0910000000000000 | 2170.214000000000 | 21.692000000000000 | 0.000 | 17 |
| M\_3\_F\_lt\_Set2 | 1.595 | 0 | 0.0910000000000000 | 2178.506000000000 | 29.983000000000001 | 0.000 | 21 |
| M\_3\_F\_lt\_Set3 | 1.610 | 0 | 0.1090000000000000 | 2183.682000000000 | 35.158999999999999 | 0.000 | 24 |
| M\_3\_F\_lh\_Set1 | 1.683 | 0 | 0.0730000000000000 | 2159.500000000000 | 10.977000000000000 | 0.000 | 13 |
| M\_3\_F\_lh\_Set2 | 1.686 | 0 | 0.0730000000000000 | 2173.024000000000 | 24.501000000000001 | 0.000 | 18 |
| M\_3\_F\_lh\_Set3 | 1.686 | 0 | 0.0730000000000000 | 2169.777000000000 | 21.254000000000001 | 0.000 | 17 |
| M\_3\_F\_qp\_Set1 | 1.534 | 0 | 0.0910000000000000 | 2200.769000000000 | 52.247000000000000 | 0.000 | 12 |
| M\_3\_F\_qp\_Set2 | 1.534 | 0 | 0.0910000000000000 | 2208.797000000000 | 60.274999999999999 | 0.000 | 17 |
| M\_3\_F\_qp\_Set3 | 1.610 | 0 | 0.0550000000000000 | 2190.639000000000 | 42.116999999999997 | 0.000 | 13 |
| M\_3\_F\_qt\_Set1 | 1.625 | 0 | 0.0910000000000000 | 2170.672000000000 | 22.149999999999999 | 0.000 | 17 |
| M\_3\_F\_qt\_Set2 | 1.628 | 0 | 0.0730000000000000 | 2173.155000000000 | 24.632000000000001 | 0.000 | 20 |
| M\_3\_F\_qt\_Set3 | 1.649 | 0 | 0.0730000000000000 | 2180.093000000000 | 31.570000000000000 | 0.000 | 23 |
| M\_3\_F\_qh\_Set1 | 1.685 | 0 | 0.0730000000000000 | 2165.158000000000 | 16.635999999999999 | 0.000 | 15 |
| M\_3\_F\_qh\_Set2 | 1.685 | 0 | 0.0910000000000000 | 2161.389000000000 | 12.866000000000000 | 0.000 | 14 |
| M\_3\_F\_qh\_Set3 | 1.686 | 0 | 0.0730000000000000 | 2166.997000000000 | 18.474000000000000 | 0.000 | 16 |
| M\_3\_F\_pt\_Set1 | 1.567 | 0 | 0.1270000000000000 | 2166.122000000000 | 17.600000000000001 | 0.000 | 16 |
| M\_3\_F\_pt\_Set2 | 1.572 | 0 | 0.1270000000000000 | 2185.313000000000 | 36.790999999999997 | 0.000 | 23 |
| M\_3\_F\_pt\_Set3 | 1.611 | 0 | 0.1090000000000000 | 2195.308000000000 | 46.784999999999997 | 0.000 | 26 |
| M\_3\_F\_ph\_Set1 | 1.684 | 0 | 0.0730000000000000 | 2164.354000000000 | 15.832000000000001 | 0.000 | 13 |
| M\_3\_F\_ph\_Set2 | 1.685 | 0 | 0.0730000000000000 | 2169.250000000000 | 20.728000000000002 | 0.000 | 16 |
| M\_3\_F\_ph\_Set3 | 1.687 | 0 | 0.0730000000000000 | 2176.114000000000 | 27.591999999999999 | 0.000 | 18 |
| M\_3\_F\_th\_Set1 | 1.690 | 0 | 0.0730000000000000 | 2166.725000000000 | 18.202000000000002 | 0.000 | 17 |
| M\_3\_F\_th\_Set2 | 1.686 | 0 | 0.0730000000000000 | 2178.998000000000 | 30.475999999999999 | 0.000 | 21 |
| M\_3\_F\_th\_Set3 | 1.691 | 0 | 0.0730000000000000 | 2185.006000000000 | 36.482999999999997 | 0.000 | 24 |
| M\_3\_F\_lqp\_Set1 | 1.537 | 0 | 0.0730000000000000 | 2188.821000000000 | 40.298000000000002 | 0.000 | 11 |
| M\_3\_F\_lqp\_Set2 | 1.540 | 0 | 0.0730000000000000 | 2190.519000000000 | 41.997000000000000 | 0.000 | 13 |
| M\_3\_F\_lqp\_Set3 | 1.601 | 0 | 0.0550000000000000 | 2187.547000000000 | 39.024000000000001 | 0.000 | 12 |
| M\_3\_F\_lqt\_Set1 | 1.626 | 0 | 0.0550000000000000 | 2169.092000000000 | 20.568999999999999 | 0.000 | 17 |
| M\_3\_F\_lqt\_Set2 | 1.622 | 0 | 0.0550000000000000 | 2176.017000000000 | 27.495000000000001 | 0.000 | 20 |
| M\_3\_F\_lqt\_Set3 | 1.646 | 0 | 0.0730000000000000 | 2181.759000000000 | 33.235999999999997 | 0.000 | 23 |
| M\_3\_F\_lqh\_Set1 | 1.691 | 0 | 0.0730000000000000 | 2165.551000000000 | 17.027999999999999 | 0.000 | 15 |
| M\_3\_F\_lqh\_Set2 | 1.695 | 0 | 0.0730000000000000 | 2164.133000000000 | 15.611000000000001 | 0.000 | 15 |
| M\_3\_F\_lqh\_Set3 | 1.692 | 0 | 0.0730000000000000 | 2175.795000000000 | 27.273000000000000 | 0.000 | 19 |
| M\_3\_F\_lpt\_Set1 | 1.589 | 0 | 0.1270000000000000 | 2168.015000000000 | 19.492999999999999 | 0.000 | 17 |
| M\_3\_F\_lpt\_Set2 | 1.585 | 0 | 0.1270000000000000 | 2172.795000000000 | 24.273000000000000 | 0.000 | 19 |
| M\_3\_F\_lpt\_Set3 | 1.616 | 0 | 0.1090000000000000 | 2185.879000000000 | 37.356999999999999 | 0.000 | 23 |
| M\_3\_F\_lph\_Set1 | 1.682 | 0 | 0.0730000000000000 | 2169.511000000000 | 20.989000000000001 | 0.000 | 16 |
| M\_3\_F\_lph\_Set2 | 1.687 | 0 | 0.0730000000000000 | 2174.104000000000 | 25.581000000000000 | 0.000 | 18 |
| M\_3\_F\_lph\_Set3 | 1.686 | 0 | 0.0730000000000000 | 2173.023000000000 | 24.500000000000000 | 0.000 | 17 |
| M\_3\_F\_qpt\_Set1 | 1.583 | 0 | 0.1090000000000000 | 2175.974000000000 | 27.451000000000001 | 0.000 | 19 |
| M\_3\_F\_qpt\_Set2 | 1.587 | 0 | 0.1090000000000000 | 2183.467000000000 | 34.945000000000000 | 0.000 | 22 |
| M\_3\_F\_qpt\_Set3 | 1.622 | 0 | 0.1090000000000000 | 2187.737000000000 | 39.213999999999999 | 0.000 | 23 |
| M\_3\_F\_qph\_Set1 | 1.689 | 0 | 0.0730000000000000 | 2178.837000000000 | 30.314000000000000 | 0.000 | 19 |
| M\_3\_F\_qph\_Set2 | 1.689 | 0 | 0.0730000000000000 | 2177.592000000000 | 29.070000000000000 | 0.000 | 20 |
| M\_3\_F\_qph\_Set3 | 1.687 | 0 | 0.0730000000000000 | 2180.902000000000 | 32.380000000000003 | 0.000 | 20 |
| M\_3\_F\_qth\_Set1 | 1.693 | 0 | 0.0910000000000000 | 2157.236000000000 | 8.712999999999999 | 0.000 | 15 |
| M\_3\_F\_qth\_Set2 | 1.688 | 0 | 0.0910000000000000 | 2157.968000000000 | 9.445000000000000 | 0.000 | 16 |
| M\_3\_F\_qth\_Set3 | 1.693 | 0 | 0.0730000000000000 | 2160.850000000000 | 12.327999999999999 | 0.000 | 18 |
| M\_3\_F\_pth\_Set1 | 1.688 | 0 | 0.0730000000000000 | 2169.572000000000 | 21.048999999999999 | 0.000 | 18 |
| M\_3\_F\_pth\_Set2 | 1.689 | 0 | 0.0730000000000000 | 2172.984000000000 | 24.460999999999999 | 0.000 | 20 |
| M\_3\_F\_pth\_Set3 | 1.689 | 0 | 0.0730000000000000 | 2177.033000000000 | 28.510999999999999 | 0.000 | 22 |
| M\_3\_F\_lqpt\_Set1 | 1.585 | 0 | 0.1090000000000000 | 2174.807000000000 | 26.285000000000000 | 0.000 | 18 |
| M\_3\_F\_lqpt\_Set2 | 1.587 | 0 | 0.1090000000000000 | 2182.679000000000 | 34.156999999999996 | 0.000 | 21 |
| M\_3\_F\_lqpt\_Set3 | 1.627 | 0 | 0.1090000000000000 | 2188.040000000000 | 39.518000000000001 | 0.000 | 23 |
| M\_3\_F\_lqph\_Set1 | 1.686 | 0 | 0.0730000000000000 | 2168.513000000000 | 19.991000000000000 | 0.000 | 16 |
| M\_3\_F\_lqph\_Set2 | 1.686 | 0 | 0.0730000000000000 | 2178.862000000000 | 30.338999999999999 | 0.000 | 20 |
| M\_3\_F\_lqph\_Set3 | 1.685 | 0 | 0.0730000000000000 | 2175.045000000000 | 26.523000000000000 | 0.000 | 18 |
| M\_3\_F\_lqth\_Set1 | 1.696 | 0 | 0.0730000000000000 | 2157.672000000000 | 9.148999999999999 | 0.000 | 15 |
| M\_3\_F\_lqth\_Set2 | 1.694 | 0 | 0.0730000000000000 | 2161.404000000000 | 12.882000000000000 | 0.000 | 17 |
| M\_3\_F\_lqth\_Set3 | 1.692 | 0 | 0.0550000000000000 | 2169.750000000000 | 21.227000000000000 | 0.000 | 21 |
| M\_3\_F\_lpth\_Set1 | 1.686 | 0 | 0.0730000000000000 | 2171.013000000000 | 22.491000000000000 | 0.000 | 19 |
| M\_3\_F\_lpth\_Set2 | 1.693 | 0 | 0.0730000000000000 | 2179.277000000000 | 30.754999999999999 | 0.000 | 22 |
| M\_3\_F\_lpth\_Set3 | 1.680 | 0 | 0.0730000000000000 | 2168.088000000000 | 19.565000000000001 | 0.000 | 19 |
| M\_3\_F\_lqpth\_Set1 | 1.688 | 0 | 0.0730000000000000 | 2166.125000000000 | 17.603000000000002 | 0.000 | 18 |
| M\_3\_F\_lqpth\_Set2 | 1.689 | 0 | 0.0730000000000000 | 2179.775000000000 | 31.251999999999999 | 0.000 | 23 |
| M\_3\_F\_lqpth\_Set3 | 1.689 | 0 | 0.0730000000000000 | 2180.106000000000 | 31.582999999999998 | 0.000 | 23 |
| M\_4\_F\_l\_Set1 | 1.479 | 0 | 0.0910000000000000 | 2275.112000000000 | 126.590000000000003 | 0.000 | 6 |
| M\_4\_F\_l\_Set2 | 1.482 | 0 | 0.0910000000000000 | 2274.832000000000 | 126.308999999999997 | 0.000 | 6 |
| M\_4\_F\_l\_Set3 | 1.490 | 0 | 0.1090000000000000 | 2275.849000000000 | 127.325999999999993 | 0.000 | 6 |
| M\_4\_F\_q\_Set1 | 1.528 | 0 | 0.0730000000000000 | 2241.991000000000 | 93.468000000000004 | 0.000 | 6 |
| M\_4\_F\_q\_Set2 | 1.530 | 0 | 0.0730000000000000 | 2211.479000000000 | 62.957000000000001 | 0.000 | 7 |
| M\_4\_F\_q\_Set3 | 1.546 | 0 | 0.0550000000000000 | 2198.566000000000 | 50.042999999999999 | 0.000 | 8 |
| M\_4\_F\_p\_Set1 | 1.481 | 0 | 0.0910000000000000 | 2247.591000000000 | 99.069000000000003 | 0.000 | 8 |
| M\_4\_F\_p\_Set2 | 1.463 | 0 | 0.0910000000000000 | 2244.257000000000 | 95.733999999999995 | 0.000 | 12 |
| M\_4\_F\_p\_Set3 | 1.511 | 0 | 0.0550000000000000 | 2201.141000000000 | 52.619000000000000 | 0.000 | 12 |
| M\_4\_F\_t\_Set1 | 1.559 | 0 | 0.1090000000000000 | 2201.938000000000 | 53.415999999999997 | 0.000 | 14 |
| M\_4\_F\_t\_Set2 | 1.562 | 0 | 0.1090000000000000 | 2204.645000000000 | 56.122000000000000 | 0.000 | 15 |
| M\_4\_F\_t\_Set3 | 1.577 | 0 | 0.1090000000000000 | 2202.264000000000 | 53.741999999999997 | 0.000 | 16 |
| M\_4\_F\_h\_Set1 | 1.649 | 0 | 0.0550000000000000 | 2186.967000000000 | 38.445000000000000 | 0.000 | 17 |
| M\_4\_F\_h\_Set2 | 1.643 | 0 | 0.0550000000000000 | 2183.797000000000 | 35.274999999999999 | 0.000 | 16 |
| M\_4\_F\_h\_Set3 | 1.638 | 0 | 0.0550000000000000 | 2195.983000000000 | 47.460000000000001 | 0.000 | 20 |
| M\_4\_F\_lq\_Set1 | 1.569 | 0 | 0.0550000000000000 | 2197.657000000000 | 49.134999999999998 | 0.000 | 8 |
| M\_4\_F\_lq\_Set2 | 1.579 | 0 | 0.0550000000000000 | 2195.655000000000 | 47.131999999999998 | 0.000 | 9 |
| M\_4\_F\_lq\_Set3 | 1.580 | 0 | 0.0550000000000000 | 2189.535000000000 | 41.012000000000000 | 0.000 | 10 |
| M\_4\_F\_lp\_Set1 | 1.497 | 0 | 0.0550000000000000 | 2215.789000000000 | 67.266999999999996 | 0.000 | 10 |
| M\_4\_F\_lp\_Set2 | 1.479 | 0 | 0.0730000000000000 | 2216.308000000000 | 67.784999999999997 | 0.000 | 11 |
| M\_4\_F\_lp\_Set3 | 1.518 | 0 | 0.1090000000000000 | 2191.296000000000 | 42.774000000000001 | 0.000 | 10 |
| M\_4\_F\_lt\_Set1 | 1.558 | 0 | 0.0910000000000000 | 2185.709000000000 | 37.186000000000000 | 0.000 | 13 |
| M\_4\_F\_lt\_Set2 | 1.566 | 0 | 0.0910000000000000 | 2189.398000000000 | 40.875000000000000 | 0.000 | 15 |
| M\_4\_F\_lt\_Set3 | 1.576 | 0 | 0.0910000000000000 | 2192.901000000000 | 44.378000000000000 | 0.000 | 18 |
| M\_4\_F\_lh\_Set1 | 1.651 | 0 | 0.0550000000000000 | 2166.617000000000 | 18.094000000000001 | 0.000 | 11 |
| M\_4\_F\_lh\_Set2 | 1.655 | 0 | 0.0550000000000000 | 2169.074000000000 | 20.552000000000000 | 0.000 | 13 |
| M\_4\_F\_lh\_Set3 | 1.650 | 0 | 0.0550000000000000 | 2166.435000000000 | 17.913000000000000 | 0.000 | 12 |
| M\_4\_F\_qp\_Set1 | 1.495 | 0 | 0.0910000000000000 | 2209.298000000000 | 60.774999999999999 | 0.000 | 12 |
| M\_4\_F\_qp\_Set2 | 1.477 | 0 | 0.1090000000000000 | 2212.592000000000 | 64.069999999999993 | 0.000 | 15 |
| M\_4\_F\_qp\_Set3 | 1.566 | 0 | 0.0550000000000000 | 2193.130000000000 | 44.607999999999997 | 0.000 | 12 |
| M\_4\_F\_qt\_Set1 | 1.605 | 0 | 0.0730000000000000 | 2180.310000000000 | 31.788000000000000 | 0.000 | 12 |
| M\_4\_F\_qt\_Set2 | 1.604 | 0 | 0.0910000000000000 | 2181.746000000000 | 33.222999999999999 | 0.000 | 15 |
| M\_4\_F\_qt\_Set3 | 1.612 | 0 | 0.0360000000000000 | 2187.790000000000 | 39.268000000000001 | 0.000 | 18 |
| M\_4\_F\_qh\_Set1 | 1.666 | 0 | 0.0550000000000000 | 2171.969000000000 | 23.446999999999999 | 0.000 | 14 |
| M\_4\_F\_qh\_Set2 | 1.668 | 0 | 0.0550000000000000 | 2176.333000000000 | 27.809999999999999 | 0.000 | 16 |
| M\_4\_F\_qh\_Set3 | 1.671 | 0 | 0.0550000000000000 | 2168.625000000000 | 20.102000000000000 | 0.000 | 13 |
| M\_4\_F\_pt\_Set1 | 1.548 | 0 | 0.0910000000000000 | 2186.363000000000 | 37.840000000000003 | 0.000 | 15 |
| M\_4\_F\_pt\_Set2 | 1.547 | 0 | 0.0910000000000000 | 2194.336000000000 | 45.813000000000002 | 0.000 | 18 |
| M\_4\_F\_pt\_Set3 | 1.567 | 0 | 0.0730000000000000 | 2197.692000000000 | 49.170000000000002 | 0.000 | 19 |
| M\_4\_F\_ph\_Set1 | 1.646 | 0 | 0.0550000000000000 | 2167.317000000000 | 18.794000000000000 | 0.000 | 11 |
| M\_4\_F\_ph\_Set2 | 1.645 | 0 | 0.0550000000000000 | 2173.467000000000 | 24.945000000000000 | 0.000 | 14 |
| M\_4\_F\_ph\_Set3 | 1.640 | 0 | 0.0550000000000000 | 2169.442000000000 | 20.920000000000002 | 0.000 | 11 |
| M\_4\_F\_th\_Set1 | 1.655 | 0 | 0.0730000000000000 | 2176.342000000000 | 27.820000000000000 | 0.000 | 15 |
| M\_4\_F\_th\_Set2 | 1.653 | 0 | 0.0730000000000000 | 2176.016000000000 | 27.492999999999999 | 0.000 | 15 |
| M\_4\_F\_th\_Set3 | 1.655 | 0 | 0.0550000000000000 | 2176.100000000000 | 27.577999999999999 | 0.000 | 16 |
| M\_4\_F\_lqp\_Set1 | 1.510 | 0 | 0.0730000000000000 | 2189.470000000000 | 40.948000000000000 | 0.000 | 9 |
| M\_4\_F\_lqp\_Set2 | 1.492 | 0 | 0.0730000000000000 | 2192.120000000000 | 43.597000000000001 | 0.000 | 11 |
| M\_4\_F\_lqp\_Set3 | 1.556 | 0 | 0.0550000000000000 | 2189.593000000000 | 41.070999999999998 | 0.000 | 11 |
| M\_4\_F\_lqt\_Set1 | 1.594 | 0 | 0.0730000000000000 | 2185.643000000000 | 37.121000000000002 | 0.000 | 15 |
| M\_4\_F\_lqt\_Set2 | 1.597 | 0 | 0.0730000000000000 | 2190.706000000000 | 42.183000000000000 | 0.000 | 17 |
| M\_4\_F\_lqt\_Set3 | 1.616 | 0 | 0.0360000000000000 | 2186.054000000000 | 37.530999999999999 | 0.000 | 17 |
| M\_4\_F\_lqh\_Set1 | 1.668 | 0 | 0.0730000000000000 | 2167.917000000000 | 19.393999999999998 | 0.000 | 13 |
| M\_4\_F\_lqh\_Set2 | 1.675 | 0 | 0.0730000000000000 | 2175.912000000000 | 27.388999999999999 | 0.000 | 16 |
| M\_4\_F\_lqh\_Set3 | 1.665 | 0 | 0.0550000000000000 | 2176.591000000000 | 28.068999999999999 | 0.000 | 16 |
| M\_4\_F\_lpt\_Set1 | 1.567 | 0 | 0.0910000000000000 | 2182.219000000000 | 33.695999999999998 | 0.000 | 14 |
| M\_4\_F\_lpt\_Set2 | 1.564 | 0 | 0.0730000000000000 | 2189.332000000000 | 40.808999999999997 | 0.000 | 17 |
| M\_4\_F\_lpt\_Set3 | 1.579 | 0 | 0.0730000000000000 | 2191.546000000000 | 43.024000000000001 | 0.000 | 17 |
| M\_4\_F\_lph\_Set1 | 1.645 | 0 | 0.0550000000000000 | 2178.310000000000 | 29.788000000000000 | 0.000 | 16 |
| M\_4\_F\_lph\_Set2 | 1.648 | 0 | 0.0550000000000000 | 2186.270000000000 | 37.747000000000000 | 0.000 | 19 |
| M\_4\_F\_lph\_Set3 | 1.635 | 0 | 0.0550000000000000 | 2176.485000000000 | 27.963000000000001 | 0.000 | 14 |
| M\_4\_F\_qpt\_Set1 | 1.570 | 0 | 0.1270000000000000 | 2184.876000000000 | 36.353999999999999 | 0.000 | 15 |
| M\_4\_F\_qpt\_Set2 | 1.568 | 0 | 0.1090000000000000 | 2187.206000000000 | 38.683000000000000 | 0.000 | 16 |
| M\_4\_F\_qpt\_Set3 | 1.593 | 0 | 0.0730000000000000 | 2185.329000000000 | 36.807000000000002 | 0.000 | 16 |
| M\_4\_F\_qph\_Set1 | 1.642 | 0 | 0.0550000000000000 | 2184.894000000000 | 36.371000000000002 | 0.000 | 18 |
| M\_4\_F\_qph\_Set2 | 1.645 | 0 | 0.0550000000000000 | 2193.099000000000 | 44.576000000000001 | 0.000 | 21 |
| M\_4\_F\_qph\_Set3 | 1.643 | 0 | 0.0550000000000000 | 2188.125000000000 | 39.601999999999997 | 0.000 | 18 |
| M\_4\_F\_qth\_Set1 | 1.669 | 0 | 0.0730000000000000 | 2166.746000000000 | 18.224000000000000 | 0.000 | 14 |
| M\_4\_F\_qth\_Set2 | 1.668 | 0 | 0.0730000000000000 | 2173.738000000000 | 25.216000000000001 | 0.000 | 17 |
| M\_4\_F\_qth\_Set3 | 1.674 | 0 | 0.0360000000000000 | 2173.567000000000 | 25.045000000000002 | 0.000 | 18 |
| M\_4\_F\_pth\_Set1 | 1.656 | 0 | 0.0730000000000000 | 2166.520000000000 | 17.998000000000001 | 0.000 | 13 |
| M\_4\_F\_pth\_Set2 | 1.661 | 0 | 0.0730000000000000 | 2176.099000000000 | 27.577000000000002 | 0.000 | 17 |
| M\_4\_F\_pth\_Set3 | 1.649 | 0 | 0.0550000000000000 | 2168.792000000000 | 20.270000000000000 | 0.000 | 15 |
| M\_4\_F\_lqpt\_Set1 | 1.570 | 0 | 0.1090000000000000 | 2185.397000000000 | 36.875000000000000 | 0.000 | 14 |
| M\_4\_F\_lqpt\_Set2 | 1.570 | 0 | 0.0910000000000000 | 2190.652000000000 | 42.130000000000003 | 0.000 | 16 |
| M\_4\_F\_lqpt\_Set3 | 1.591 | 0 | 0.0730000000000000 | 2188.218000000000 | 39.695999999999998 | 0.000 | 17 |
| M\_4\_F\_lqph\_Set1 | 1.638 | 0 | 0.0550000000000000 | 2176.857000000000 | 28.334000000000000 | 0.000 | 16 |
| M\_4\_F\_lqph\_Set2 | 1.646 | 0 | 0.0550000000000000 | 2182.485000000000 | 33.962000000000003 | 0.000 | 18 |
| M\_4\_F\_lqph\_Set3 | 1.637 | 0 | 0.0550000000000000 | 2178.759000000000 | 30.236000000000001 | 0.000 | 15 |
| M\_4\_F\_lqth\_Set1 | 1.675 | 0 | 0.0730000000000000 | 2162.109000000000 | 13.587000000000000 | 0.000 | 13 |
| M\_4\_F\_lqth\_Set2 | 1.674 | 0 | 0.0730000000000000 | 2164.491000000000 | 15.968000000000000 | 0.000 | 14 |
| M\_4\_F\_lqth\_Set3 | 1.677 | 0 | 0.0550000000000000 | 2170.033000000000 | 21.510999999999999 | 0.000 | 17 |
| M\_4\_F\_lpth\_Set1 | 1.653 | 0 | 0.0730000000000000 | 2172.364000000000 | 23.841999999999999 | 0.000 | 16 |
| M\_4\_F\_lpth\_Set2 | 1.651 | 0 | 0.0730000000000000 | 2175.087000000000 | 26.564000000000000 | 0.000 | 17 |
| M\_4\_F\_lpth\_Set3 | 1.657 | 0 | 0.0550000000000000 | 2170.642000000000 | 22.120000000000001 | 0.000 | 16 |
| M\_4\_F\_lqpth\_Set1 | 1.655 | 0 | 0.0730000000000000 | 2173.882000000000 | 25.359000000000002 | 0.000 | 17 |
| M\_4\_F\_lqpth\_Set2 | 1.658 | 0 | 0.0730000000000000 | 2179.592000000000 | 31.068999999999999 | 0.000 | 19 |
| M\_4\_F\_lqpth\_Set3 | 1.656 | 0 | 0.0550000000000000 | 2173.586000000000 | 25.064000000000000 | 0.000 | 17 |
| M\_5\_F\_l\_Set1 | 1.481 | 0 | 0.0910000000000000 | 2274.513000000000 | 125.991000000000000 | 0.000 | 5 |
| M\_5\_F\_l\_Set2 | 1.489 | 0 | 0.0910000000000000 | 2274.483000000000 | 125.959999999999994 | 0.000 | 5 |
| M\_5\_F\_l\_Set3 | 1.478 | 0 | 0.0910000000000000 | 2275.432000000000 | 126.909000000000006 | 0.000 | 5 |
| M\_5\_F\_q\_Set1 | 1.506 | 0 | 0.0730000000000000 | 2244.675000000000 | 96.153000000000006 | 0.000 | 6 |
| M\_5\_F\_q\_Set2 | 1.509 | 0 | 0.0730000000000000 | 2216.588000000000 | 68.064999999999998 | 0.000 | 7 |
| M\_5\_F\_q\_Set3 | 1.528 | 0 | 0.0550000000000000 | 2204.845000000000 | 56.323000000000000 | 0.000 | 8 |
| M\_5\_F\_p\_Set1 | 1.487 | 0 | 0.0910000000000000 | 2252.273000000000 | 103.751000000000005 | 0.000 | 8 |
| M\_5\_F\_p\_Set2 | 1.460 | 0 | 0.0910000000000000 | 2243.302000000000 | 94.780000000000001 | 0.000 | 9 |
| M\_5\_F\_p\_Set3 | 1.474 | 0 | 0.0730000000000000 | 2204.118000000000 | 55.595999999999997 | 0.000 | 11 |
| M\_5\_F\_t\_Set1 | 1.556 | 0 | 0.1090000000000000 | 2216.142000000000 | 67.620000000000005 | 0.000 | 9 |
| M\_5\_F\_t\_Set2 | 1.559 | 0 | 0.1090000000000000 | 2216.135000000000 | 67.611999999999995 | 0.000 | 9 |
| M\_5\_F\_t\_Set3 | 1.578 | 0 | 0.1090000000000000 | 2209.405000000000 | 60.881999999999998 | 0.000 | 10 |
| M\_5\_F\_h\_Set1 | 1.599 | 0 | 0.0550000000000000 | 2197.407000000000 | 48.884999999999998 | 0.000 | 13 |
| M\_5\_F\_h\_Set2 | 1.599 | 0 | 0.0550000000000000 | 2199.969000000000 | 51.445999999999998 | 0.000 | 14 |
| M\_5\_F\_h\_Set3 | 1.591 | 0 | 0.0550000000000000 | 2212.355000000000 | 63.832000000000001 | 0.000 | 18 |
| M\_5\_F\_lq\_Set1 | 1.508 | 0 | 0.0910000000000000 | 2201.622000000000 | 53.100000000000001 | 0.000 | 8 |
| M\_5\_F\_lq\_Set2 | 1.516 | 0 | 0.0910000000000000 | 2201.334000000000 | 52.811000000000000 | 0.000 | 9 |
| M\_5\_F\_lq\_Set3 | 1.534 | 0 | 0.0360000000000000 | 2193.319000000000 | 44.795999999999999 | 0.000 | 9 |
| M\_5\_F\_lp\_Set1 | 1.488 | 0 | 0.0550000000000000 | 2225.639000000000 | 77.117000000000004 | 0.000 | 9 |
| M\_5\_F\_lp\_Set2 | 1.475 | 0 | 0.0730000000000000 | 2224.713000000000 | 76.191000000000003 | 0.000 | 10 |
| M\_5\_F\_lp\_Set3 | 1.489 | 0 | 0.1090000000000000 | 2194.563000000000 | 46.040999999999997 | 0.000 | 10 |
| M\_5\_F\_lt\_Set1 | 1.551 | 0 | 0.0910000000000000 | 2197.222000000000 | 48.698999999999998 | 0.000 | 10 |
| M\_5\_F\_lt\_Set2 | 1.557 | 0 | 0.0910000000000000 | 2201.607000000000 | 53.085000000000001 | 0.000 | 12 |
| M\_5\_F\_lt\_Set3 | 1.559 | 0 | 0.0910000000000000 | 2194.988000000000 | 46.466000000000001 | 0.000 | 12 |
| M\_5\_F\_lh\_Set1 | 1.620 | 0 | 0.0550000000000000 | 2176.668000000000 | 28.145000000000000 | 0.000 | 11 |
| M\_5\_F\_lh\_Set2 | 1.618 | 0 | 0.0550000000000000 | 2179.374000000000 | 30.850999999999999 | 0.000 | 13 |
| M\_5\_F\_lh\_Set3 | 1.616 | 0 | 0.0550000000000000 | 2179.338000000000 | 30.815999999999999 | 0.000 | 13 |
| M\_5\_F\_qp\_Set1 | 1.501 | 0 | 0.0910000000000000 | 2214.700000000000 | 66.177999999999997 | 0.000 | 11 |
| M\_5\_F\_qp\_Set2 | 1.474 | 0 | 0.1090000000000000 | 2219.438000000000 | 70.915999999999997 | 0.000 | 14 |
| M\_5\_F\_qp\_Set3 | 1.522 | 0 | 0.0550000000000000 | 2198.003000000000 | 49.479999999999997 | 0.000 | 12 |
| M\_5\_F\_qt\_Set1 | 1.594 | 0 | 0.0550000000000000 | 2190.983000000000 | 42.460999999999999 | 0.000 | 9 |
| M\_5\_F\_qt\_Set2 | 1.591 | 0 | 0.0550000000000000 | 2193.006000000000 | 44.484000000000002 | 0.000 | 12 |
| M\_5\_F\_qt\_Set3 | 1.591 | 0 | 0.0360000000000000 | 2196.648000000000 | 48.125999999999998 | 0.000 | 15 |
| M\_5\_F\_qh\_Set1 | 1.612 | 0 | 0.0550000000000000 | 2180.819000000000 | 32.296999999999997 | 0.000 | 13 |
| M\_5\_F\_qh\_Set2 | 1.612 | 0 | 0.0550000000000000 | 2181.049000000000 | 32.526000000000003 | 0.000 | 14 |
| M\_5\_F\_qh\_Set3 | 1.619 | 0 | 0.0550000000000000 | 2181.015000000000 | 32.491999999999997 | 0.000 | 14 |
| M\_5\_F\_pt\_Set1 | 1.531 | 0 | 0.0910000000000000 | 2198.204000000000 | 49.682000000000002 | 0.000 | 12 |
| M\_5\_F\_pt\_Set2 | 1.531 | 0 | 0.1090000000000000 | 2203.030000000000 | 54.506999999999998 | 0.000 | 15 |
| M\_5\_F\_pt\_Set3 | 1.549 | 0 | 0.0550000000000000 | 2195.243000000000 | 46.719999999999999 | 0.000 | 15 |
| M\_5\_F\_ph\_Set1 | 1.602 | 0 | 0.0550000000000000 | 2175.490000000000 | 26.968000000000000 | 0.000 | 11 |
| M\_5\_F\_ph\_Set2 | 1.600 | 0 | 0.0550000000000000 | 2174.655000000000 | 26.132000000000001 | 0.000 | 11 |
| M\_5\_F\_ph\_Set3 | 1.589 | 0 | 0.0550000000000000 | 2179.123000000000 | 30.600999999999999 | 0.000 | 11 |
| M\_5\_F\_th\_Set1 | 1.610 | 0 | 0.0550000000000000 | 2197.278000000000 | 48.756000000000000 | 0.000 | 15 |
| M\_5\_F\_th\_Set2 | 1.605 | 0 | 0.0550000000000000 | 2199.837000000000 | 51.314000000000000 | 0.000 | 16 |
| M\_5\_F\_th\_Set3 | 1.600 | 0 | 0.0550000000000000 | 2191.720000000000 | 43.198000000000000 | 0.000 | 14 |
| M\_5\_F\_lqp\_Set1 | 1.504 | 0 | 0.0550000000000000 | 2193.754000000000 | 45.231000000000002 | 0.000 | 9 |
| M\_5\_F\_lqp\_Set2 | 1.487 | 0 | 0.0550000000000000 | 2196.127000000000 | 47.604999999999997 | 0.000 | 10 |
| M\_5\_F\_lqp\_Set3 | 1.534 | 0 | 0.0550000000000000 | 2191.486000000000 | 42.963999999999999 | 0.000 | 10 |
| M\_5\_F\_lqt\_Set1 | 1.568 | 0 | 0.0910000000000000 | 2189.175000000000 | 40.652000000000001 | 0.000 | 11 |
| M\_5\_F\_lqt\_Set2 | 1.574 | 0 | 0.0910000000000000 | 2189.210000000000 | 40.688000000000002 | 0.000 | 11 |
| M\_5\_F\_lqt\_Set3 | 1.575 | 0 | 0.0360000000000000 | 2187.867000000000 | 39.344999999999999 | 0.000 | 13 |
| M\_5\_F\_lqh\_Set1 | 1.627 | 0 | 0.0550000000000000 | 2179.320000000000 | 30.797000000000001 | 0.000 | 14 |
| M\_5\_F\_lqh\_Set2 | 1.621 | 0 | 0.0550000000000000 | 2174.146000000000 | 25.623999999999999 | 0.000 | 12 |
| M\_5\_F\_lqh\_Set3 | 1.616 | 0 | 0.0550000000000000 | 2177.983000000000 | 29.460999999999999 | 0.000 | 13 |
| M\_5\_F\_lpt\_Set1 | 1.564 | 0 | 0.0910000000000000 | 2193.560000000000 | 45.036999999999999 | 0.000 | 12 |
| M\_5\_F\_lpt\_Set2 | 1.564 | 0 | 0.0910000000000000 | 2197.658000000000 | 49.134999999999998 | 0.000 | 14 |
| M\_5\_F\_lpt\_Set3 | 1.573 | 0 | 0.0730000000000000 | 2192.561000000000 | 44.039000000000001 | 0.000 | 15 |
| M\_5\_F\_lph\_Set1 | 1.600 | 0 | 0.0550000000000000 | 2182.683000000000 | 34.159999999999997 | 0.000 | 14 |
| M\_5\_F\_lph\_Set2 | 1.601 | 0 | 0.0550000000000000 | 2177.167000000000 | 28.645000000000000 | 0.000 | 12 |
| M\_5\_F\_lph\_Set3 | 1.594 | 0 | 0.0550000000000000 | 2186.880000000000 | 38.356999999999999 | 0.000 | 14 |
| M\_5\_F\_qpt\_Set1 | 1.566 | 0 | 0.0910000000000000 | 2191.708000000000 | 43.185000000000002 | 0.000 | 12 |
| M\_5\_F\_qpt\_Set2 | 1.558 | 0 | 0.0730000000000000 | 2195.664000000000 | 47.140999999999998 | 0.000 | 14 |
| M\_5\_F\_qpt\_Set3 | 1.590 | 0 | 0.0550000000000000 | 2187.836000000000 | 39.313000000000002 | 0.000 | 14 |
| M\_5\_F\_qph\_Set1 | 1.598 | 0 | 0.0550000000000000 | 2188.946000000000 | 40.423000000000002 | 0.000 | 16 |
| M\_5\_F\_qph\_Set2 | 1.599 | 0 | 0.0550000000000000 | 2191.704000000000 | 43.180999999999997 | 0.000 | 17 |
| M\_5\_F\_qph\_Set3 | 1.593 | 0 | 0.0550000000000000 | 2196.184000000000 | 47.661999999999999 | 0.000 | 17 |
| M\_5\_F\_qth\_Set1 | 1.621 | 0 | 0.0550000000000000 | 2180.286000000000 | 31.763000000000002 | 0.000 | 15 |
| M\_5\_F\_qth\_Set2 | 1.618 | 0 | 0.0550000000000000 | 2177.788000000000 | 29.265999999999998 | 0.000 | 15 |
| M\_5\_F\_qth\_Set3 | 1.623 | 0 | 0.0550000000000000 | 2171.567000000000 | 23.045000000000002 | 0.000 | 14 |
| M\_5\_F\_pth\_Set1 | 1.604 | 0 | 0.0550000000000000 | 2177.131000000000 | 28.609000000000002 | 0.000 | 14 |
| M\_5\_F\_pth\_Set2 | 1.608 | 0 | 0.0550000000000000 | 2175.582000000000 | 27.059000000000001 | 0.000 | 14 |
| M\_5\_F\_pth\_Set3 | 1.601 | 0 | 0.0550000000000000 | 2172.973000000000 | 24.449999999999999 | 0.000 | 13 |
| M\_5\_F\_lqpt\_Set1 | 1.579 | 0 | 0.0910000000000000 | 2188.963000000000 | 40.439999999999998 | 0.000 | 12 |
| M\_5\_F\_lqpt\_Set2 | 1.561 | 0 | 0.0910000000000000 | 2188.990000000000 | 40.468000000000004 | 0.000 | 12 |
| M\_5\_F\_lqpt\_Set3 | 1.582 | 0 | 0.0550000000000000 | 2187.252000000000 | 38.728999999999999 | 0.000 | 14 |
| M\_5\_F\_lqph\_Set1 | 1.598 | 0 | 0.0550000000000000 | 2180.998000000000 | 32.475999999999999 | 0.000 | 14 |
| M\_5\_F\_lqph\_Set2 | 1.594 | 0 | 0.0550000000000000 | 2184.033000000000 | 35.509999999999998 | 0.000 | 15 |
| M\_5\_F\_lqph\_Set3 | 1.593 | 0 | 0.0550000000000000 | 2179.257000000000 | 30.734000000000002 | 0.000 | 11 |
| M\_5\_F\_lqth\_Set1 | 1.626 | 0 | 0.0730000000000000 | 2170.450000000000 | 21.928000000000001 | 0.000 | 13 |
| M\_5\_F\_lqth\_Set2 | 1.629 | 0 | 0.0730000000000000 | 2173.009000000000 | 24.486000000000001 | 0.000 | 14 |
| M\_5\_F\_lqth\_Set3 | 1.622 | 0 | 0.0550000000000000 | 2173.573000000000 | 25.050000000000001 | 0.000 | 15 |
| M\_5\_F\_lpth\_Set1 | 1.609 | 0 | 0.0550000000000000 | 2178.046000000000 | 29.523000000000000 | 0.000 | 15 |
| M\_5\_F\_lpth\_Set2 | 1.612 | 0 | 0.0550000000000000 | 2175.326000000000 | 26.803999999999998 | 0.000 | 14 |
| M\_5\_F\_lpth\_Set3 | 1.607 | 0 | 0.0550000000000000 | 2174.157000000000 | 25.634000000000000 | 0.000 | 14 |
| M\_5\_F\_lqpth\_Set1 | 1.609 | 0 | 0.0550000000000000 | 2179.880000000000 | 31.356999999999999 | 0.000 | 16 |
| M\_5\_F\_lqpth\_Set2 | 1.612 | 0 | 0.0550000000000000 | 2179.824000000000 | 31.302000000000000 | 0.000 | 16 |
| M\_5\_F\_lqpth\_Set3 | 1.601 | 0 | 0.0550000000000000 | 2178.040000000000 | 29.516999999999999 | 0.000 | 15 |
| M\_6\_F\_l\_Set1 | 1.485 | 0 | 0.0730000000000000 | 2275.742000000000 | 127.218999999999994 | 0.000 | 5 |
| M\_6\_F\_l\_Set2 | 1.481 | 0 | 0.0730000000000000 | 2275.711000000000 | 127.188999999999993 | 0.000 | 5 |
| M\_6\_F\_l\_Set3 | 1.472 | 0 | 0.0730000000000000 | 2275.950000000000 | 127.427000000000007 | 0.000 | 5 |
| M\_6\_F\_q\_Set1 | 1.502 | 0 | 0.0550000000000000 | 2245.527000000000 | 97.004999999999995 | 0.000 | 5 |
| M\_6\_F\_q\_Set2 | 1.496 | 0 | 0.0550000000000000 | 2222.699000000000 | 74.177000000000007 | 0.000 | 7 |
| M\_6\_F\_q\_Set3 | 1.515 | 0 | 0.0550000000000000 | 2212.841000000000 | 64.319000000000003 | 0.000 | 8 |
| M\_6\_F\_p\_Set1 | 1.491 | 0 | 0.0910000000000000 | 2252.609000000000 | 104.085999999999999 | 0.000 | 7 |
| M\_6\_F\_p\_Set2 | 1.468 | 0 | 0.0910000000000000 | 2243.633000000000 | 95.109999999999999 | 0.000 | 8 |
| M\_6\_F\_p\_Set3 | 1.468 | 0 | 0.0730000000000000 | 2206.535000000000 | 58.012000000000000 | 0.000 | 10 |
| M\_6\_F\_t\_Set1 | 1.459 | 0 | 0.1090000000000000 | 2223.604000000000 | 75.081000000000003 | 0.000 | 7 |
| M\_6\_F\_t\_Set2 | 1.462 | 0 | 0.1090000000000000 | 2223.593000000000 | 75.070999999999998 | 0.000 | 7 |
| M\_6\_F\_t\_Set3 | 1.487 | 0 | 0.1090000000000000 | 2214.115000000000 | 65.591999999999999 | 0.000 | 7 |
| M\_6\_F\_h\_Set1 | 1.580 | 0 | 0.0550000000000000 | 2210.418000000000 | 61.896000000000001 | 0.000 | 13 |
| M\_6\_F\_h\_Set2 | 1.573 | 0 | 0.0550000000000000 | 2208.275000000000 | 59.752000000000002 | 0.000 | 12 |
| M\_6\_F\_h\_Set3 | 1.580 | 0 | 0.0550000000000000 | 2220.743000000000 | 72.219999999999999 | 0.000 | 16 |
| M\_6\_F\_lq\_Set1 | 1.514 | 0 | 0.0730000000000000 | 2206.397000000000 | 57.874000000000002 | 0.000 | 8 |
| M\_6\_F\_lq\_Set2 | 1.510 | 0 | 0.0730000000000000 | 2206.284000000000 | 57.762000000000000 | 0.000 | 8 |
| M\_6\_F\_lq\_Set3 | 1.526 | 0 | 0.0360000000000000 | 2199.164000000000 | 50.640999999999998 | 0.000 | 9 |
| M\_6\_F\_lp\_Set1 | 1.480 | 0 | 0.0550000000000000 | 2238.803000000000 | 90.280000000000001 | 0.000 | 9 |
| M\_6\_F\_lp\_Set2 | 1.465 | 0 | 0.0730000000000000 | 2235.861000000000 | 87.338999999999999 | 0.000 | 10 |
| M\_6\_F\_lp\_Set3 | 1.481 | 0 | 0.0730000000000000 | 2201.415000000000 | 52.892000000000003 | 0.000 | 11 |
| M\_6\_F\_lt\_Set1 | 1.528 | 0 | 0.0730000000000000 | 2208.327000000000 | 59.805000000000000 | 0.000 | 9 |
| M\_6\_F\_lt\_Set2 | 1.523 | 0 | 0.0730000000000000 | 2210.610000000000 | 62.088000000000001 | 0.000 | 10 |
| M\_6\_F\_lt\_Set3 | 1.521 | 0 | 0.0910000000000000 | 2205.482000000000 | 56.959000000000003 | 0.000 | 11 |
| M\_6\_F\_lh\_Set1 | 1.570 | 0 | 0.0550000000000000 | 2188.189000000000 | 39.667000000000002 | 0.000 | 11 |
| M\_6\_F\_lh\_Set2 | 1.577 | 0 | 0.0550000000000000 | 2188.486000000000 | 39.963999999999999 | 0.000 | 12 |
| M\_6\_F\_lh\_Set3 | 1.558 | 0 | 0.0550000000000000 | 2190.997000000000 | 42.475000000000001 | 0.000 | 13 |
| M\_6\_F\_qp\_Set1 | 1.483 | 0 | 0.0910000000000000 | 2222.153000000000 | 73.631000000000000 | 0.000 | 11 |
| M\_6\_F\_qp\_Set2 | 1.460 | 0 | 0.0910000000000000 | 2219.968000000000 | 71.444999999999993 | 0.000 | 11 |
| M\_6\_F\_qp\_Set3 | 1.507 | 0 | 0.0550000000000000 | 2199.873000000000 | 51.350000000000001 | 0.000 | 11 |
| M\_6\_F\_qt\_Set1 | 1.564 | 0 | 0.0730000000000000 | 2199.160000000000 | 50.637999999999998 | 0.000 | 8 |
| M\_6\_F\_qt\_Set2 | 1.566 | 0 | 0.0730000000000000 | 2201.374000000000 | 52.851999999999997 | 0.000 | 11 |
| M\_6\_F\_qt\_Set3 | 1.562 | 0 | 0.0550000000000000 | 2200.158000000000 | 51.636000000000003 | 0.000 | 12 |
| M\_6\_F\_qh\_Set1 | 1.582 | 0 | 0.0550000000000000 | 2194.785000000000 | 46.262999999999998 | 0.000 | 13 |
| M\_6\_F\_qh\_Set2 | 1.591 | 0 | 0.0550000000000000 | 2191.460000000000 | 42.938000000000002 | 0.000 | 14 |
| M\_6\_F\_qh\_Set3 | 1.588 | 0 | 0.0550000000000000 | 2191.440000000000 | 42.917999999999999 | 0.000 | 14 |
| M\_6\_F\_pt\_Set1 | 1.533 | 0 | 0.0910000000000000 | 2209.779000000000 | 61.256999999999998 | 0.000 | 12 |
| M\_6\_F\_pt\_Set2 | 1.531 | 0 | 0.1090000000000000 | 2218.032000000000 | 69.510000000000005 | 0.000 | 16 |
| M\_6\_F\_pt\_Set3 | 1.543 | 0 | 0.0550000000000000 | 2200.371000000000 | 51.848999999999997 | 0.000 | 14 |
| M\_6\_F\_ph\_Set1 | 1.580 | 0 | 0.0550000000000000 | 2184.396000000000 | 35.872999999999998 | 0.000 | 11 |
| M\_6\_F\_ph\_Set2 | 1.581 | 0 | 0.0550000000000000 | 2188.658000000000 | 40.136000000000003 | 0.000 | 13 |
| M\_6\_F\_ph\_Set3 | 1.578 | 0 | 0.0550000000000000 | 2182.894000000000 | 34.371000000000002 | 0.000 | 10 |
| M\_6\_F\_th\_Set1 | 1.571 | 0 | 0.0550000000000000 | 2196.670000000000 | 48.148000000000003 | 0.000 | 10 |
| M\_6\_F\_th\_Set2 | 1.578 | 0 | 0.0550000000000000 | 2198.995000000000 | 50.472999999999999 | 0.000 | 11 |
| M\_6\_F\_th\_Set3 | 1.578 | 0 | 0.0550000000000000 | 2197.345000000000 | 48.823000000000000 | 0.000 | 12 |
| M\_6\_F\_lqp\_Set1 | 1.503 | 0 | 0.0550000000000000 | 2198.479000000000 | 49.956000000000003 | 0.000 | 9 |
| M\_6\_F\_lqp\_Set2 | 1.491 | 0 | 0.0550000000000000 | 2198.541000000000 | 50.018999999999998 | 0.000 | 9 |
| M\_6\_F\_lqp\_Set3 | 1.516 | 0 | 0.0550000000000000 | 2194.162000000000 | 45.640000000000001 | 0.000 | 10 |
| M\_6\_F\_lqt\_Set1 | 1.551 | 0 | 0.0730000000000000 | 2195.799000000000 | 47.277000000000001 | 0.000 | 10 |
| M\_6\_F\_lqt\_Set2 | 1.554 | 0 | 0.0730000000000000 | 2195.567000000000 | 47.045000000000002 | 0.000 | 10 |
| M\_6\_F\_lqt\_Set3 | 1.547 | 0 | 0.0360000000000000 | 2194.079000000000 | 45.555999999999997 | 0.000 | 12 |
| M\_6\_F\_lqh\_Set1 | 1.569 | 0 | 0.0550000000000000 | 2180.945000000000 | 32.421999999999997 | 0.000 | 11 |
| M\_6\_F\_lqh\_Set2 | 1.570 | 0 | 0.0550000000000000 | 2181.036000000000 | 32.514000000000003 | 0.000 | 11 |
| M\_6\_F\_lqh\_Set3 | 1.558 | 0 | 0.0550000000000000 | 2185.502000000000 | 36.978999999999999 | 0.000 | 12 |
| M\_6\_F\_lpt\_Set1 | 1.551 | 0 | 0.0910000000000000 | 2204.732000000000 | 56.210000000000001 | 0.000 | 12 |
| M\_6\_F\_lpt\_Set2 | 1.552 | 0 | 0.1090000000000000 | 2203.477000000000 | 54.954000000000001 | 0.000 | 12 |
| M\_6\_F\_lpt\_Set3 | 1.564 | 0 | 0.0730000000000000 | 2201.640000000000 | 53.116999999999997 | 0.000 | 15 |
| M\_6\_F\_lph\_Set1 | 1.577 | 0 | 0.0550000000000000 | 2189.610000000000 | 41.088000000000001 | 0.000 | 13 |
| M\_6\_F\_lph\_Set2 | 1.577 | 0 | 0.0550000000000000 | 2186.345000000000 | 37.823000000000000 | 0.000 | 12 |
| M\_6\_F\_lph\_Set3 | 1.579 | 0 | 0.0550000000000000 | 2177.673000000000 | 29.151000000000000 | 0.000 | 8 |
| M\_6\_F\_qpt\_Set1 | 1.563 | 0 | 0.0730000000000000 | 2206.981000000000 | 58.457999999999998 | 0.000 | 14 |
| M\_6\_F\_qpt\_Set2 | 1.561 | 0 | 0.0910000000000000 | 2206.952000000000 | 58.429000000000002 | 0.000 | 14 |
| M\_6\_F\_qpt\_Set3 | 1.581 | 0 | 0.0550000000000000 | 2193.857000000000 | 45.335000000000001 | 0.000 | 14 |
| M\_6\_F\_qph\_Set1 | 1.573 | 0 | 0.0550000000000000 | 2193.484000000000 | 44.962000000000003 | 0.000 | 14 |
| M\_6\_F\_qph\_Set2 | 1.571 | 0 | 0.0550000000000000 | 2190.585000000000 | 42.061999999999998 | 0.000 | 13 |
| M\_6\_F\_qph\_Set3 | 1.580 | 0 | 0.0550000000000000 | 2189.975000000000 | 41.453000000000003 | 0.000 | 12 |
| M\_6\_F\_qth\_Set1 | 1.591 | 0 | 0.0550000000000000 | 2189.691000000000 | 41.167999999999999 | 0.000 | 14 |
| M\_6\_F\_qth\_Set2 | 1.593 | 0 | 0.0550000000000000 | 2185.032000000000 | 36.509000000000000 | 0.000 | 14 |
| M\_6\_F\_qth\_Set3 | 1.598 | 0 | 0.0550000000000000 | 2184.414000000000 | 35.892000000000003 | 0.000 | 15 |
| M\_6\_F\_pth\_Set1 | 1.576 | 0 | 0.0550000000000000 | 2177.612000000000 | 29.090000000000000 | 0.000 | 11 |
| M\_6\_F\_pth\_Set2 | 1.571 | 0 | 0.0550000000000000 | 2178.937000000000 | 30.414999999999999 | 0.000 | 12 |
| M\_6\_F\_pth\_Set3 | 1.580 | 0 | 0.0550000000000000 | 2173.289000000000 | 24.766999999999999 | 0.000 | 10 |
| M\_6\_F\_lqpt\_Set1 | 1.566 | 0 | 0.0910000000000000 | 2197.098000000000 | 48.576000000000001 | 0.000 | 12 |
| M\_6\_F\_lqpt\_Set2 | 1.564 | 0 | 0.0910000000000000 | 2196.728000000000 | 48.206000000000003 | 0.000 | 12 |
| M\_6\_F\_lqpt\_Set3 | 1.582 | 0 | 0.0550000000000000 | 2190.912000000000 | 42.390000000000001 | 0.000 | 13 |
| M\_6\_F\_lqph\_Set1 | 1.572 | 0 | 0.0550000000000000 | 2200.527000000000 | 52.003999999999998 | 0.000 | 17 |
| M\_6\_F\_lqph\_Set2 | 1.579 | 0 | 0.0550000000000000 | 2197.170000000000 | 48.648000000000003 | 0.000 | 16 |
| M\_6\_F\_lqph\_Set3 | 1.573 | 0 | 0.0550000000000000 | 2183.669000000000 | 35.146999999999998 | 0.000 | 10 |
| M\_6\_F\_lqth\_Set1 | 1.570 | 0 | 0.0550000000000000 | 2174.714000000000 | 26.190999999999999 | 0.000 | 11 |
| M\_6\_F\_lqth\_Set2 | 1.572 | 0 | 0.0550000000000000 | 2177.309000000000 | 28.786999999999999 | 0.000 | 12 |
| M\_6\_F\_lqth\_Set3 | 1.564 | 0 | 0.0550000000000000 | 2183.045000000000 | 34.523000000000003 | 0.000 | 15 |
| M\_6\_F\_lpth\_Set1 | 1.585 | 0 | 0.0550000000000000 | 2181.753000000000 | 33.231000000000002 | 0.000 | 13 |
| M\_6\_F\_lpth\_Set2 | 1.579 | 0 | 0.0550000000000000 | 2178.940000000000 | 30.417999999999999 | 0.000 | 12 |
| M\_6\_F\_lpth\_Set3 | 1.580 | 0 | 0.0550000000000000 | 2175.779000000000 | 27.256000000000000 | 0.000 | 11 |
| M\_6\_F\_lqpth\_Set1 | 1.579 | 0 | 0.0550000000000000 | 2178.992000000000 | 30.469999999999999 | 0.000 | 12 |
| M\_6\_F\_lqpth\_Set2 | 1.581 | 0 | 0.0550000000000000 | 2181.915000000000 | 33.393000000000001 | 0.000 | 13 |
| M\_6\_F\_lqpth\_Set3 | 1.583 | 0 | 0.0550000000000000 | 2185.259000000000 | 36.737000000000002 | 0.000 | 14 |
| M\_8\_F\_l\_Set1 | 1.489 | 0 | 0.0550000000000000 | 2278.757000000000 | 130.234000000000009 | 0.000 | 5 |
| M\_8\_F\_l\_Set2 | 1.484 | 0 | 0.0550000000000000 | 2278.727000000000 | 130.204000000000008 | 0.000 | 5 |
| M\_8\_F\_l\_Set3 | 1.474 | 0 | 0.0730000000000000 | 2277.320000000000 | 128.798000000000002 | 0.000 | 5 |
| M\_8\_F\_q\_Set1 | 1.500 | 0 | 0.0550000000000000 | 2252.239000000000 | 103.716999999999999 | 0.000 | 5 |
| M\_8\_F\_q\_Set2 | 1.495 | 0 | 0.0550000000000000 | 2238.280000000000 | 89.757999999999996 | 0.000 | 7 |
| M\_8\_F\_q\_Set3 | 1.517 | 0 | 0.0550000000000000 | 2229.654000000000 | 81.132000000000005 | 0.000 | 7 |
| M\_8\_F\_p\_Set1 | 1.487 | 0 | 0.0550000000000000 | 2253.811000000000 | 105.289000000000001 | 0.000 | 6 |
| M\_8\_F\_p\_Set2 | 1.478 | 0 | 0.0910000000000000 | 2247.294000000000 | 98.772000000000006 | 0.000 | 8 |
| M\_8\_F\_p\_Set3 | 1.471 | 0 | 0.0910000000000000 | 2212.593000000000 | 64.070999999999998 | 0.000 | 10 |
| M\_8\_F\_t\_Set1 | 1.290 | 0 | 0.0360000000000000 | 2245.934000000000 | 97.411000000000001 | 0.000 | 6 |
| M\_8\_F\_t\_Set2 | 1.292 | 0 | 0.0360000000000000 | 2245.916000000000 | 97.393000000000001 | 0.000 | 6 |
| M\_8\_F\_t\_Set3 | 1.340 | 0 | 0.0360000000000000 | 2235.005000000000 | 86.483000000000004 | 0.000 | 6 |
| M\_8\_F\_h\_Set1 | 1.556 | 0 | 0.0550000000000000 | 2232.609000000000 | 84.087000000000003 | 0.000 | 13 |
| M\_8\_F\_h\_Set2 | 1.553 | 0 | 0.0550000000000000 | 2232.519000000000 | 83.997000000000000 | 0.000 | 13 |
| M\_8\_F\_h\_Set3 | 1.553 | 0 | 0.0730000000000000 | 2232.407000000000 | 83.885000000000005 | 0.000 | 12 |
| M\_8\_F\_lq\_Set1 | 1.521 | 0 | 0.0550000000000000 | 2213.871000000000 | 65.349000000000004 | 0.000 | 7 |
| M\_8\_F\_lq\_Set2 | 1.516 | 0 | 0.0550000000000000 | 2213.896000000000 | 65.373999999999995 | 0.000 | 7 |
| M\_8\_F\_lq\_Set3 | 1.520 | 0 | 0.0360000000000000 | 2211.933000000000 | 63.409999999999997 | 0.000 | 8 |
| M\_8\_F\_lp\_Set1 | 1.452 | 0 | 0.0550000000000000 | 2250.260000000000 | 101.736999999999995 | 0.000 | 7 |
| M\_8\_F\_lp\_Set2 | 1.471 | 0 | 0.0550000000000000 | 2240.469000000000 | 91.945999999999998 | 0.000 | 7 |
| M\_8\_F\_lp\_Set3 | 1.481 | 0 | 0.0730000000000000 | 2211.368000000000 | 62.845999999999997 | 0.000 | 10 |
| M\_8\_F\_lt\_Set1 | 1.491 | 0 | 0.0550000000000000 | 2225.770000000000 | 77.247000000000000 | 0.000 | 6 |
| M\_8\_F\_lt\_Set2 | 1.490 | 0 | 0.0550000000000000 | 2225.754000000000 | 77.230999999999995 | 0.000 | 6 |
| M\_8\_F\_lt\_Set3 | 1.468 | 0 | 0.0730000000000000 | 2223.161000000000 | 74.638000000000005 | 0.000 | 9 |
| M\_8\_F\_lh\_Set1 | 1.504 | 0 | 0.0550000000000000 | 2209.621000000000 | 61.097999999999999 | 0.000 | 10 |
| M\_8\_F\_lh\_Set2 | 1.502 | 0 | 0.0550000000000000 | 2212.228000000000 | 63.706000000000003 | 0.000 | 11 |
| M\_8\_F\_lh\_Set3 | 1.479 | 0 | 0.0730000000000000 | 2209.644000000000 | 61.122000000000000 | 0.000 | 10 |
| M\_8\_F\_qp\_Set1 | 1.490 | 0 | 0.0550000000000000 | 2232.040000000000 | 83.516999999999996 | 0.000 | 10 |
| M\_8\_F\_qp\_Set2 | 1.472 | 0 | 0.0910000000000000 | 2234.660000000000 | 86.137000000000000 | 0.000 | 11 |
| M\_8\_F\_qp\_Set3 | 1.491 | 0 | 0.0550000000000000 | 2205.709000000000 | 57.186999999999998 | 0.000 | 11 |
| M\_8\_F\_qt\_Set1 | 1.515 | 0 | 0.0550000000000000 | 2213.360000000000 | 64.837000000000003 | 0.000 | 7 |
| M\_8\_F\_qt\_Set2 | 1.516 | 0 | 0.0550000000000000 | 2213.161000000000 | 64.638000000000005 | 0.000 | 8 |
| M\_8\_F\_qt\_Set3 | 1.515 | 0 | 0.0550000000000000 | 2210.902000000000 | 62.378999999999998 | 0.000 | 10 |
| M\_8\_F\_qh\_Set1 | 1.553 | 0 | 0.0550000000000000 | 2218.153000000000 | 69.629999999999995 | 0.000 | 12 |
| M\_8\_F\_qh\_Set2 | 1.554 | 0 | 0.0550000000000000 | 2212.220000000000 | 63.698000000000000 | 0.000 | 13 |
| M\_8\_F\_qh\_Set3 | 1.549 | 0 | 0.0550000000000000 | 2207.090000000000 | 58.567000000000000 | 0.000 | 12 |
| M\_8\_F\_pt\_Set1 | 1.516 | 0 | 0.1090000000000000 | 2222.839000000000 | 74.316000000000003 | 0.000 | 8 |
| M\_8\_F\_pt\_Set2 | 1.514 | 0 | 0.1090000000000000 | 2218.867000000000 | 70.343999999999994 | 0.000 | 9 |
| M\_8\_F\_pt\_Set3 | 1.543 | 0 | 0.0910000000000000 | 2208.713000000000 | 60.191000000000003 | 0.000 | 12 |
| M\_8\_F\_ph\_Set1 | 1.553 | 0 | 0.0550000000000000 | 2198.950000000000 | 50.427000000000000 | 0.000 | 9 |
| M\_8\_F\_ph\_Set2 | 1.552 | 0 | 0.0550000000000000 | 2198.002000000000 | 49.478999999999999 | 0.000 | 9 |
| M\_8\_F\_ph\_Set3 | 1.546 | 0 | 0.0730000000000000 | 2193.328000000000 | 44.805000000000000 | 0.000 | 10 |
| M\_8\_F\_th\_Set1 | 1.548 | 0 | 0.0550000000000000 | 2214.985000000000 | 66.462000000000003 | 0.000 | 9 |
| M\_8\_F\_th\_Set2 | 1.550 | 0 | 0.0550000000000000 | 2217.051000000000 | 68.528000000000006 | 0.000 | 10 |
| M\_8\_F\_th\_Set3 | 1.546 | 0 | 0.0730000000000000 | 2221.444000000000 | 72.921000000000006 | 0.000 | 13 |
| M\_8\_F\_lqp\_Set1 | 1.472 | 0 | 0.0550000000000000 | 2208.264000000000 | 59.741999999999997 | 0.000 | 8 |
| M\_8\_F\_lqp\_Set2 | 1.470 | 0 | 0.0550000000000000 | 2208.298000000000 | 59.774999999999999 | 0.000 | 8 |
| M\_8\_F\_lqp\_Set3 | 1.501 | 0 | 0.0550000000000000 | 2198.800000000000 | 50.277000000000001 | 0.000 | 9 |
| M\_8\_F\_lqt\_Set1 | 1.513 | 0 | 0.0550000000000000 | 2207.573000000000 | 59.051000000000002 | 0.000 | 9 |
| M\_8\_F\_lqt\_Set2 | 1.516 | 0 | 0.0550000000000000 | 2207.597000000000 | 59.073999999999998 | 0.000 | 9 |
| M\_8\_F\_lqt\_Set3 | 1.530 | 0 | 0.0360000000000000 | 2208.270000000000 | 59.747000000000000 | 0.000 | 10 |
| M\_8\_F\_lqh\_Set1 | 1.526 | 0 | 0.0550000000000000 | 2201.828000000000 | 53.305000000000000 | 0.000 | 11 |
| M\_8\_F\_lqh\_Set2 | 1.528 | 0 | 0.0550000000000000 | 2204.311000000000 | 55.789000000000001 | 0.000 | 12 |
| M\_8\_F\_lqh\_Set3 | 1.529 | 0 | 0.0550000000000000 | 2205.879000000000 | 57.356999999999999 | 0.000 | 11 |
| M\_8\_F\_lpt\_Set1 | 1.496 | 0 | 0.0910000000000000 | 2218.175000000000 | 69.653000000000006 | 0.000 | 9 |
| M\_8\_F\_lpt\_Set2 | 1.504 | 0 | 0.0910000000000000 | 2217.458000000000 | 68.935000000000002 | 0.000 | 10 |
| M\_8\_F\_lpt\_Set3 | 1.524 | 0 | 0.0910000000000000 | 2205.834000000000 | 57.311999999999998 | 0.000 | 11 |
| M\_8\_F\_lph\_Set1 | 1.550 | 0 | 0.0550000000000000 | 2202.603000000000 | 54.081000000000003 | 0.000 | 10 |
| M\_8\_F\_lph\_Set2 | 1.546 | 0 | 0.0550000000000000 | 2200.865000000000 | 52.343000000000004 | 0.000 | 10 |
| M\_8\_F\_lph\_Set3 | 1.543 | 0 | 0.0730000000000000 | 2186.266000000000 | 37.743000000000002 | 0.000 | 7 |
| M\_8\_F\_qpt\_Set1 | 1.546 | 0 | 0.1090000000000000 | 2216.867000000000 | 68.344999999999999 | 0.000 | 11 |
| M\_8\_F\_qpt\_Set2 | 1.529 | 0 | 0.1090000000000000 | 2219.167000000000 | 70.644999999999996 | 0.000 | 12 |
| M\_8\_F\_qpt\_Set3 | 1.535 | 0 | 0.0550000000000000 | 2199.508000000000 | 50.984999999999999 | 0.000 | 12 |
| M\_8\_F\_qph\_Set1 | 1.551 | 0 | 0.0550000000000000 | 2209.959000000000 | 61.436000000000000 | 0.000 | 12 |
| M\_8\_F\_qph\_Set2 | 1.554 | 0 | 0.0550000000000000 | 2207.290000000000 | 58.768000000000001 | 0.000 | 11 |
| M\_8\_F\_qph\_Set3 | 1.546 | 0 | 0.0730000000000000 | 2203.062000000000 | 54.539999999999999 | 0.000 | 12 |
| M\_8\_F\_qth\_Set1 | 1.544 | 0 | 0.0550000000000000 | 2211.238000000000 | 62.716000000000001 | 0.000 | 12 |
| M\_8\_F\_qth\_Set2 | 1.551 | 0 | 0.0550000000000000 | 2205.406000000000 | 56.884000000000000 | 0.000 | 13 |
| M\_8\_F\_qth\_Set3 | 1.548 | 0 | 0.0550000000000000 | 2194.913000000000 | 46.390999999999998 | 0.000 | 11 |
| M\_8\_F\_pth\_Set1 | 1.550 | 0 | 0.0550000000000000 | 2190.897000000000 | 42.375000000000000 | 0.000 | 9 |
| M\_8\_F\_pth\_Set2 | 1.545 | 0 | 0.0550000000000000 | 2197.474000000000 | 48.951999999999998 | 0.000 | 12 |
| M\_8\_F\_pth\_Set3 | 1.546 | 0 | 0.0730000000000000 | 2183.788000000000 | 35.265999999999998 | 0.000 | 9 |
| M\_8\_F\_lqpt\_Set1 | 1.528 | 0 | 0.0550000000000000 | 2199.800000000000 | 51.277000000000001 | 0.000 | 8 |
| M\_8\_F\_lqpt\_Set2 | 1.528 | 0 | 0.1090000000000000 | 2200.002000000000 | 51.479999999999997 | 0.000 | 8 |
| M\_8\_F\_lqpt\_Set3 | 1.528 | 0 | 0.0550000000000000 | 2194.005000000000 | 45.482999999999997 | 0.000 | 10 |
| M\_8\_F\_lqph\_Set1 | 1.549 | 0 | 0.0550000000000000 | 2206.351000000000 | 57.828000000000003 | 0.000 | 11 |
| M\_8\_F\_lqph\_Set2 | 1.549 | 0 | 0.0550000000000000 | 2208.765000000000 | 60.241999999999997 | 0.000 | 12 |
| M\_8\_F\_lqph\_Set3 | 1.554 | 0 | 0.0730000000000000 | 2194.302000000000 | 45.780000000000001 | 0.000 | 9 |
| M\_8\_F\_lqth\_Set1 | 1.530 | 0 | 0.0550000000000000 | 2197.047000000000 | 48.524999999999999 | 0.000 | 12 |
| M\_8\_F\_lqth\_Set2 | 1.525 | 0 | 0.0550000000000000 | 2196.995000000000 | 48.472000000000001 | 0.000 | 12 |
| M\_8\_F\_lqth\_Set3 | 1.532 | 0 | 0.0550000000000000 | 2193.726000000000 | 45.203000000000003 | 0.000 | 11 |
| M\_8\_F\_lpth\_Set1 | 1.549 | 0 | 0.0550000000000000 | 2196.462000000000 | 47.939999999999998 | 0.000 | 11 |
| M\_8\_F\_lpth\_Set2 | 1.550 | 0 | 0.0550000000000000 | 2197.039000000000 | 48.517000000000003 | 0.000 | 12 |
| M\_8\_F\_lpth\_Set3 | 1.545 | 0 | 0.0730000000000000 | 2188.018000000000 | 39.494999999999997 | 0.000 | 10 |
| M\_8\_F\_lqpth\_Set1 | 1.547 | 0 | 0.0550000000000000 | 2196.918000000000 | 48.396000000000001 | 0.000 | 11 |
| M\_8\_F\_lqpth\_Set2 | 1.548 | 0 | 0.0550000000000000 | 2199.620000000000 | 51.097999999999999 | 0.000 | 12 |
| M\_8\_F\_lqpth\_Set3 | 1.553 | 0 | 0.0730000000000000 | 2195.865000000000 | 47.341999999999999 | 0.000 | 12 |
| M\_10\_F\_l\_Set1 | 1.485 | 0 | 0.0550000000000000 | 2278.332000000000 | 129.810000000000002 | 0.000 | 4 |
| M\_10\_F\_l\_Set2 | 1.489 | 0 | 0.0550000000000000 | 2278.302000000000 | 129.780000000000001 | 0.000 | 4 |
| M\_10\_F\_l\_Set3 | 1.468 | 0 | 0.0730000000000000 | 2276.043000000000 | 127.521000000000001 | 0.000 | 4 |
| M\_10\_F\_q\_Set1 | 1.494 | 0 | 0.0730000000000000 | 2260.283000000000 | 111.760999999999996 | 0.000 | 5 |
| M\_10\_F\_q\_Set2 | 1.497 | 0 | 0.0730000000000000 | 2253.902000000000 | 105.379999999999995 | 0.000 | 6 |
| M\_10\_F\_q\_Set3 | 1.512 | 0 | 0.0550000000000000 | 2238.809000000000 | 90.286000000000001 | 0.000 | 7 |
| M\_10\_F\_p\_Set1 | 1.475 | 0 | 0.0550000000000000 | 2260.131000000000 | 111.608999999999995 | 0.000 | 7 |
| M\_10\_F\_p\_Set2 | 1.481 | 0 | 0.0730000000000000 | 2252.309000000000 | 103.787000000000006 | 0.000 | 8 |
| M\_10\_F\_p\_Set3 | 1.460 | 0 | 0.0910000000000000 | 2221.864000000000 | 73.340999999999994 | 0.000 | 10 |
| M\_10\_F\_t\_Set1 | 1.291 | 0 | 0.0360000000000000 | 2268.547000000000 | 120.025000000000006 | 0.000 | 6 |
| M\_10\_F\_t\_Set2 | 1.294 | 0 | 0.0360000000000000 | 2268.526000000000 | 120.003000000000000 | 0.000 | 6 |
| M\_10\_F\_t\_Set3 | 1.330 | 0 | 0.0180000000000000 | 2255.168000000000 | 106.646000000000001 | 0.000 | 4 |
| M\_10\_F\_h\_Set1 | 1.524 | 0 | 0.0550000000000000 | 2248.556000000000 | 100.033000000000001 | 0.000 | 11 |
| M\_10\_F\_h\_Set2 | 1.533 | 0 | 0.0550000000000000 | 2251.154000000000 | 102.631000000000000 | 0.000 | 12 |
| M\_10\_F\_h\_Set3 | 1.535 | 0 | 0.0730000000000000 | 2242.029000000000 | 93.506000000000000 | 0.000 | 8 |
| M\_10\_F\_lq\_Set1 | 1.517 | 0 | 0.0550000000000000 | 2225.242000000000 | 76.718999999999994 | 0.000 | 7 |
| M\_10\_F\_lq\_Set2 | 1.513 | 0 | 0.0550000000000000 | 2225.205000000000 | 76.682000000000002 | 0.000 | 7 |
| M\_10\_F\_lq\_Set3 | 1.516 | 0 | 0.0360000000000000 | 2223.983000000000 | 75.460999999999999 | 0.000 | 6 |
| M\_10\_F\_lp\_Set1 | 1.444 | 0 | 0.0550000000000000 | 2252.764000000000 | 104.241000000000000 | 0.000 | 6 |
| M\_10\_F\_lp\_Set2 | 1.472 | 0 | 0.0550000000000000 | 2247.992000000000 | 99.468999999999994 | 0.000 | 7 |
| M\_10\_F\_lp\_Set3 | 1.462 | 0 | 0.0730000000000000 | 2213.383000000000 | 64.859999999999999 | 0.000 | 8 |
| M\_10\_F\_lt\_Set1 | 1.495 | 0 | 0.0550000000000000 | 2242.734000000000 | 94.210999999999999 | 0.000 | 6 |
| M\_10\_F\_lt\_Set2 | 1.481 | 0 | 0.0550000000000000 | 2242.668000000000 | 94.146000000000001 | 0.000 | 6 |
| M\_10\_F\_lt\_Set3 | 1.464 | 0 | 0.0730000000000000 | 2241.699000000000 | 93.177000000000007 | 0.000 | 9 |
| M\_10\_F\_lh\_Set1 | 1.486 | 0 | 0.0550000000000000 | 2228.544000000000 | 80.021000000000001 | 0.000 | 8 |
| M\_10\_F\_lh\_Set2 | 1.480 | 0 | 0.0550000000000000 | 2230.975000000000 | 82.451999999999998 | 0.000 | 9 |
| M\_10\_F\_lh\_Set3 | 1.460 | 0 | 0.0730000000000000 | 2228.719000000000 | 80.195999999999998 | 0.000 | 8 |
| M\_10\_F\_qp\_Set1 | 1.473 | 0 | 0.0550000000000000 | 2240.965000000000 | 92.441999999999993 | 0.000 | 9 |
| M\_10\_F\_qp\_Set2 | 1.475 | 0 | 0.0730000000000000 | 2241.317000000000 | 92.795000000000002 | 0.000 | 9 |
| M\_10\_F\_qp\_Set3 | 1.477 | 0 | 0.0360000000000000 | 2209.308000000000 | 60.784999999999997 | 0.000 | 9 |
| M\_10\_F\_qt\_Set1 | 1.488 | 0 | 0.0730000000000000 | 2228.151000000000 | 79.628000000000000 | 0.000 | 6 |
| M\_10\_F\_qt\_Set2 | 1.493 | 0 | 0.0730000000000000 | 2228.966000000000 | 80.444000000000003 | 0.000 | 7 |
| M\_10\_F\_qt\_Set3 | 1.512 | 0 | 0.0550000000000000 | 2221.832000000000 | 73.310000000000002 | 0.000 | 8 |
| M\_10\_F\_qh\_Set1 | 1.533 | 0 | 0.0550000000000000 | 2231.779000000000 | 83.257000000000005 | 0.000 | 9 |
| M\_10\_F\_qh\_Set2 | 1.535 | 0 | 0.0550000000000000 | 2227.576000000000 | 79.054000000000002 | 0.000 | 10 |
| M\_10\_F\_qh\_Set3 | 1.514 | 0 | 0.0550000000000000 | 2229.897000000000 | 81.375000000000000 | 0.000 | 12 |
| M\_10\_F\_pt\_Set1 | 1.487 | 0 | 0.0550000000000000 | 2237.356000000000 | 88.832999999999998 | 0.000 | 7 |
| M\_10\_F\_pt\_Set2 | 1.479 | 0 | 0.0730000000000000 | 2231.233000000000 | 82.709999999999994 | 0.000 | 8 |
| M\_10\_F\_pt\_Set3 | 1.511 | 0 | 0.0910000000000000 | 2212.992000000000 | 64.469999999999999 | 0.000 | 9 |
| M\_10\_F\_ph\_Set1 | 1.527 | 0 | 0.0550000000000000 | 2218.623000000000 | 70.099999999999994 | 0.000 | 8 |
| M\_10\_F\_ph\_Set2 | 1.534 | 0 | 0.0550000000000000 | 2228.230000000000 | 79.706999999999994 | 0.000 | 12 |
| M\_10\_F\_ph\_Set3 | 1.532 | 0 | 0.0730000000000000 | 2209.177000000000 | 60.655000000000001 | 0.000 | 11 |
| M\_10\_F\_th\_Set1 | 1.526 | 0 | 0.0550000000000000 | 2231.259000000000 | 82.736999999999995 | 0.000 | 7 |
| M\_10\_F\_th\_Set2 | 1.530 | 0 | 0.0550000000000000 | 2231.250000000000 | 82.727000000000004 | 0.000 | 7 |
| M\_10\_F\_th\_Set3 | 1.534 | 0 | 0.0730000000000000 | 2227.886000000000 | 79.364000000000004 | 0.000 | 7 |
| M\_10\_F\_lqp\_Set1 | 1.440 | 0 | 0.0550000000000000 | 2216.859000000000 | 68.337000000000003 | 0.000 | 7 |
| M\_10\_F\_lqp\_Set2 | 1.473 | 0 | 0.0550000000000000 | 2216.857000000000 | 68.334000000000003 | 0.000 | 7 |
| M\_10\_F\_lqp\_Set3 | 1.467 | 0 | 0.0550000000000000 | 2200.851000000000 | 52.328000000000003 | 0.000 | 7 |
| M\_10\_F\_lqt\_Set1 | 1.509 | 0 | 0.0550000000000000 | 2214.250000000000 | 65.727000000000004 | 0.000 | 6 |
| M\_10\_F\_lqt\_Set2 | 1.517 | 0 | 0.0550000000000000 | 2214.247000000000 | 65.724000000000004 | 0.000 | 6 |
| M\_10\_F\_lqt\_Set3 | 1.517 | 0 | 0.0360000000000000 | 2217.457000000000 | 68.935000000000002 | 0.000 | 8 |
| M\_10\_F\_lqh\_Set1 | 1.513 | 0 | 0.0550000000000000 | 2229.187000000000 | 80.665000000000006 | 0.000 | 12 |
| M\_10\_F\_lqh\_Set2 | 1.512 | 0 | 0.0550000000000000 | 2226.311000000000 | 77.789000000000001 | 0.000 | 11 |
| M\_10\_F\_lqh\_Set3 | 1.515 | 0 | 0.0360000000000000 | 2225.024000000000 | 76.501000000000005 | 0.000 | 9 |
| M\_10\_F\_lpt\_Set1 | 1.440 | 0 | 0.0550000000000000 | 2238.551000000000 | 90.028000000000006 | 0.000 | 10 |
| M\_10\_F\_lpt\_Set2 | 1.475 | 0 | 0.0550000000000000 | 2232.021000000000 | 83.498999999999995 | 0.000 | 9 |
| M\_10\_F\_lpt\_Set3 | 1.466 | 0 | 0.0730000000000000 | 2211.281000000000 | 62.758000000000003 | 0.000 | 9 |
| M\_10\_F\_lph\_Set1 | 1.537 | 0 | 0.0550000000000000 | 2218.942000000000 | 70.418999999999997 | 0.000 | 8 |
| M\_10\_F\_lph\_Set2 | 1.532 | 0 | 0.0550000000000000 | 2217.295000000000 | 68.772999999999996 | 0.000 | 8 |
| M\_10\_F\_lph\_Set3 | 1.530 | 0 | 0.0730000000000000 | 2202.528000000000 | 54.006000000000000 | 0.000 | 8 |
| M\_10\_F\_qpt\_Set1 | 1.486 | 0 | 0.0550000000000000 | 2224.252000000000 | 75.730000000000004 | 0.000 | 8 |
| M\_10\_F\_qpt\_Set2 | 1.483 | 0 | 0.0730000000000000 | 2223.846000000000 | 75.322999999999993 | 0.000 | 8 |
| M\_10\_F\_qpt\_Set3 | 1.473 | 0 | 0.0360000000000000 | 2209.246000000000 | 60.723999999999997 | 0.000 | 11 |
| M\_10\_F\_qph\_Set1 | 1.531 | 0 | 0.0550000000000000 | 2227.951000000000 | 79.427999999999997 | 0.000 | 11 |
| M\_10\_F\_qph\_Set2 | 1.527 | 0 | 0.0550000000000000 | 2225.506000000000 | 76.983000000000004 | 0.000 | 10 |
| M\_10\_F\_qph\_Set3 | 1.533 | 0 | 0.0730000000000000 | 2209.321000000000 | 60.798000000000002 | 0.000 | 9 |
| M\_10\_F\_qth\_Set1 | 1.535 | 0 | 0.0550000000000000 | 2222.031000000000 | 73.507999999999996 | 0.000 | 8 |
| M\_10\_F\_qth\_Set2 | 1.529 | 0 | 0.0550000000000000 | 2218.187000000000 | 69.665000000000006 | 0.000 | 9 |
| M\_10\_F\_qth\_Set3 | 1.508 | 0 | 0.0550000000000000 | 2214.406000000000 | 65.882999999999996 | 0.000 | 10 |
| M\_10\_F\_pth\_Set1 | 1.529 | 0 | 0.0550000000000000 | 2217.276000000000 | 68.753000000000000 | 0.000 | 11 |
| M\_10\_F\_pth\_Set2 | 1.532 | 0 | 0.0550000000000000 | 2219.069000000000 | 70.546999999999997 | 0.000 | 12 |
| M\_10\_F\_pth\_Set3 | 1.536 | 0 | 0.0730000000000000 | 2201.026000000000 | 52.503000000000000 | 0.000 | 10 |
| M\_10\_F\_lqpt\_Set1 | 1.437 | 0 | 0.0550000000000000 | 2211.347000000000 | 62.825000000000003 | 0.000 | 8 |
| M\_10\_F\_lqpt\_Set2 | 1.461 | 0 | 0.0550000000000000 | 2210.786000000000 | 62.262999999999998 | 0.000 | 8 |
| M\_10\_F\_lqpt\_Set3 | 1.469 | 0 | 0.0550000000000000 | 2202.211000000000 | 53.689000000000000 | 0.000 | 9 |
| M\_10\_F\_lqph\_Set1 | 1.525 | 0 | 0.0550000000000000 | 2218.972000000000 | 70.448999999999998 | 0.000 | 8 |
| M\_10\_F\_lqph\_Set2 | 1.531 | 0 | 0.0550000000000000 | 2216.807000000000 | 68.284000000000006 | 0.000 | 7 |
| M\_10\_F\_lqph\_Set3 | 1.539 | 0 | 0.0730000000000000 | 2200.866000000000 | 52.344000000000001 | 0.000 | 7 |
| M\_10\_F\_lqth\_Set1 | 1.516 | 0 | 0.0550000000000000 | 2212.101000000000 | 63.578000000000003 | 0.000 | 9 |
| M\_10\_F\_lqth\_Set2 | 1.506 | 0 | 0.0550000000000000 | 2212.159000000000 | 63.636000000000003 | 0.000 | 9 |
| M\_10\_F\_lqth\_Set3 | 1.514 | 0 | 0.0360000000000000 | 2219.187000000000 | 70.664000000000001 | 0.000 | 12 |
| M\_10\_F\_lpth\_Set1 | 1.528 | 0 | 0.0550000000000000 | 2214.080000000000 | 65.557000000000002 | 0.000 | 9 |
| M\_10\_F\_lpth\_Set2 | 1.531 | 0 | 0.0550000000000000 | 2212.927000000000 | 64.403999999999996 | 0.000 | 10 |
| M\_10\_F\_lpth\_Set3 | 1.531 | 0 | 0.0730000000000000 | 2201.634000000000 | 53.112000000000002 | 0.000 | 9 |
| M\_10\_F\_lqpth\_Set1 | 1.531 | 0 | 0.0550000000000000 | 2211.144000000000 | 62.621000000000002 | 0.000 | 9 |
| M\_10\_F\_lqpth\_Set2 | 1.530 | 0 | 0.0550000000000000 | 2210.899000000000 | 62.375999999999998 | 0.000 | 9 |
| M\_10\_F\_lqpth\_Set3 | 1.531 | 0 | 0.0730000000000000 | 2202.302000000000 | 53.779000000000003 | 0.000 | 9 |
