# Supplementary figures and images for: Assessing the current and future potential geographic distribution of the American dog tick, Dermacentor variabilis (Say) (Acari: Ixodidae) in North America
Source: PLoS One. 2020 Aug 10;15(8):e0237191. doi: 10.1371/journal.pone.0237191 (PMC7416948; doi:10.1371/journal.pone.0237191)

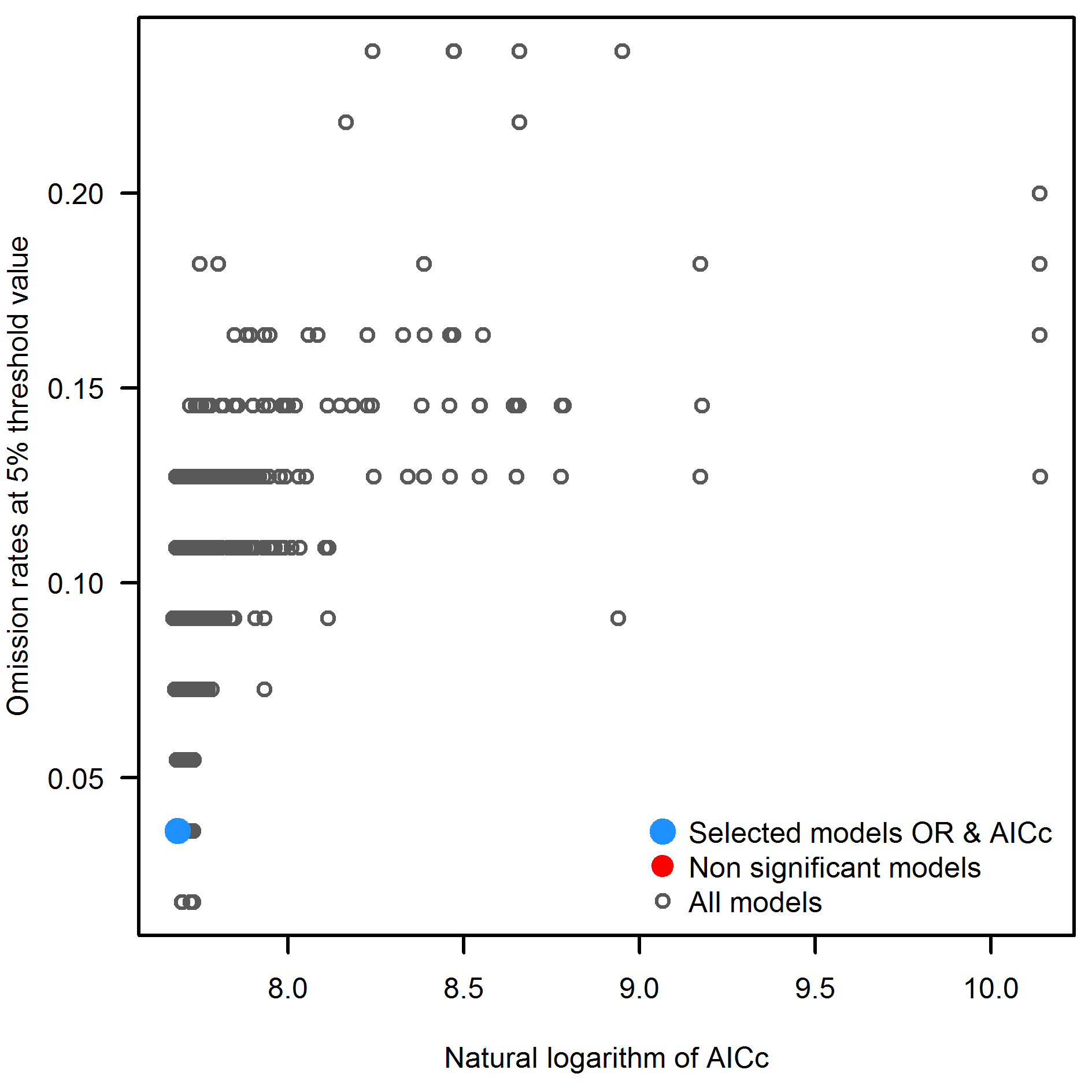

Supplement: S3 File — (ZIP) [file pone.0237191.s003.zip › SF3/evaluation_figure.png]
