# Supplementary material for: Assessing the current and future potential geographic distribution of the American dog tick, Dermacentor variabilis (Say) (Acari: Ixodidae) in North America
Source: PLoS One. 2020 Aug 10;15(8):e0237191. doi: 10.1371/journal.pone.0237191 (PMC7416948; doi:10.1371/journal.pone.0237191)

**Table 1. Variable contribution to final model**

| Variable | Percent contribution | Permutation importance |
| --- | --- | --- |
| BIO1 Annual Mean Temperature | 37.5 | 19.7 |
| BIO16 Precipitation of Wettest Quarter | 36.7 | 9.6 |
| BIO12 Annual Precipitation | 16.3 | 23.1 |
| BIO5 Max Temperature of Warmest Month | 3.1 | 16.5 |
| BIO6 Min Temperature of Coldest Month | 2.0 | 12 |
| BIO13 Precipitation of Wettest Month | 1.2 | 2.2 |
| BIO15 Precipitation Seasonality (Coefficient of Variation) | 1.1 | 7.9 |
| BIO10 Mean Temperature of Warmest Quarter | 0.8 | 5.9 |
| BIO3 Isothermality (BIO2/BIO7) (* 100) | 0.6 | 0.7 |
| BIO14 Precipitation of Driest Month | 0.4 | 1.2 |
| BIO2 Mean Diurnal Range (Mean of monthly (max temp - min temp)) | 0.2 | 1.3 |
| BIO17 Precipitation of Driest Quarter | 0.1 | 0 |

**Variable response curves**


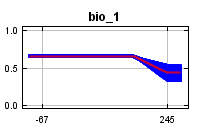


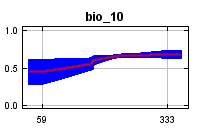


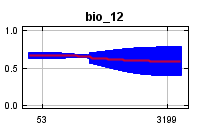


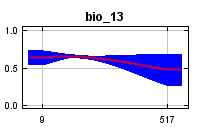


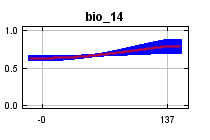


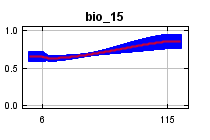


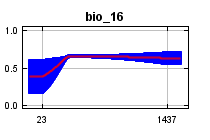


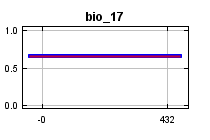


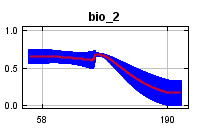


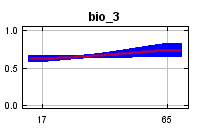


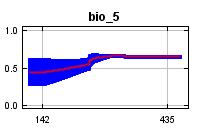


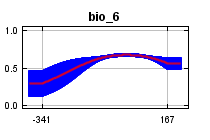

Supplement: S4 File — (DOCX) [file pone.0237191.s004.docx]
